# Supplementary figures and images for: DNGR-1 signalling limits dendritic cell activation for optimal antigen cross-presentation (part 1 of 2)
Source: EMBO J. 2025 Oct 29;44(23):6857–91. doi: 10.1038/s44318-025-00620-z (PMC12669754; doi:10.1038/s44318-025-00620-z)

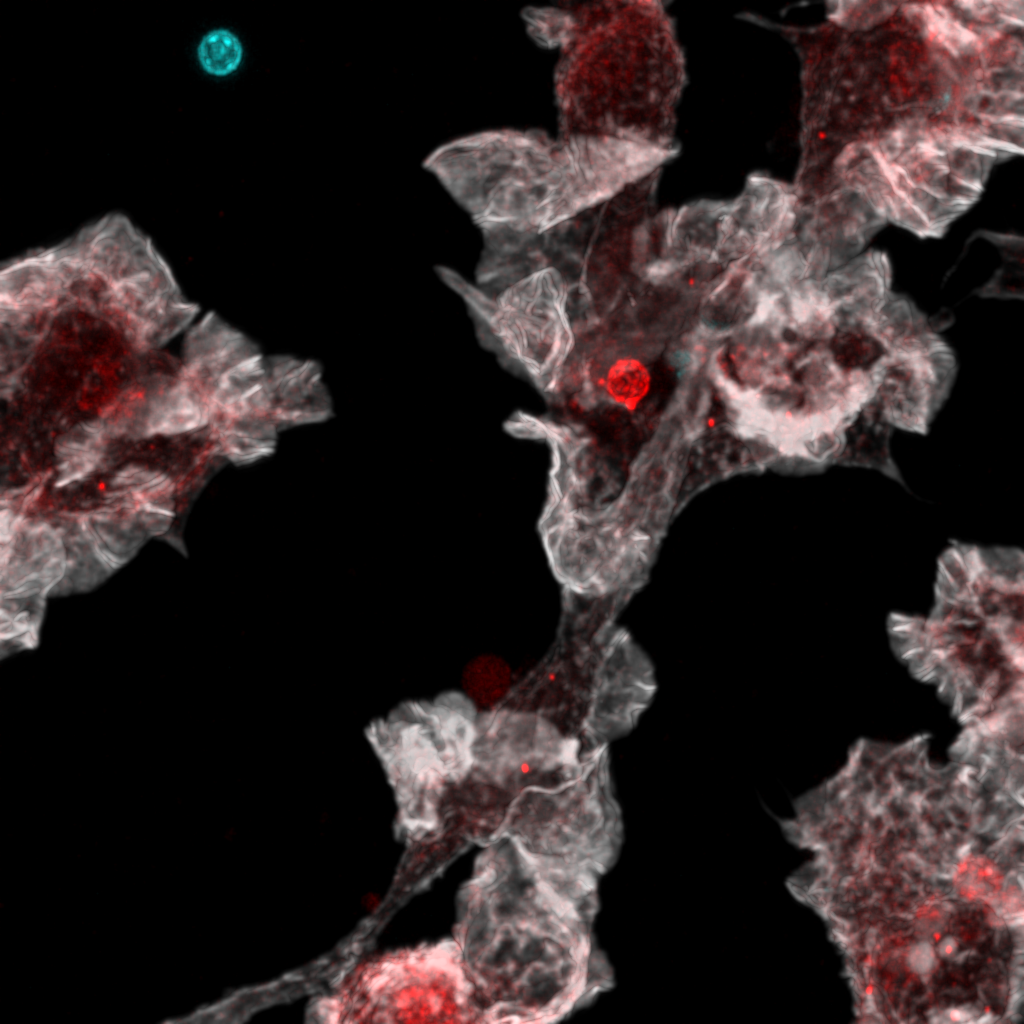

Supplement: Supplementary file 2 — Source data Fig. 1 [file 44318_2025_620_MOESM2_ESM.zip › Figure 1/1G/C9 LMC_2 Zoom 2-Deconvolv-Gauss-02_Maximum intensity projection_c2+4+5.tif]

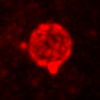

Supplement: Supplementary file 2 — Source data Fig. 1 [file 44318_2025_620_MOESM2_ESM.zip › Figure 1/1G/C9 LMC_2 Zoom 2-Deconvolv-Gauss-02_Maximum intensity projection_c5 copy.tif]

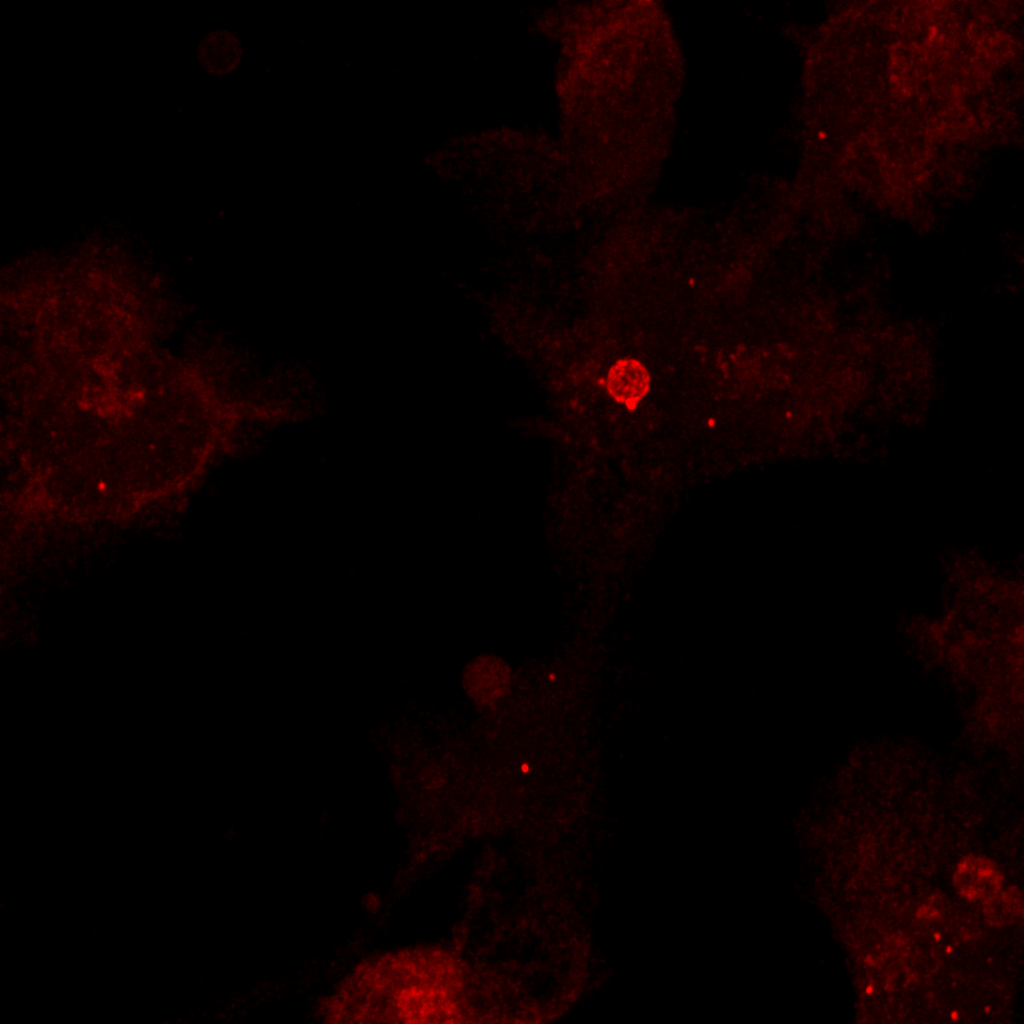

Supplement: Supplementary file 2 — Source data Fig. 1 [file 44318_2025_620_MOESM2_ESM.zip › Figure 1/1G/C9 LMC_2 Zoom 2-Deconvolv-Gauss-02_Maximum intensity projection_c5.tif]

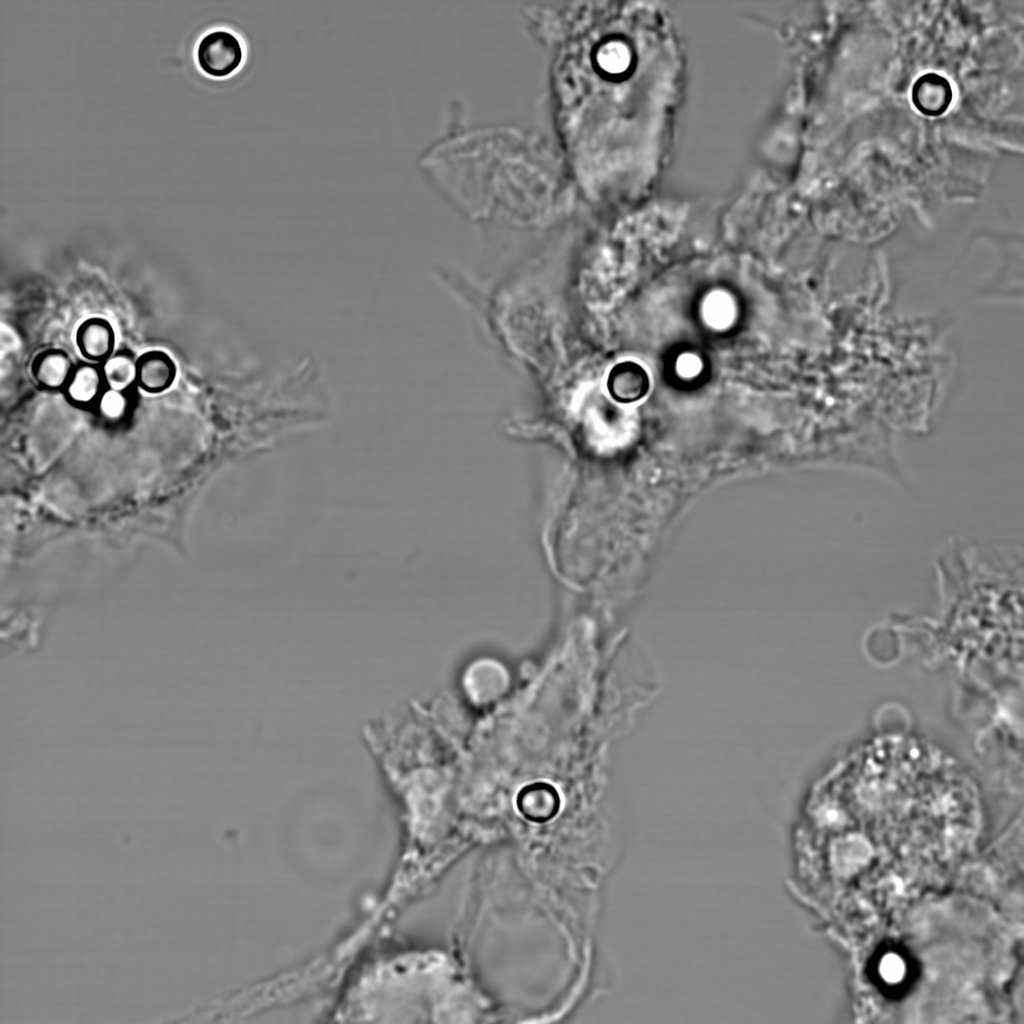

Supplement: Supplementary file 2 — Source data Fig. 1 [file 44318_2025_620_MOESM2_ESM.zip › Figure 1/1G/C9 LMC_2 Zoom 2-Deconvolv-Gauss-02_z08c3.tif]

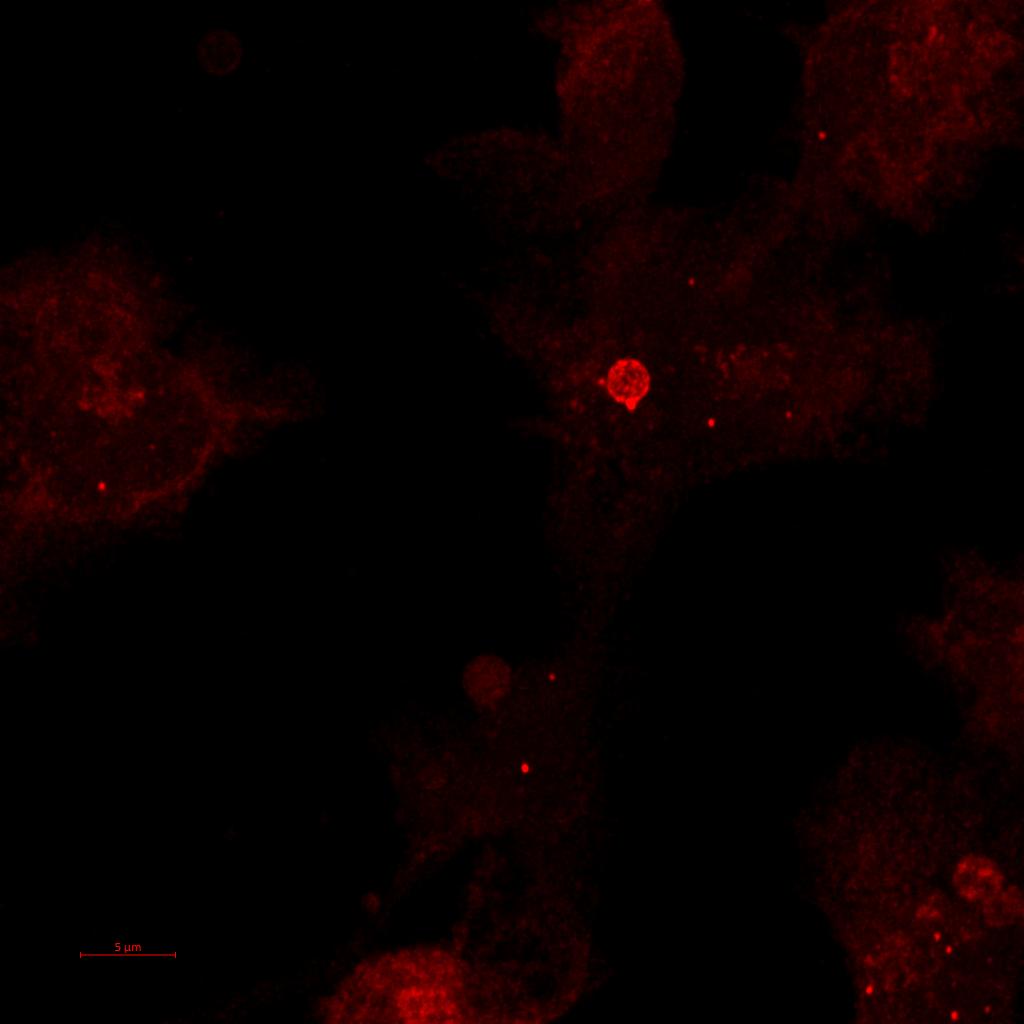

Supplement: Supplementary file 2 — Source data Fig. 1 [file 44318_2025_620_MOESM2_ESM.zip › Figure 1/1G/C9 LMC_2 Zoom 2-Measure_c5.tif]

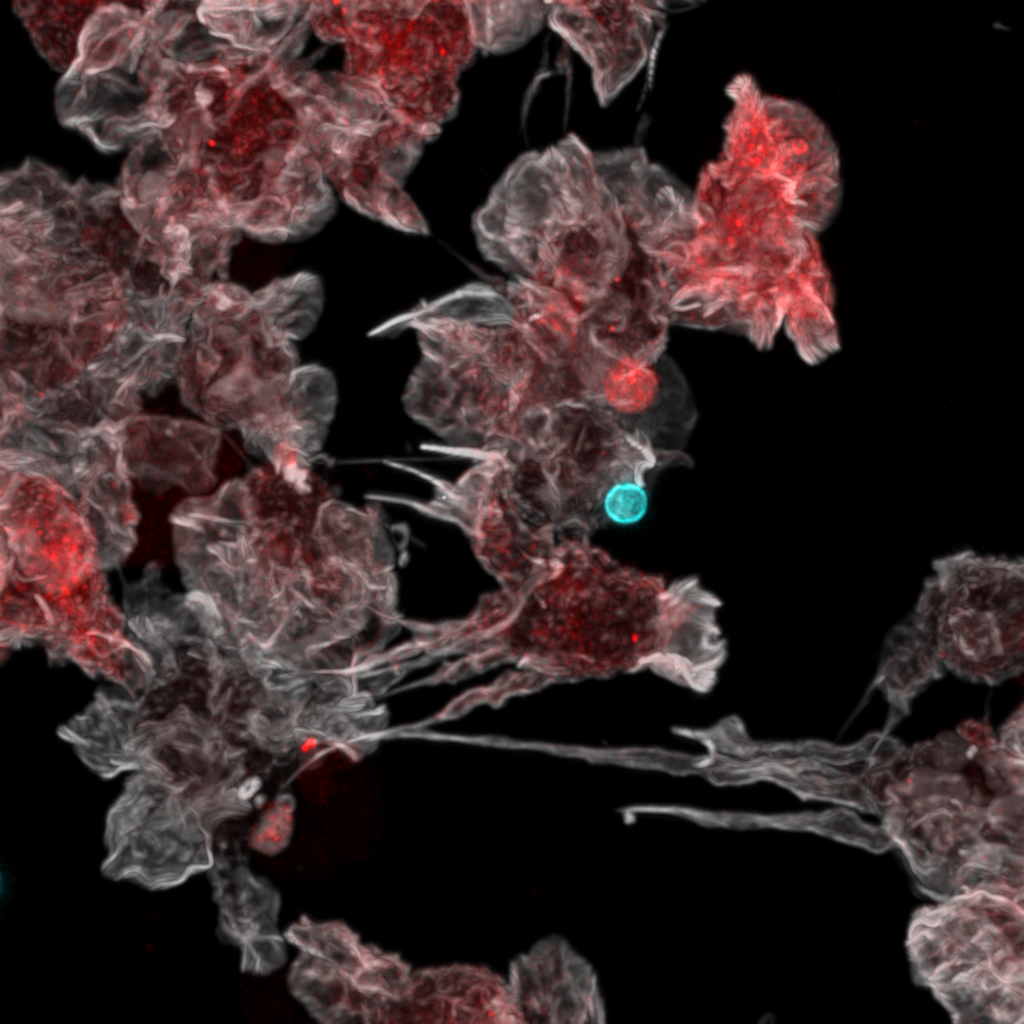

Supplement: Supplementary file 2 — Source data Fig. 1 [file 44318_2025_620_MOESM2_ESM.zip › Figure 1/1G/KO LMC_5 Zoom 2-Deconvolv-Gauss-02_Maximum intensity projection_c2+4+5.tif]

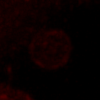

Supplement: Supplementary file 2 — Source data Fig. 1 [file 44318_2025_620_MOESM2_ESM.zip › Figure 1/1G/KO LMC_5 Zoom 2-Deconvolv-Gauss-02_Maximum intensity projection_c5 copy.tif]

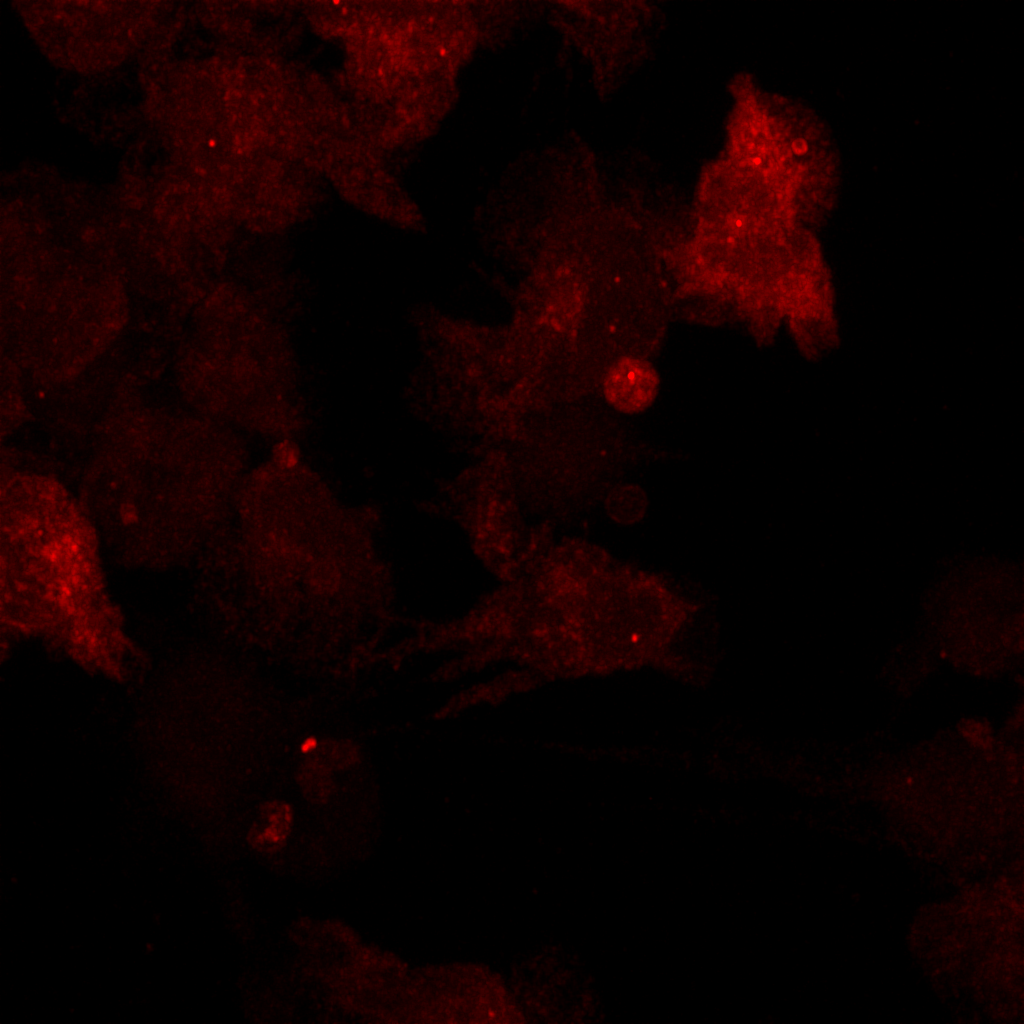

Supplement: Supplementary file 2 — Source data Fig. 1 [file 44318_2025_620_MOESM2_ESM.zip › Figure 1/1G/KO LMC_5 Zoom 2-Deconvolv-Gauss-02_Maximum intensity projection_c5.tif]

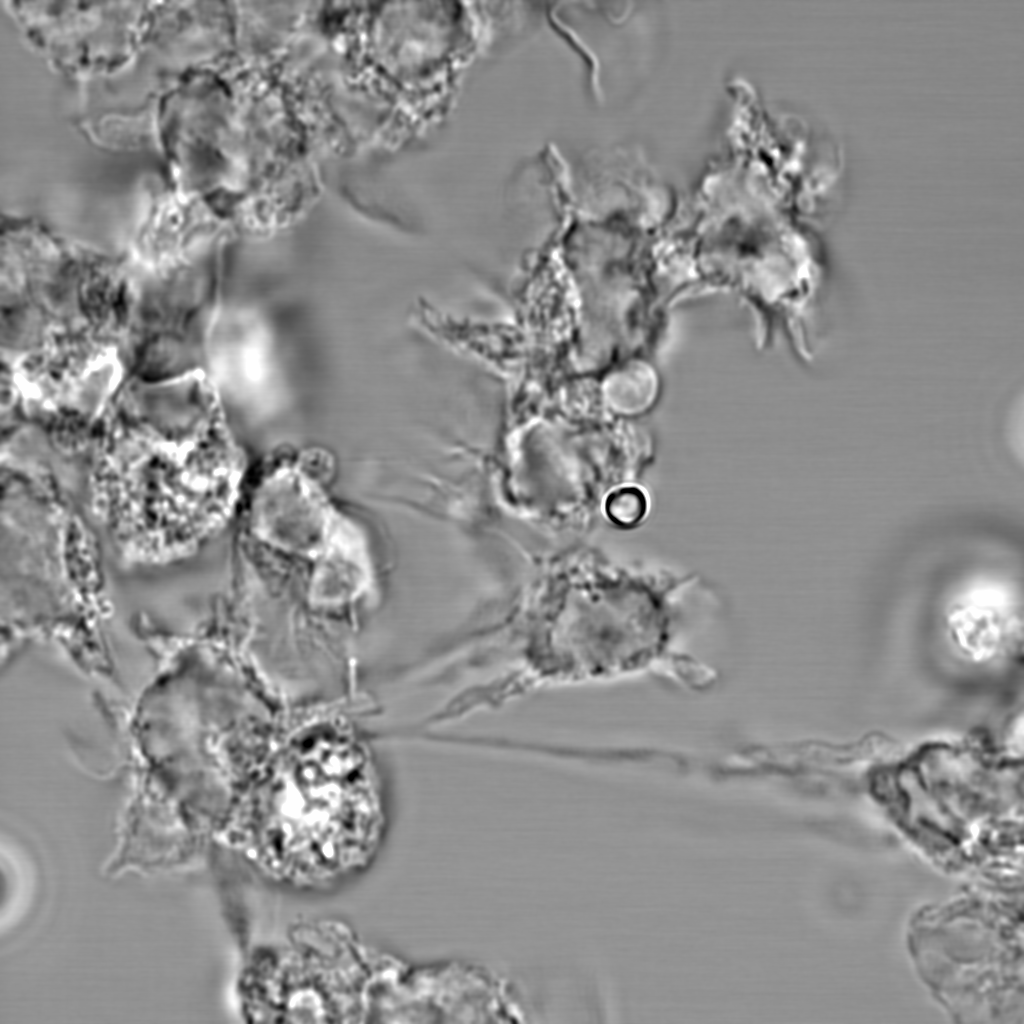

Supplement: Supplementary file 2 — Source data Fig. 1 [file 44318_2025_620_MOESM2_ESM.zip › Figure 1/1G/KO LMC_5 Zoom 2-Deconvolv-Gauss-02_z14c3.tif]

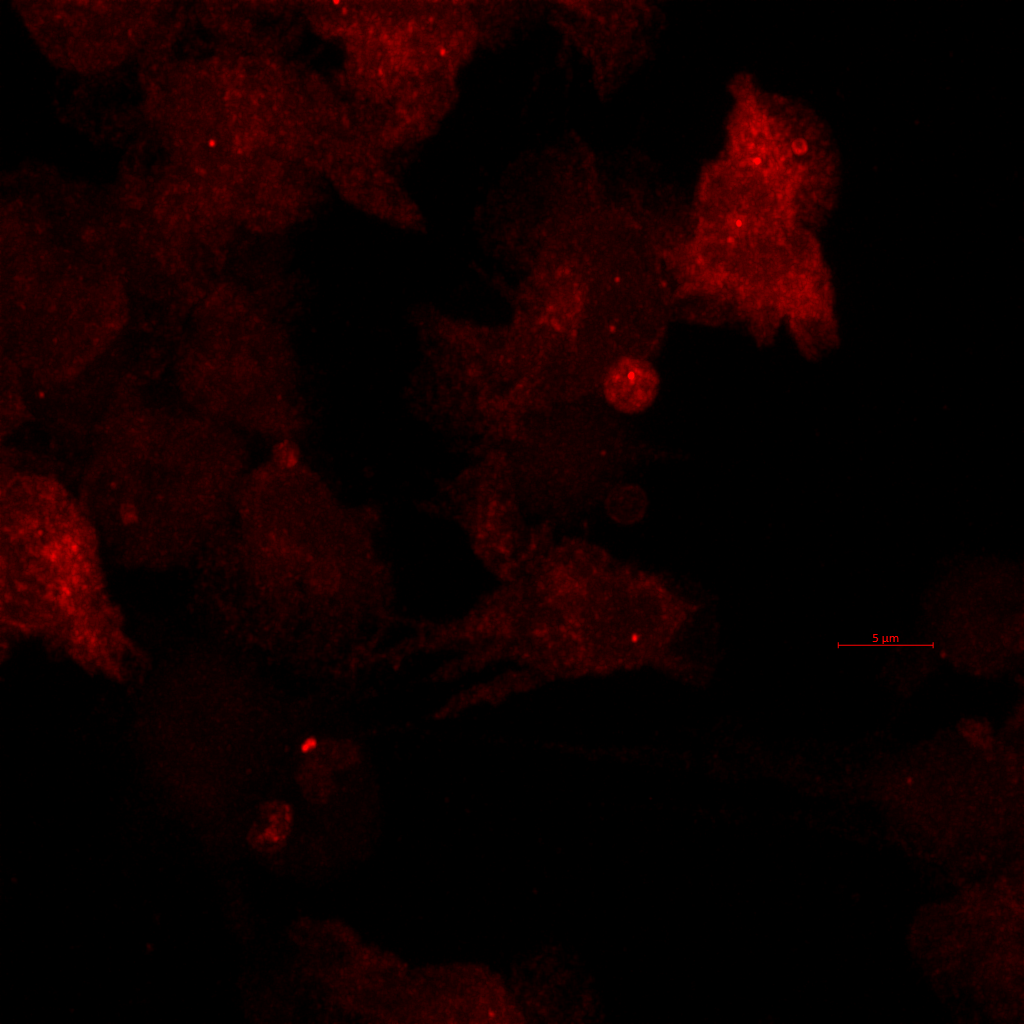

Supplement: Supplementary file 2 — Source data Fig. 1 [file 44318_2025_620_MOESM2_ESM.zip › Figure 1/1G/KO LMC_5 Zoom 2-Measure_c5.tif]

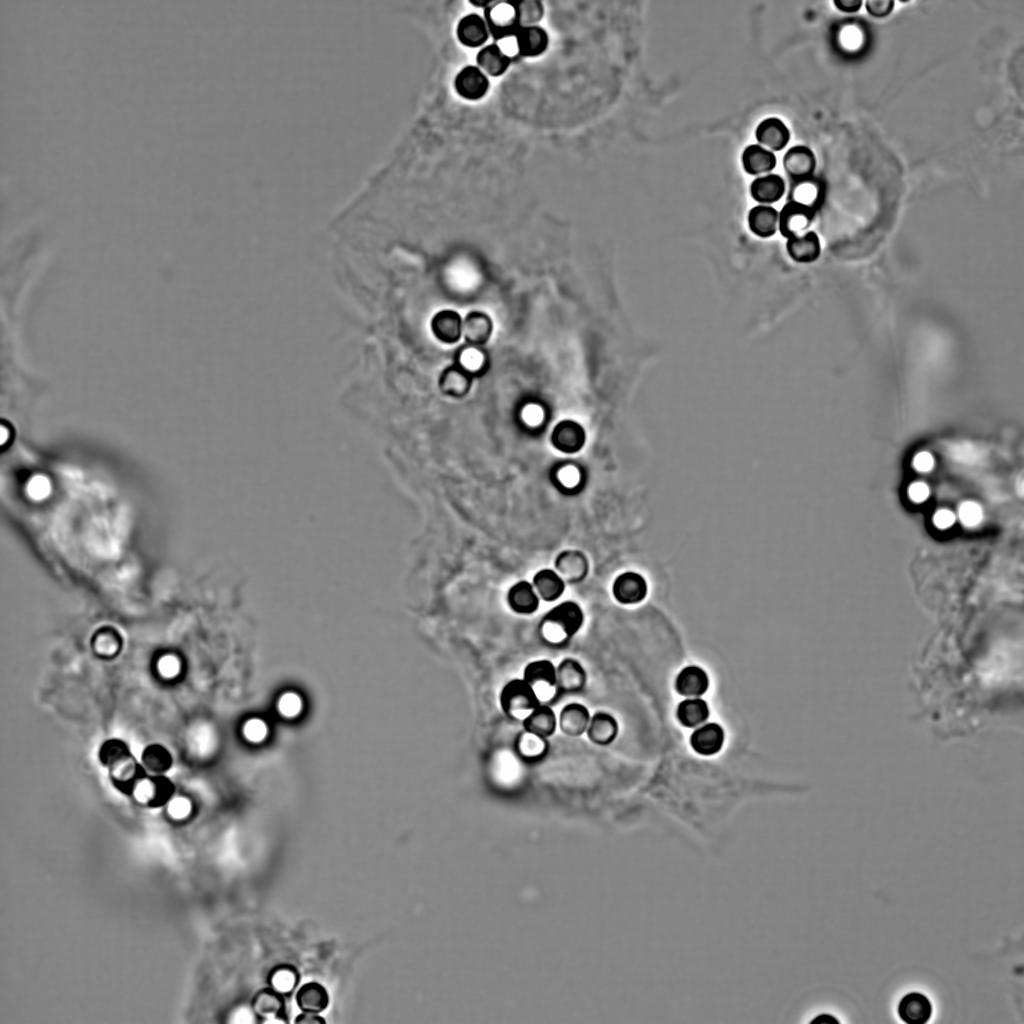

Supplement: Supplementary file 2 — Source data Fig. 1 [file 44318_2025_620_MOESM2_ESM.zip › Figure 1/1J/PSyk525 15 min C9-Deconvolv-01-Gauss-02_z16c3.tif]

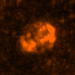

Supplement: Supplementary file 2 — Source data Fig. 1 [file 44318_2025_620_MOESM2_ESM.zip › Figure 1/1J/PSyk525 15 min C9-Deconvolv-01-Gauss-02-Create Image Subset-01_Maximum intensity projection_c2 copy.tif]

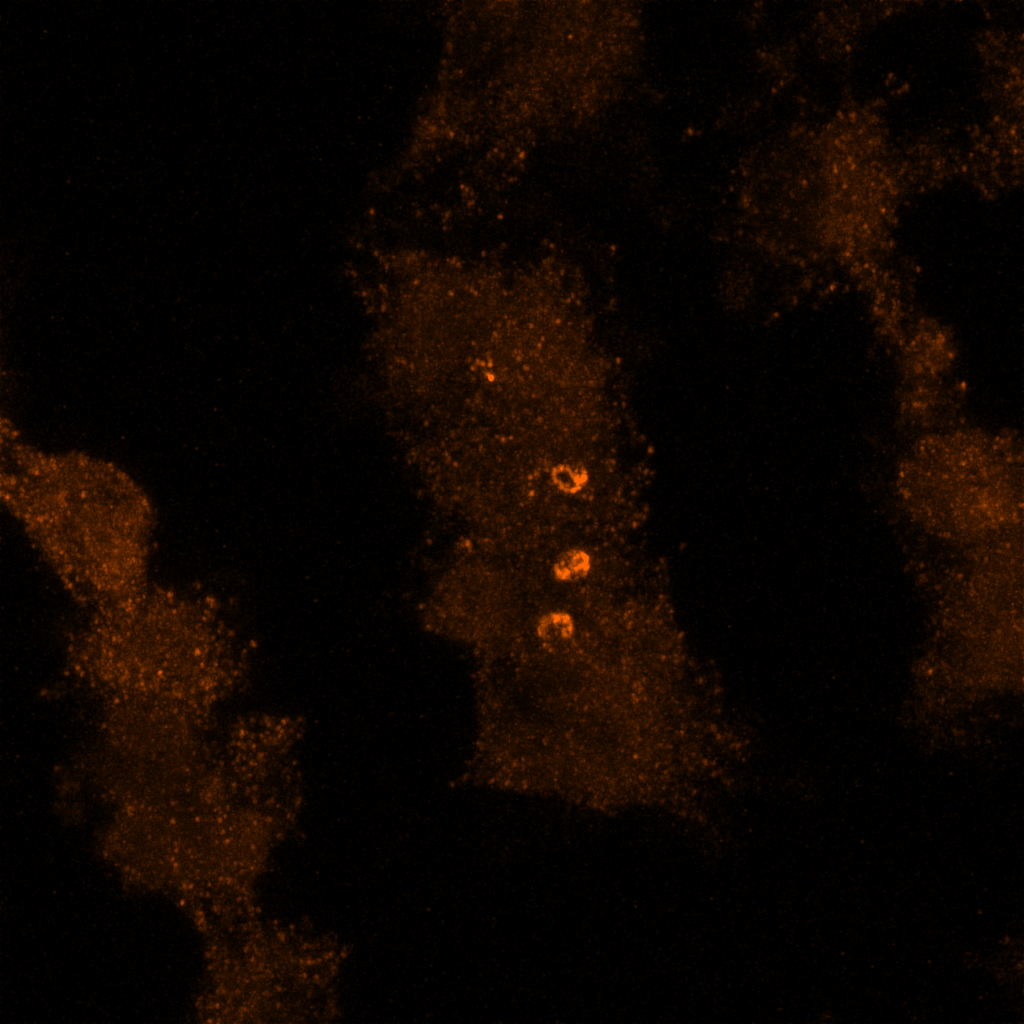

Supplement: Supplementary file 2 — Source data Fig. 1 [file 44318_2025_620_MOESM2_ESM.zip › Figure 1/1J/PSyk525 15 min C9-Deconvolv-01-Gauss-02-Create Image Subset-01_Maximum intensity projection_c2.tif]

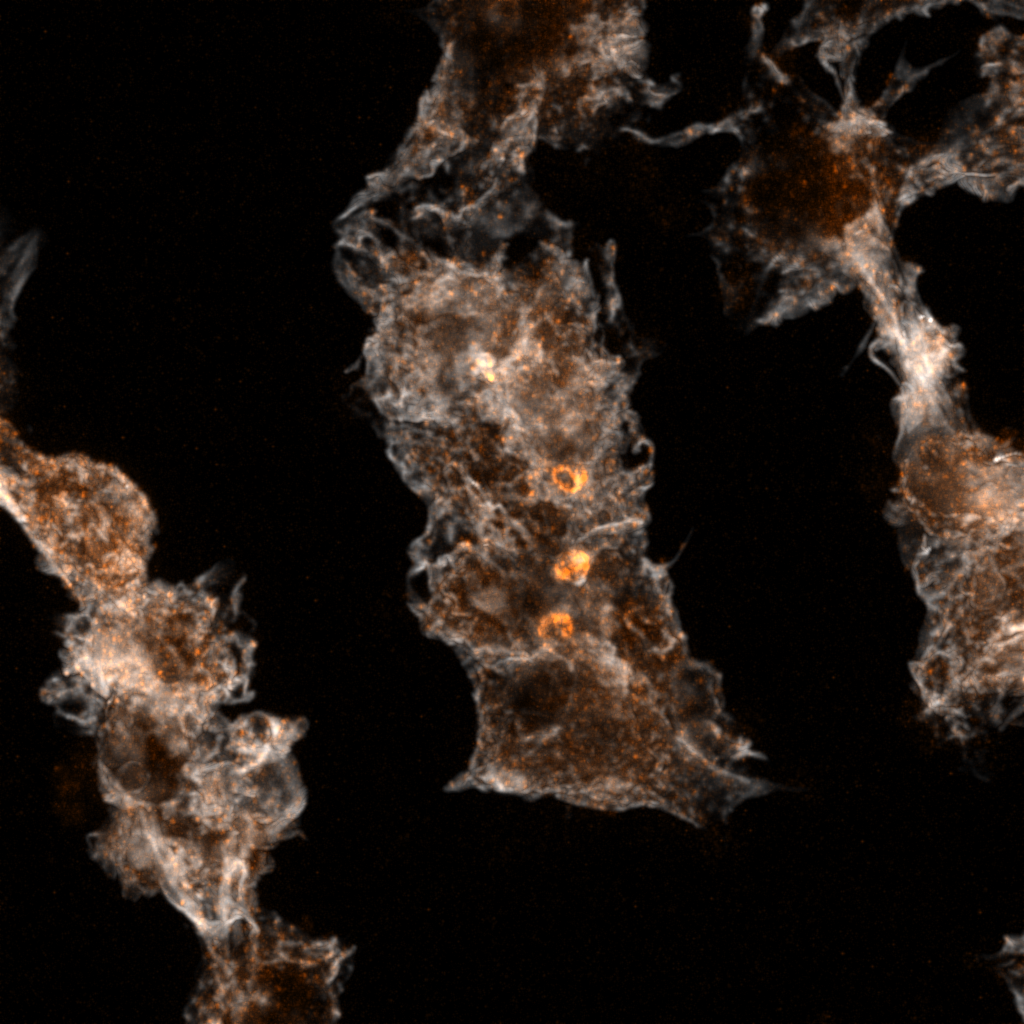

Supplement: Supplementary file 2 — Source data Fig. 1 [file 44318_2025_620_MOESM2_ESM.zip › Figure 1/1J/PSyk525 15 min C9-Deconvolv-01-Gauss-02-Create Image Subset-01_Maximum intensity projection_c2+4.tif]

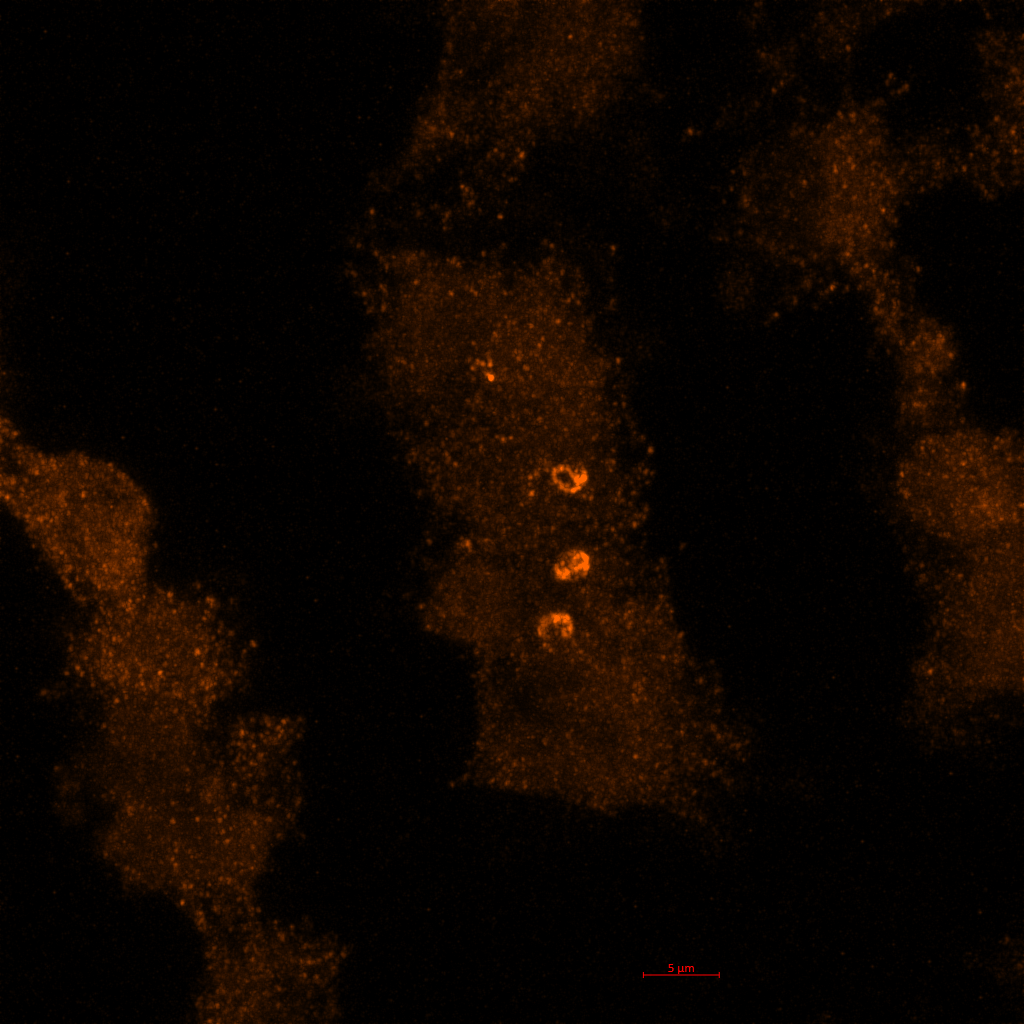

Supplement: Supplementary file 2 — Source data Fig. 1 [file 44318_2025_620_MOESM2_ESM.zip › Figure 1/1J/PSyk525 15 min C9-Measure_c2.tif]

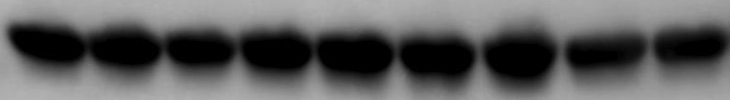

Supplement: Supplementary file 5 — Source data Fig. 4 [file 44318_2025_620_MOESM5_ESM.zip › Figure 4/4C/Western Blot Cropped TIFF Files/bactin 20230208_181205_Ch_Chemi+Marker.tiff]

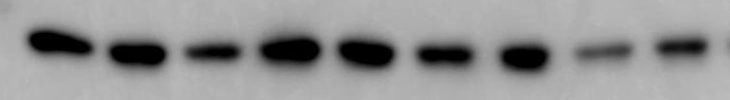

Supplement: Supplementary file 5 — Source data Fig. 4 [file 44318_2025_620_MOESM5_ESM.zip › Figure 4/4C/Western Blot Cropped TIFF Files/IkBa C term 20230208_185256_Ch_Chemi+Marker.tiff]

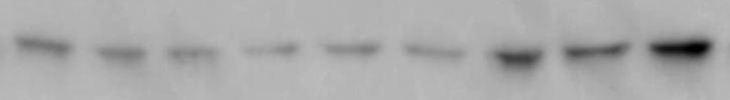

Supplement: Supplementary file 5 — Source data Fig. 4 [file 44318_2025_620_MOESM5_ESM.zip › Figure 4/4C/Western Blot Cropped TIFF Files/P-p38 20230208_184652_Ch_Chemi+Marker.tiff]

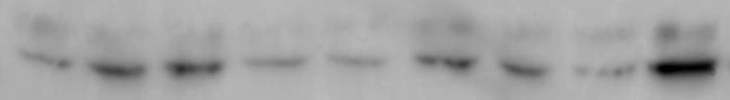

Supplement: Supplementary file 5 — Source data Fig. 4 [file 44318_2025_620_MOESM5_ESM.zip › Figure 4/4C/Western Blot Cropped TIFF Files/P-p44 42 2 20230208_184032_Ch_Chemi+Marker.tiff]

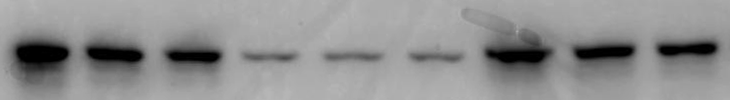

Supplement: Supplementary file 5 — Source data Fig. 4 [file 44318_2025_620_MOESM5_ESM.zip › Figure 4/4C/Western Blot Cropped TIFF Files/P-SHIP1 STC 20230208_145512_Ch_Chemi+Marker.tiff]

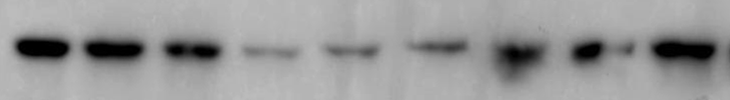

Supplement: Supplementary file 5 — Source data Fig. 4 [file 44318_2025_620_MOESM5_ESM.zip › Figure 4/4C/Western Blot Cropped TIFF Files/P-SHP-1 2 20230208_151055_Ch_Chemi+Marker.tiff]

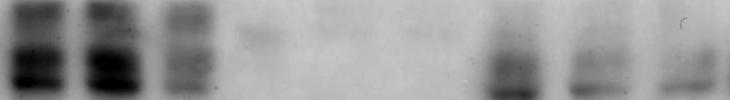

Supplement: Supplementary file 5 — Source data Fig. 4 [file 44318_2025_620_MOESM5_ESM.zip › Figure 4/4C/Western Blot Cropped TIFF Files/P-SHP-2 Y542 20230208_135035_Ch_Chemi+Marker.tiff]

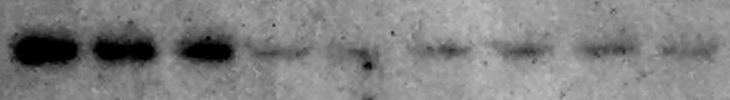

Supplement: Supplementary file 5 — Source data Fig. 4 [file 44318_2025_620_MOESM5_ESM.zip › Figure 4/4C/Western Blot Cropped TIFF Files/P-SHP-2 Y580 20230208_132431_Ch_Chemi+Marker.tiff]

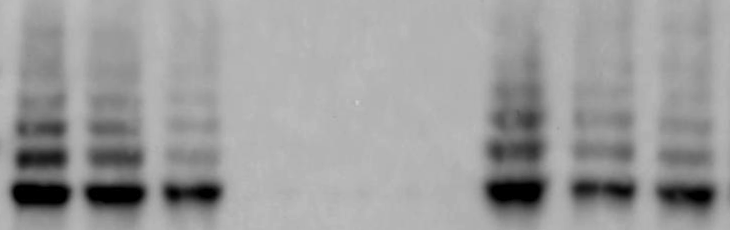

Supplement: Supplementary file 5 — Source data Fig. 4 [file 44318_2025_620_MOESM5_ESM.zip › Figure 4/4C/Western Blot Cropped TIFF Files/P-Syk Y346 2 20230208_145025_Ch_Chemi+Marker.tiff]

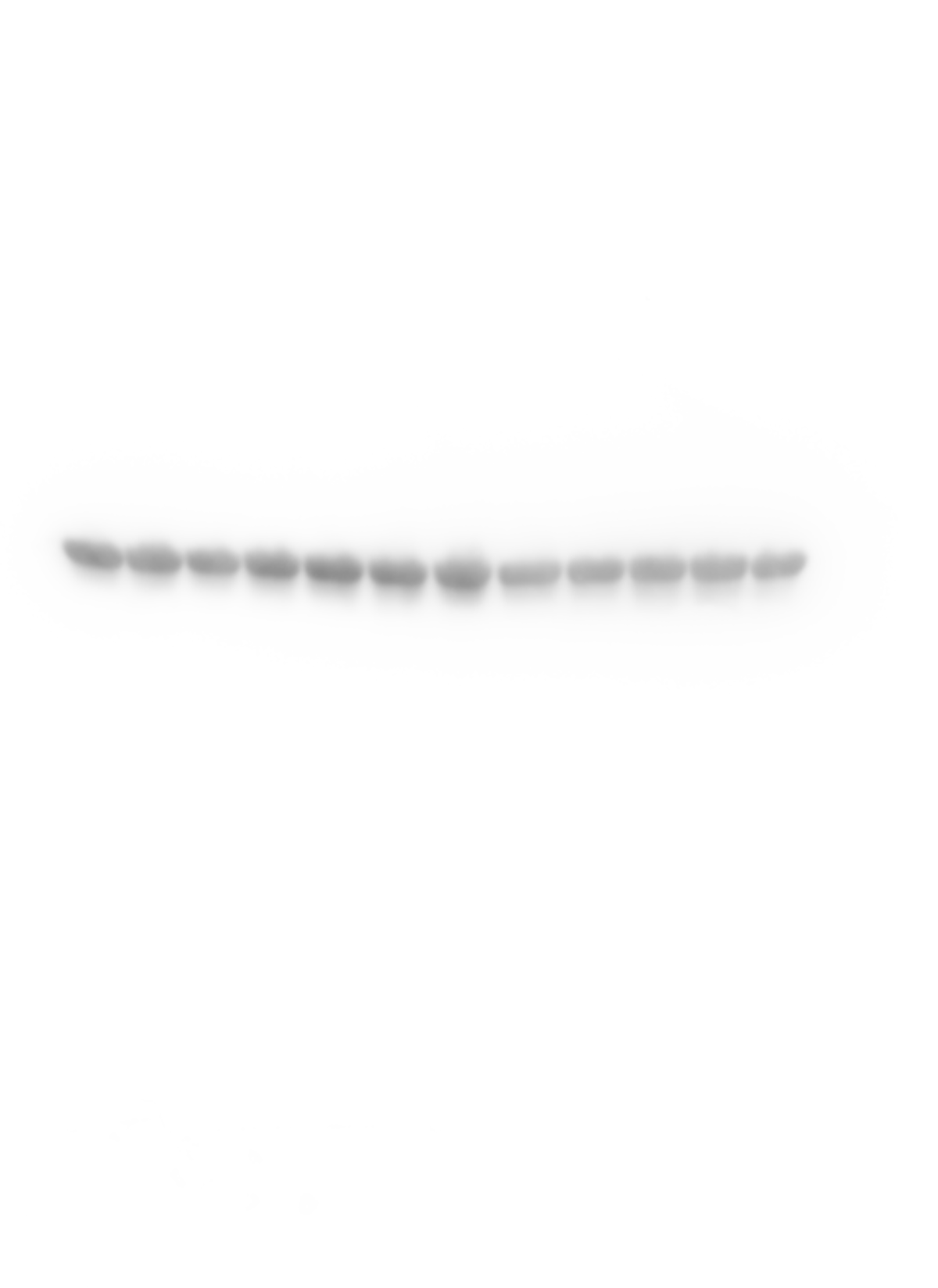

Supplement: Supplementary file 5 — Source data Fig. 4 [file 44318_2025_620_MOESM5_ESM.zip › Figure 4/4C/Western Blot Image Files/bactin 20230208_181205_Ch/bactin 20230208_181205_Ch_Chemi.tif]

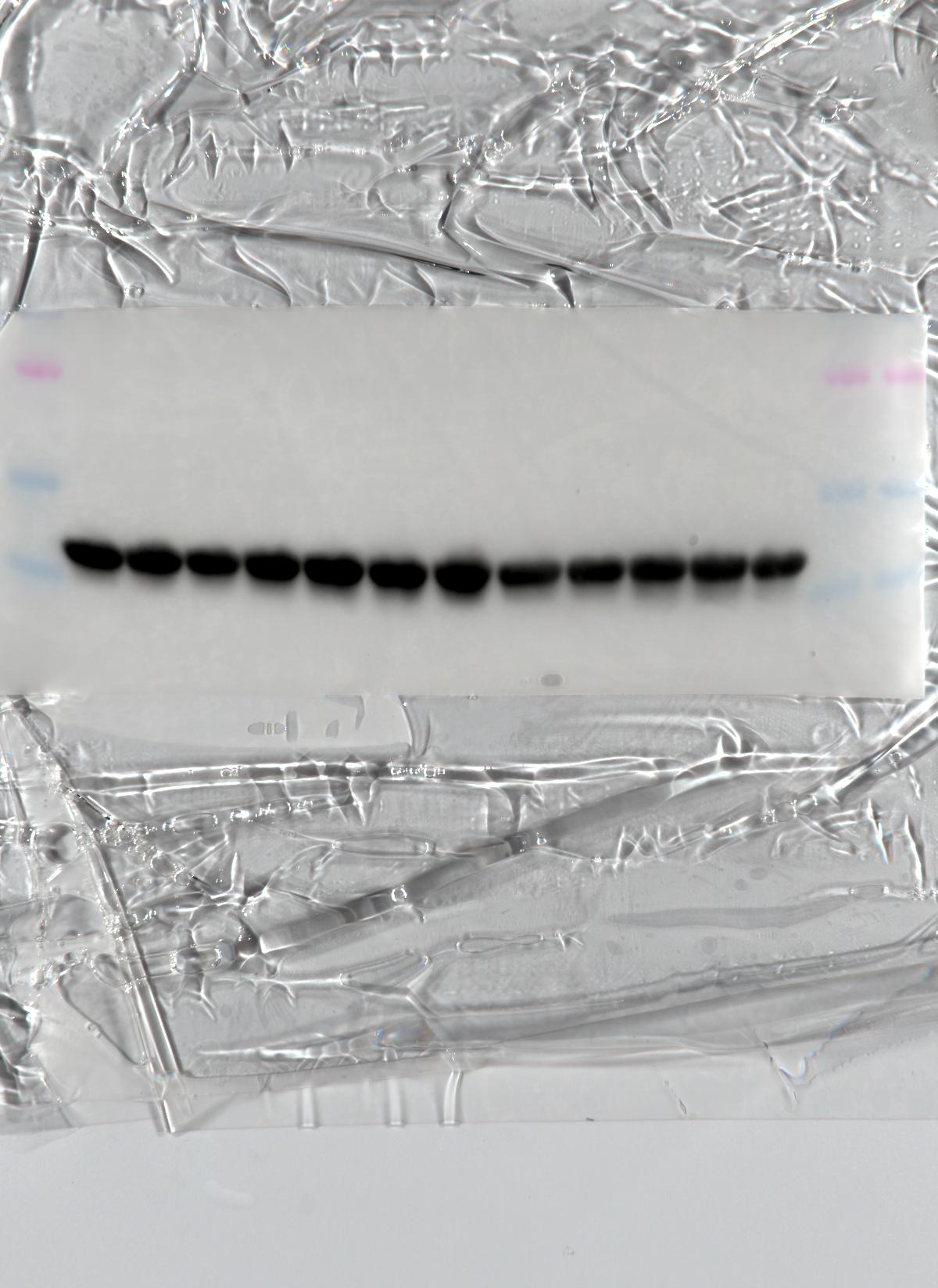

Supplement: Supplementary file 5 — Source data Fig. 4 [file 44318_2025_620_MOESM5_ESM.zip › Figure 4/4C/Western Blot Image Files/bactin 20230208_181205_Ch/bactin 20230208_181205_Ch_Chemi+Marker.jpg]

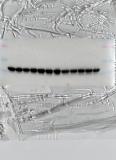

Supplement: Supplementary file 5 — Source data Fig. 4 [file 44318_2025_620_MOESM5_ESM.zip › Figure 4/4C/Western Blot Image Files/bactin 20230208_181205_Ch/bactin 20230208_181205_Ch_Thumb.jpg]

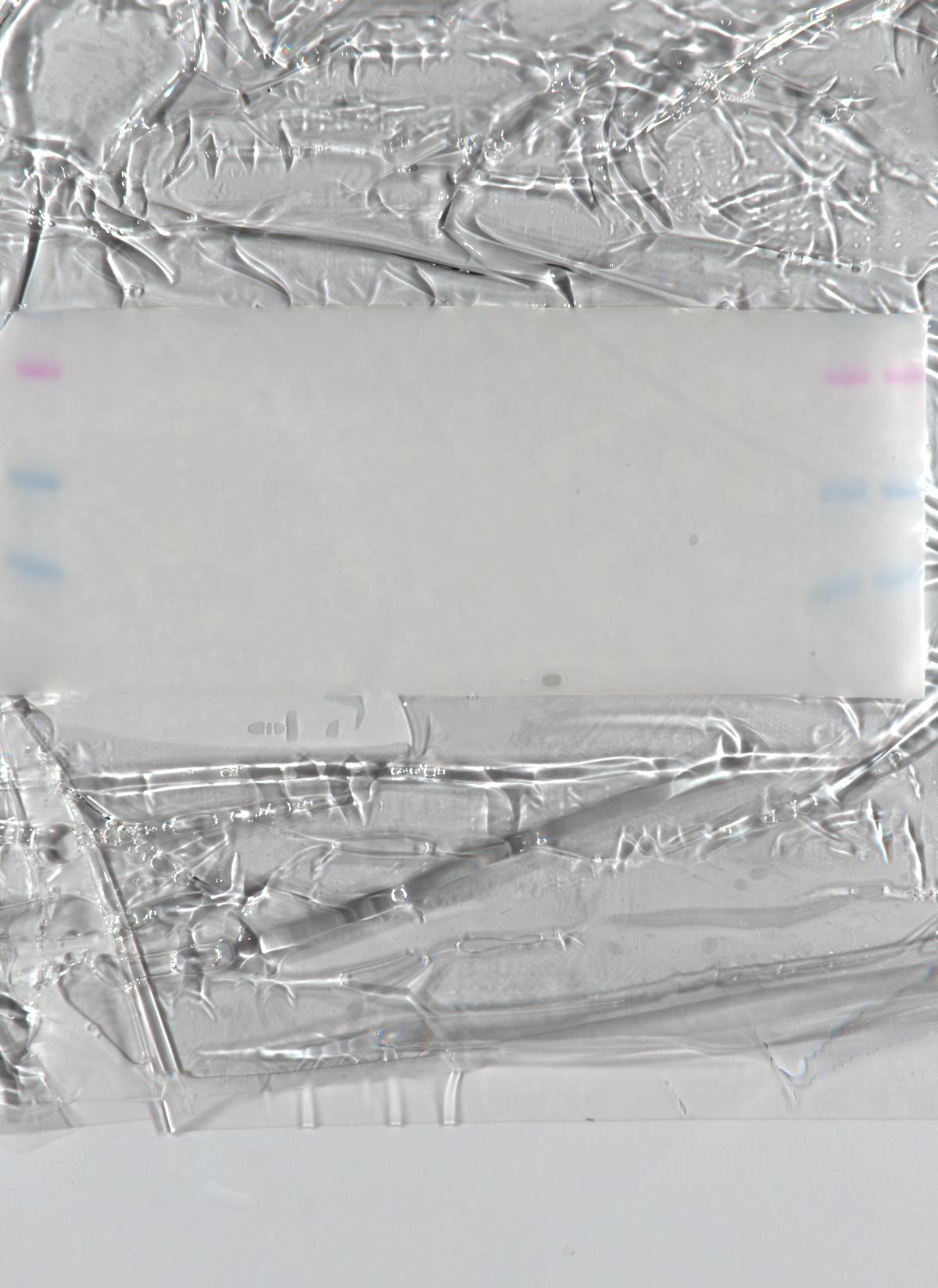

Supplement: Supplementary file 5 — Source data Fig. 4 [file 44318_2025_620_MOESM5_ESM.zip › Figure 4/4C/Western Blot Image Files/bactin 20230208_181205_Ch/bactin 20230208_181205_Ch-Marker.jpg]

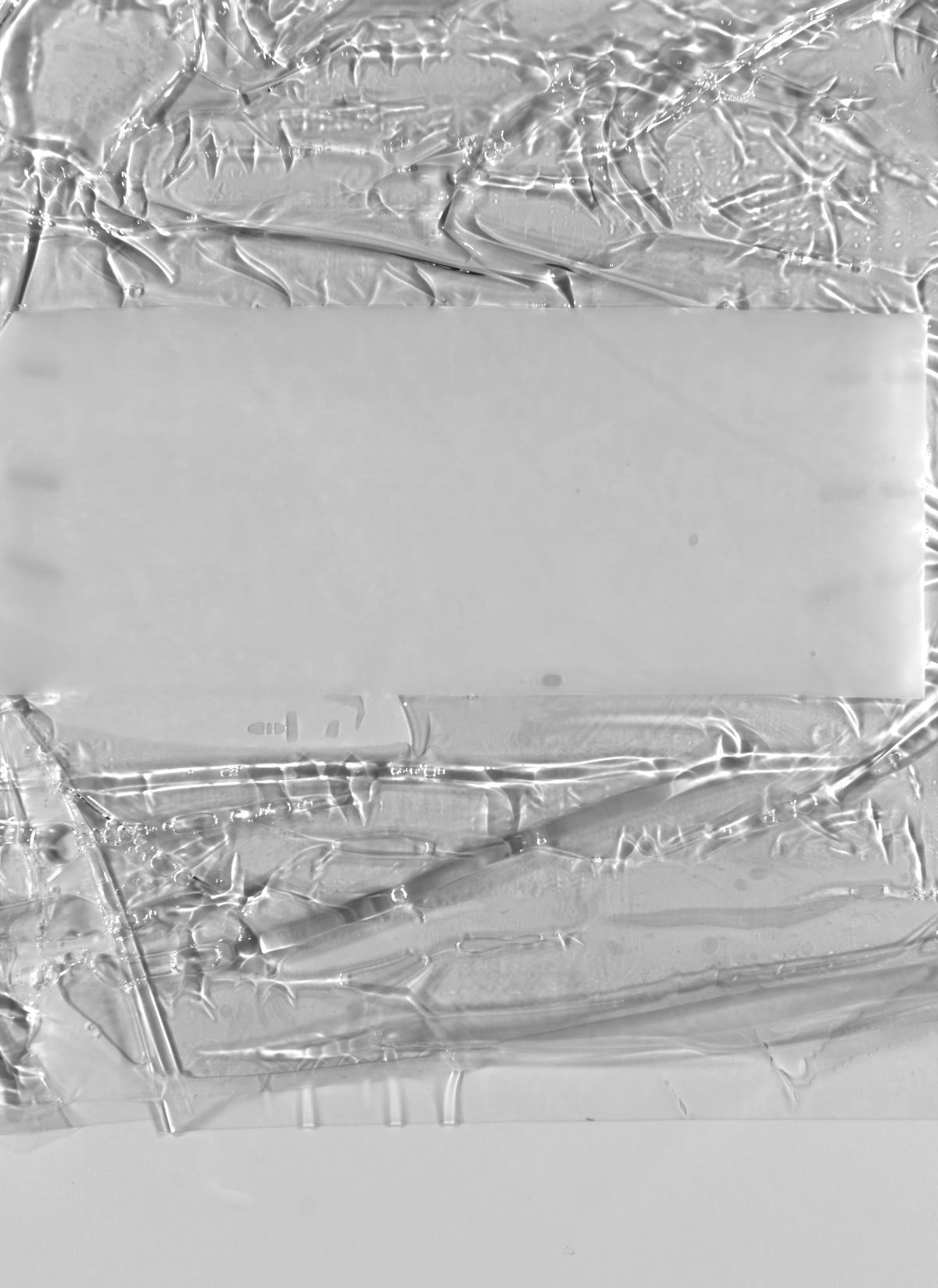

Supplement: Supplementary file 5 — Source data Fig. 4 [file 44318_2025_620_MOESM5_ESM.zip › Figure 4/4C/Western Blot Image Files/bactin 20230208_181205_Ch/bactin 20230208_181205_Ch-Marker.tif]

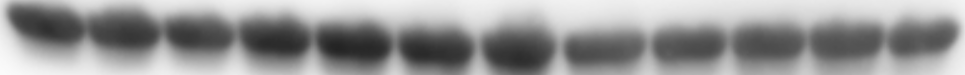

Supplement: Supplementary file 5 — Source data Fig. 4 [file 44318_2025_620_MOESM5_ESM.zip › Figure 4/4C/Western Blot Image Files/bactin 20230208_181205_Ch/bactin.tif]

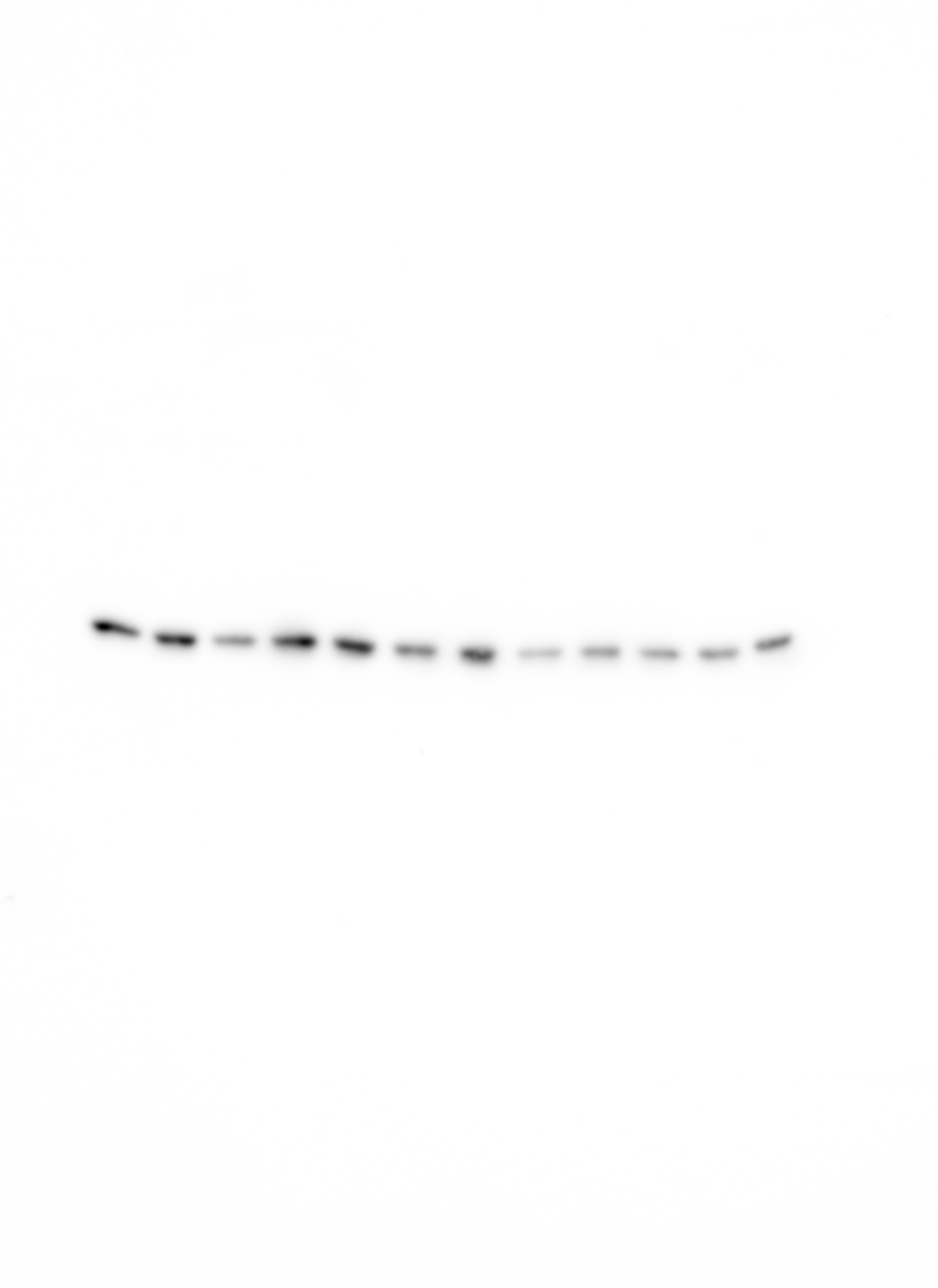

Supplement: Supplementary file 5 — Source data Fig. 4 [file 44318_2025_620_MOESM5_ESM.zip › Figure 4/4C/Western Blot Image Files/IkBa C term 20230208_185256_Ch/IkBa C term 20230208_185256_Ch_Chemi.tif]

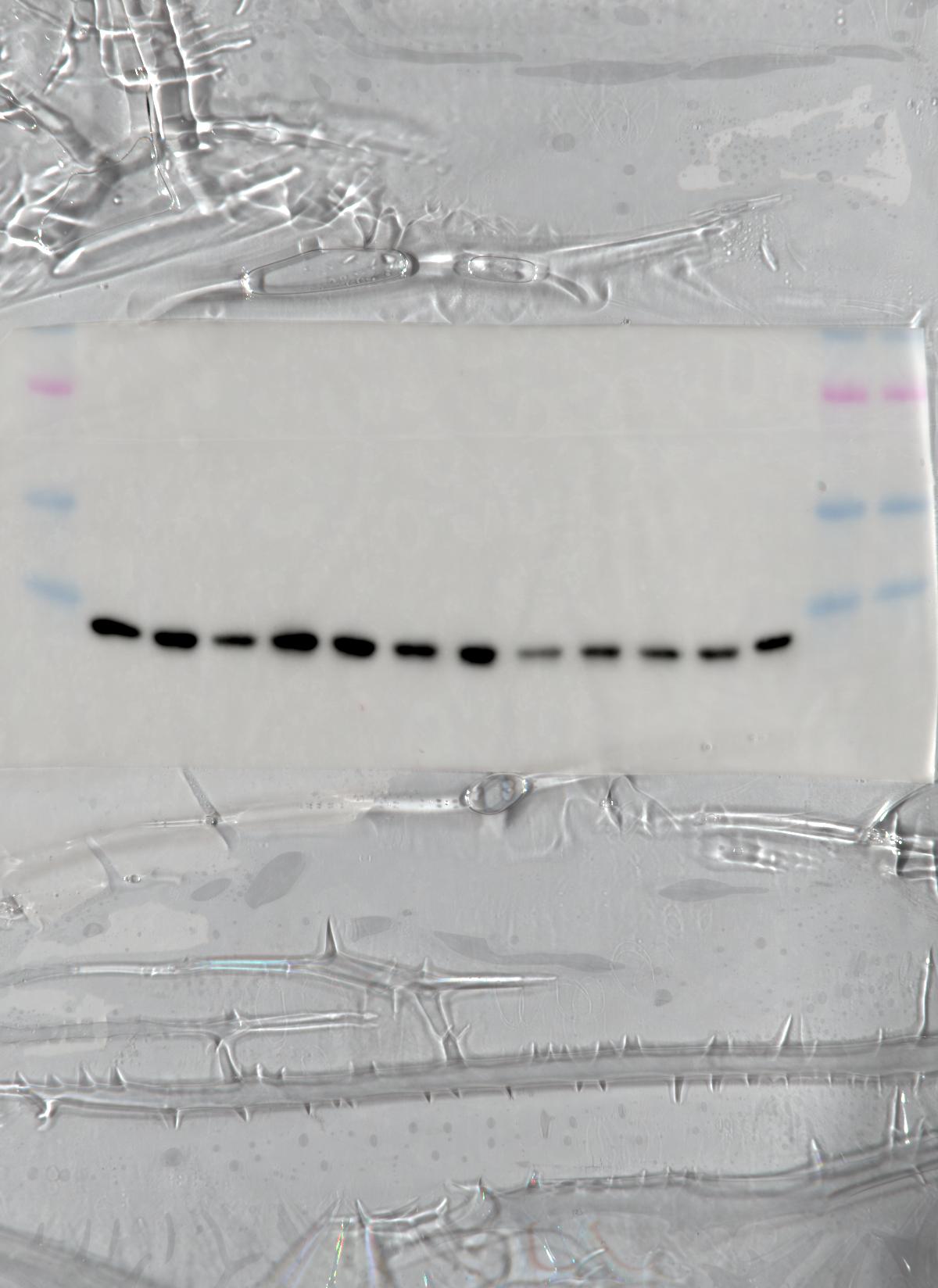

Supplement: Supplementary file 5 — Source data Fig. 4 [file 44318_2025_620_MOESM5_ESM.zip › Figure 4/4C/Western Blot Image Files/IkBa C term 20230208_185256_Ch/IkBa C term 20230208_185256_Ch_Chemi+Marker.jpg]

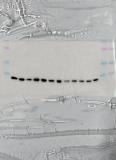

Supplement: Supplementary file 5 — Source data Fig. 4 [file 44318_2025_620_MOESM5_ESM.zip › Figure 4/4C/Western Blot Image Files/IkBa C term 20230208_185256_Ch/IkBa C term 20230208_185256_Ch_Thumb.jpg]

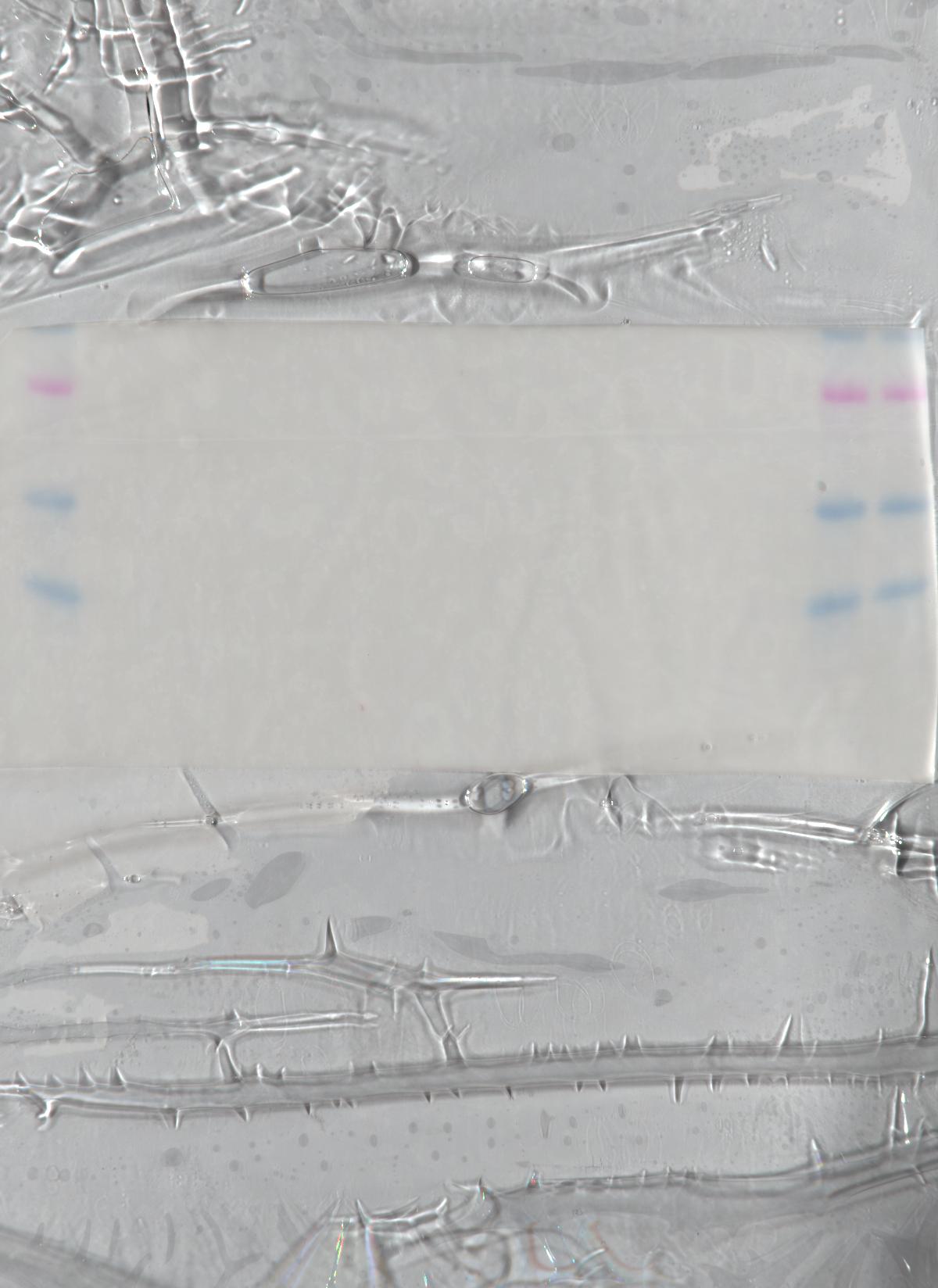

Supplement: Supplementary file 5 — Source data Fig. 4 [file 44318_2025_620_MOESM5_ESM.zip › Figure 4/4C/Western Blot Image Files/IkBa C term 20230208_185256_Ch/IkBa C term 20230208_185256_Ch-Marker.jpg]

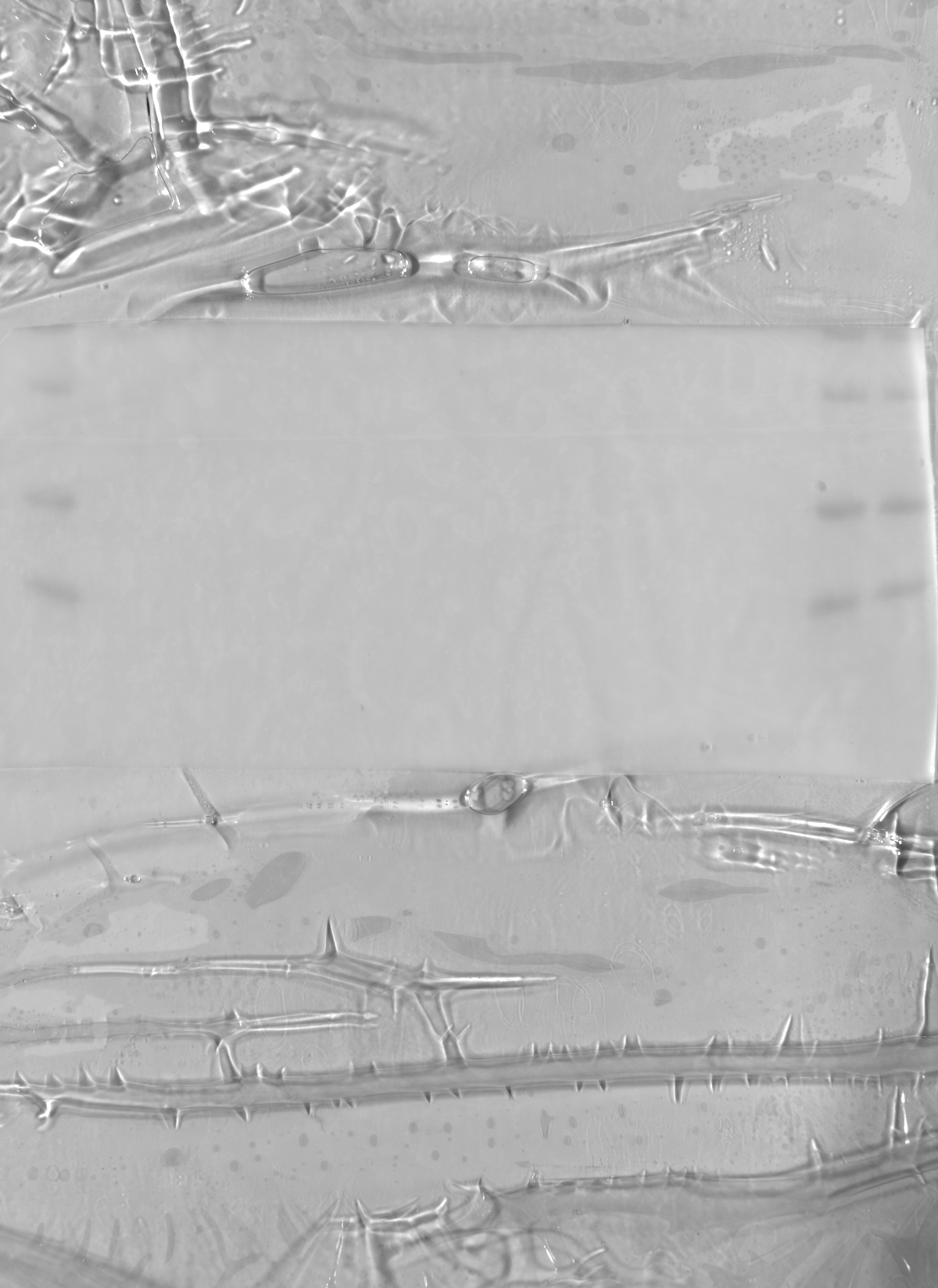

Supplement: Supplementary file 5 — Source data Fig. 4 [file 44318_2025_620_MOESM5_ESM.zip › Figure 4/4C/Western Blot Image Files/IkBa C term 20230208_185256_Ch/IkBa C term 20230208_185256_Ch-Marker.tif]

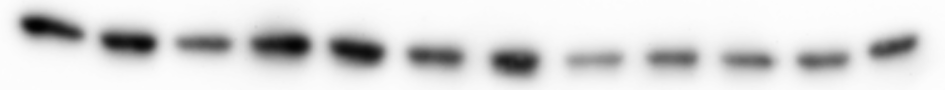

Supplement: Supplementary file 5 — Source data Fig. 4 [file 44318_2025_620_MOESM5_ESM.zip › Figure 4/4C/Western Blot Image Files/IkBa C term 20230208_185256_Ch/IkBa C term.tif]

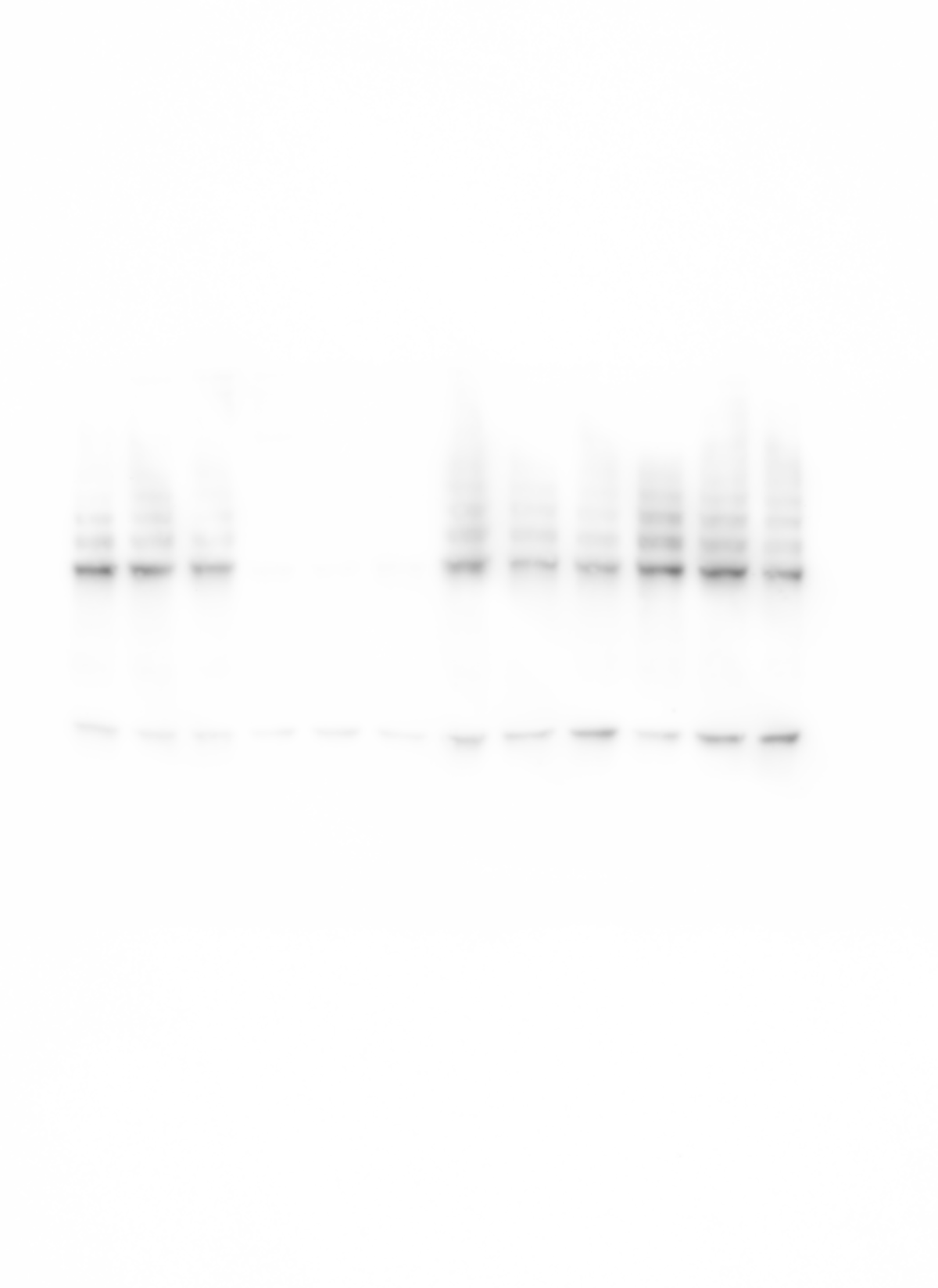

Supplement: Supplementary file 5 — Source data Fig. 4 [file 44318_2025_620_MOESM5_ESM.zip › Figure 4/4C/Western Blot Image Files/P-p38 20230208_184652_Ch/P-p38 20230208_184652_Ch_Chemi.tif]

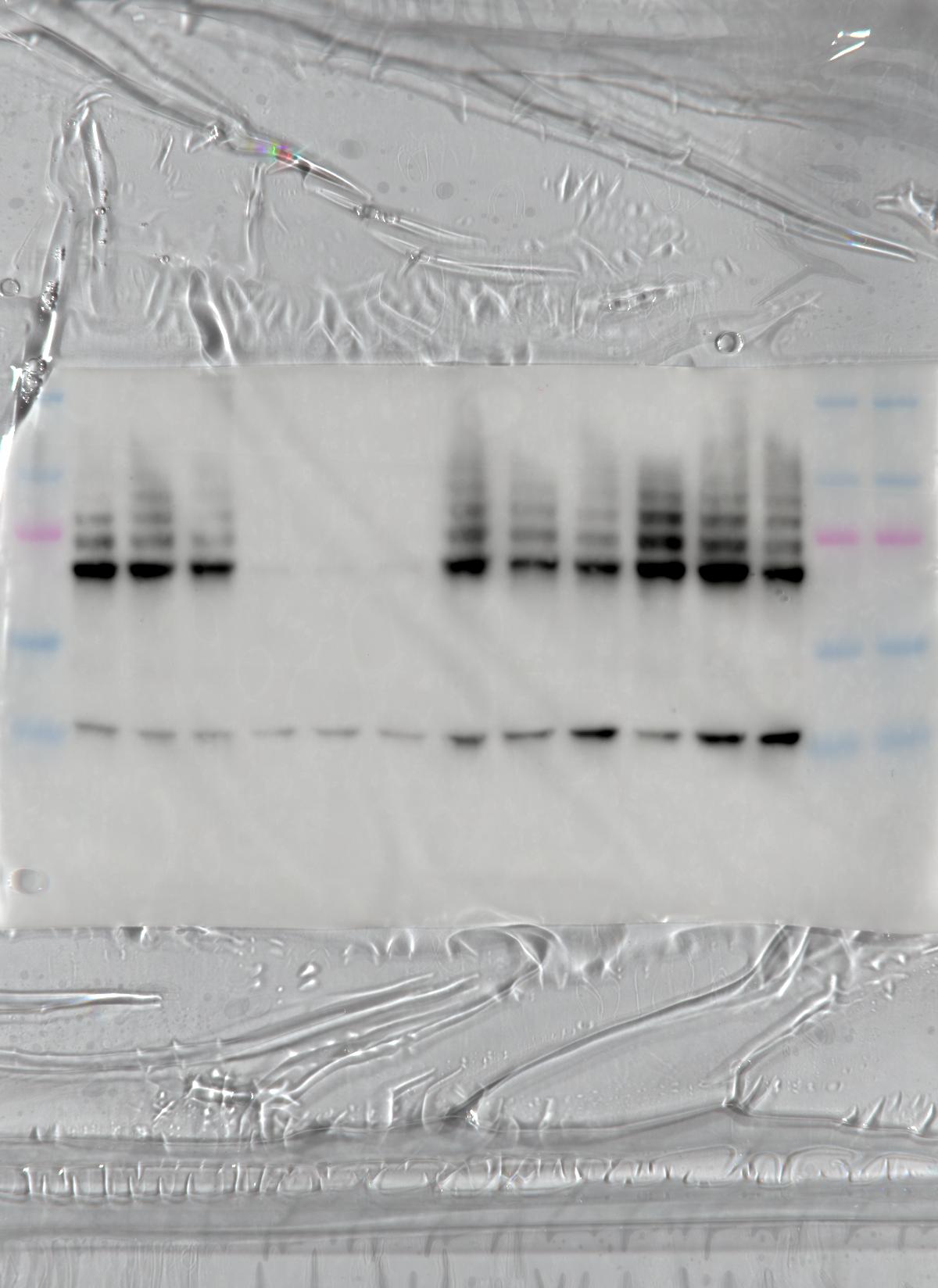

Supplement: Supplementary file 5 — Source data Fig. 4 [file 44318_2025_620_MOESM5_ESM.zip › Figure 4/4C/Western Blot Image Files/P-p38 20230208_184652_Ch/P-p38 20230208_184652_Ch_Chemi+Marker.jpg]

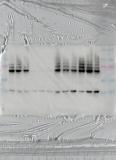

Supplement: Supplementary file 5 — Source data Fig. 4 [file 44318_2025_620_MOESM5_ESM.zip › Figure 4/4C/Western Blot Image Files/P-p38 20230208_184652_Ch/P-p38 20230208_184652_Ch_Thumb.jpg]

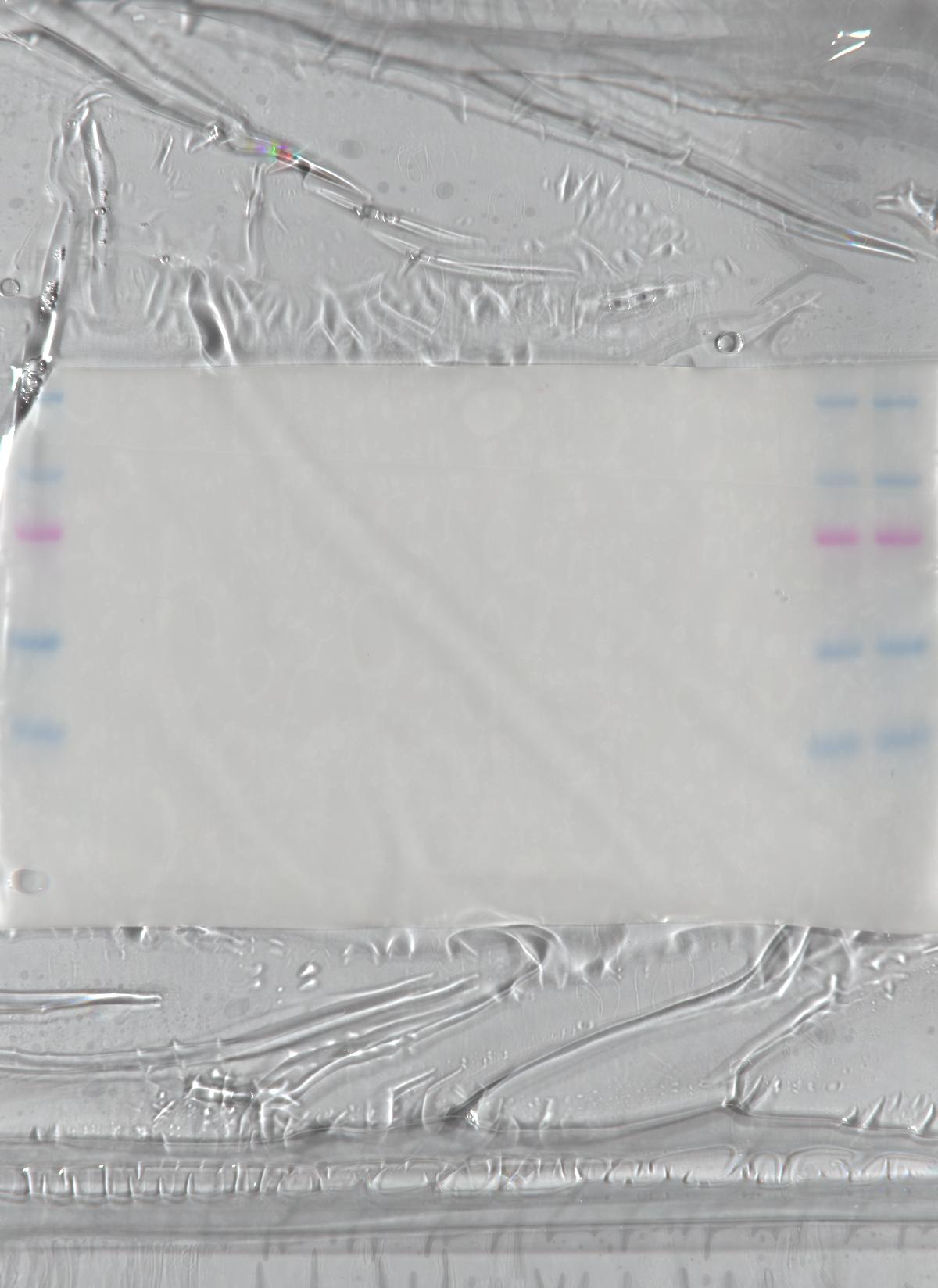

Supplement: Supplementary file 5 — Source data Fig. 4 [file 44318_2025_620_MOESM5_ESM.zip › Figure 4/4C/Western Blot Image Files/P-p38 20230208_184652_Ch/P-p38 20230208_184652_Ch-Marker.jpg]

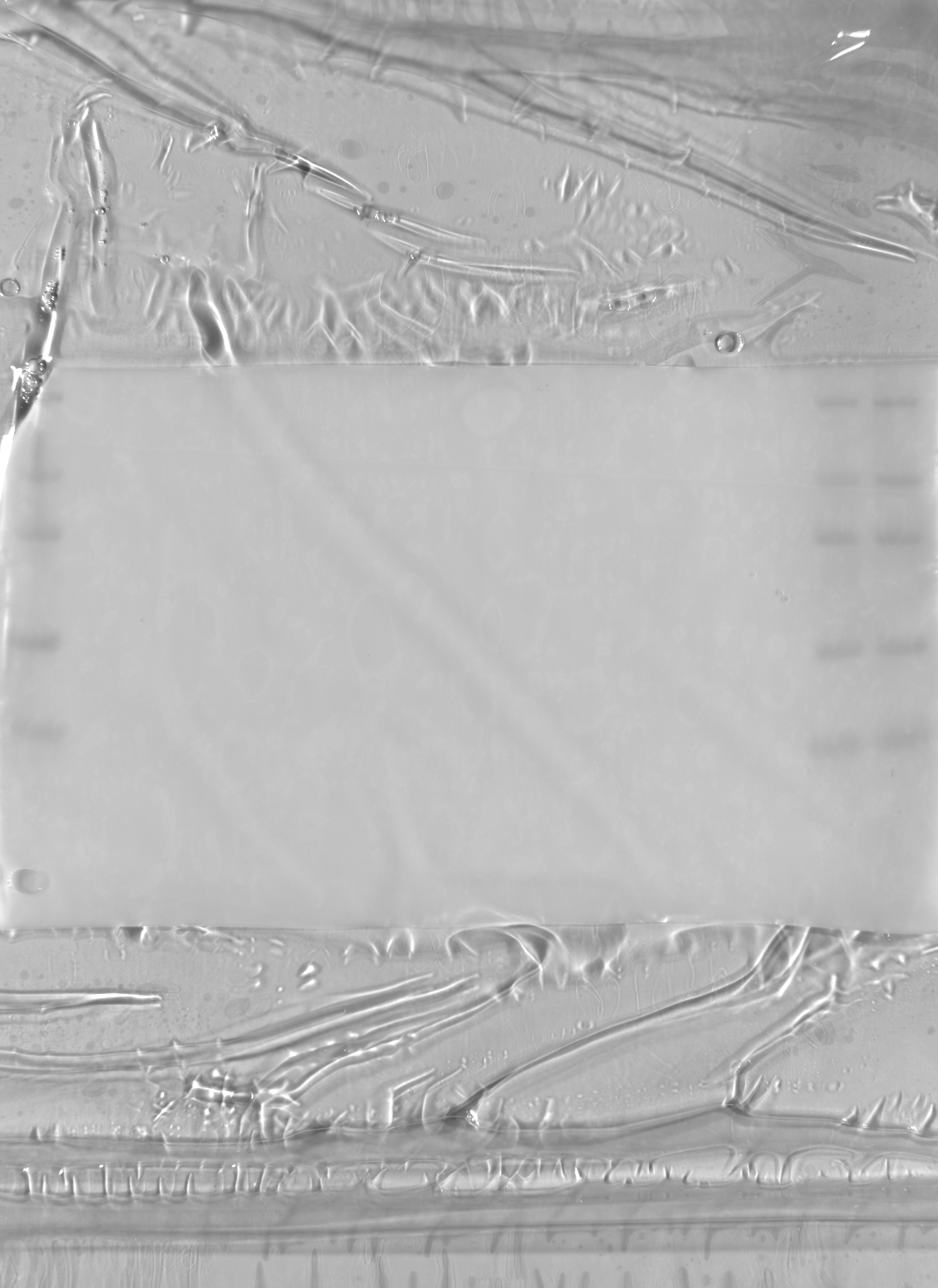

Supplement: Supplementary file 5 — Source data Fig. 4 [file 44318_2025_620_MOESM5_ESM.zip › Figure 4/4C/Western Blot Image Files/P-p38 20230208_184652_Ch/P-p38 20230208_184652_Ch-Marker.tif]

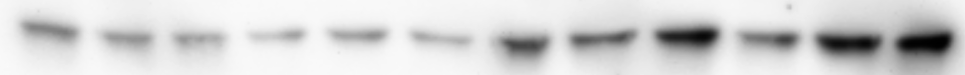

Supplement: Supplementary file 5 — Source data Fig. 4 [file 44318_2025_620_MOESM5_ESM.zip › Figure 4/4C/Western Blot Image Files/P-p38 20230208_184652_Ch/P-p38.tif]

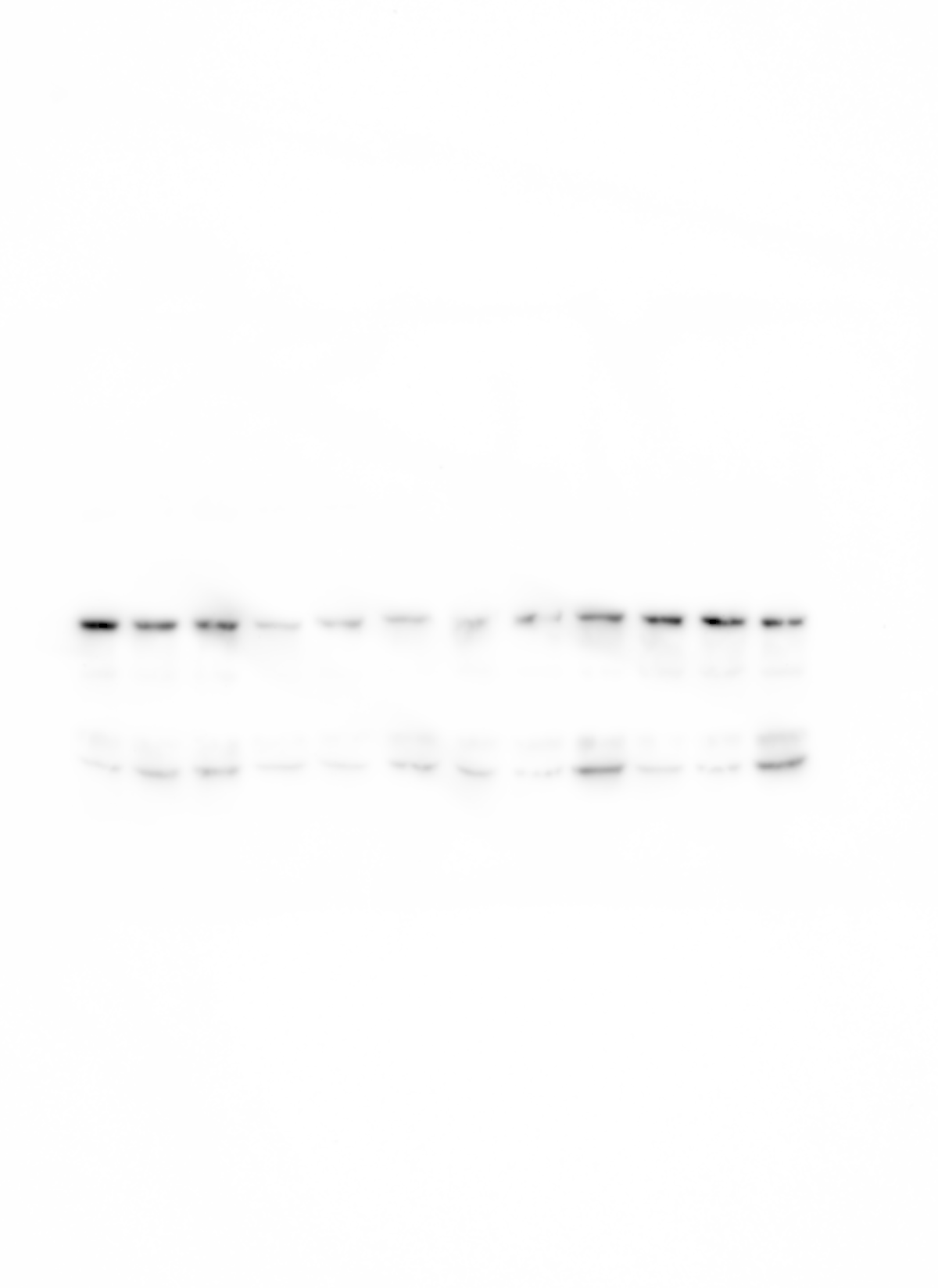

Supplement: Supplementary file 5 — Source data Fig. 4 [file 44318_2025_620_MOESM5_ESM.zip › Figure 4/4C/Western Blot Image Files/P-p44 42 2 20230208_184032_Ch/P-p44 42 2 20230208_184032_Ch_Chemi.tif]

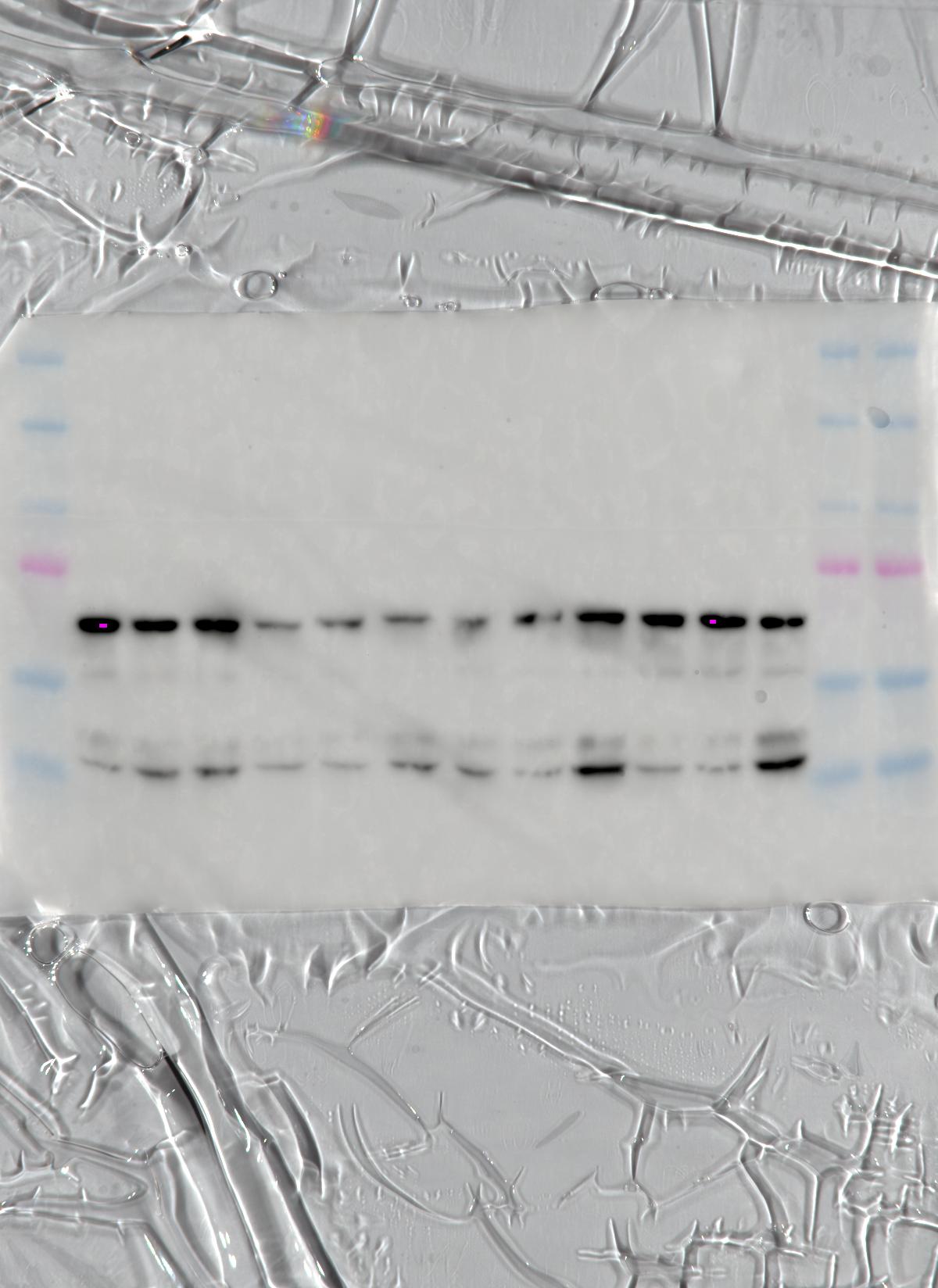

Supplement: Supplementary file 5 — Source data Fig. 4 [file 44318_2025_620_MOESM5_ESM.zip › Figure 4/4C/Western Blot Image Files/P-p44 42 2 20230208_184032_Ch/P-p44 42 2 20230208_184032_Ch_Chemi+Marker.jpg]

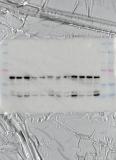

Supplement: Supplementary file 5 — Source data Fig. 4 [file 44318_2025_620_MOESM5_ESM.zip › Figure 4/4C/Western Blot Image Files/P-p44 42 2 20230208_184032_Ch/P-p44 42 2 20230208_184032_Ch_Thumb.jpg]

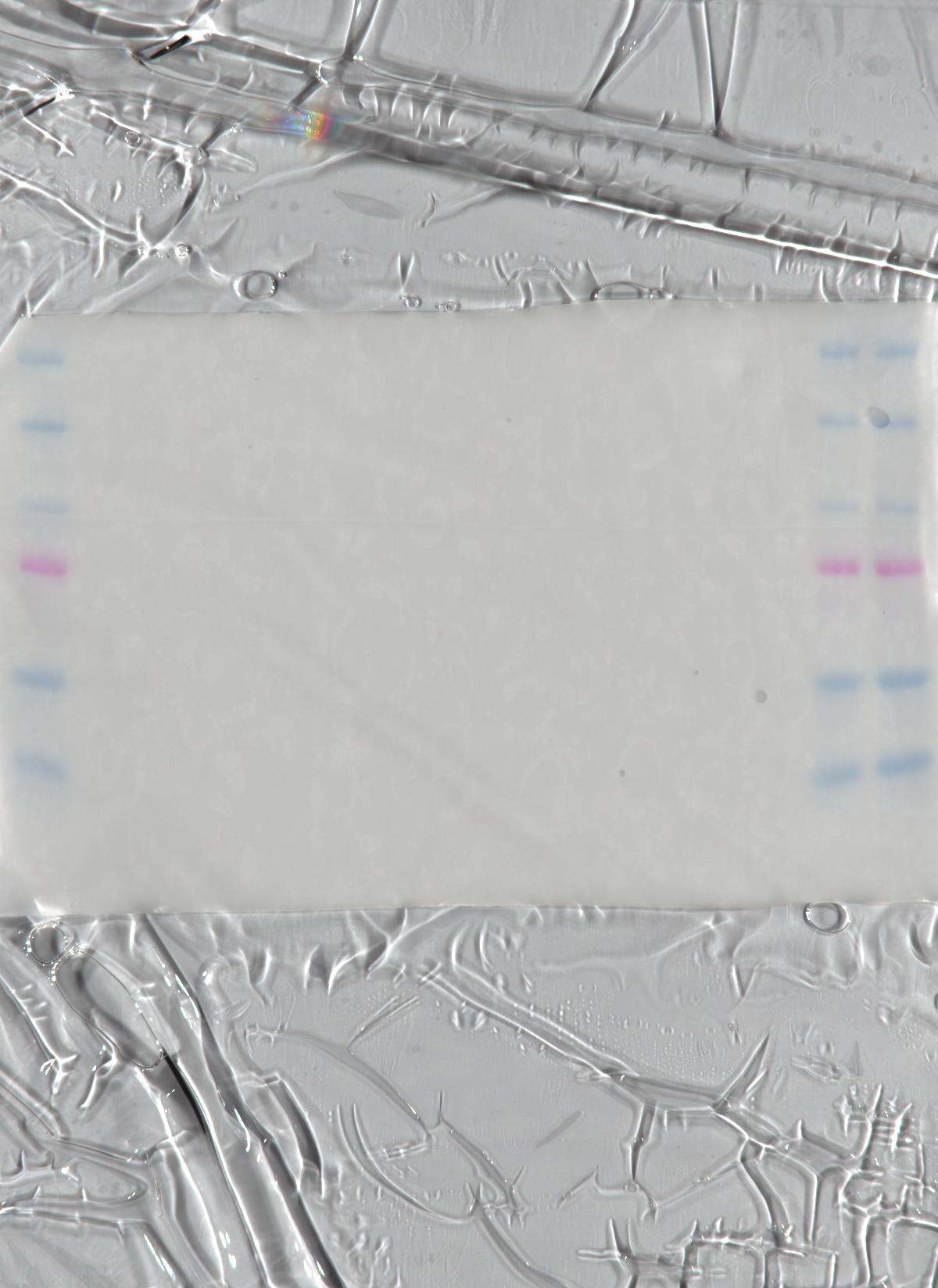

Supplement: Supplementary file 5 — Source data Fig. 4 [file 44318_2025_620_MOESM5_ESM.zip › Figure 4/4C/Western Blot Image Files/P-p44 42 2 20230208_184032_Ch/P-p44 42 2 20230208_184032_Ch-Marker.jpg]

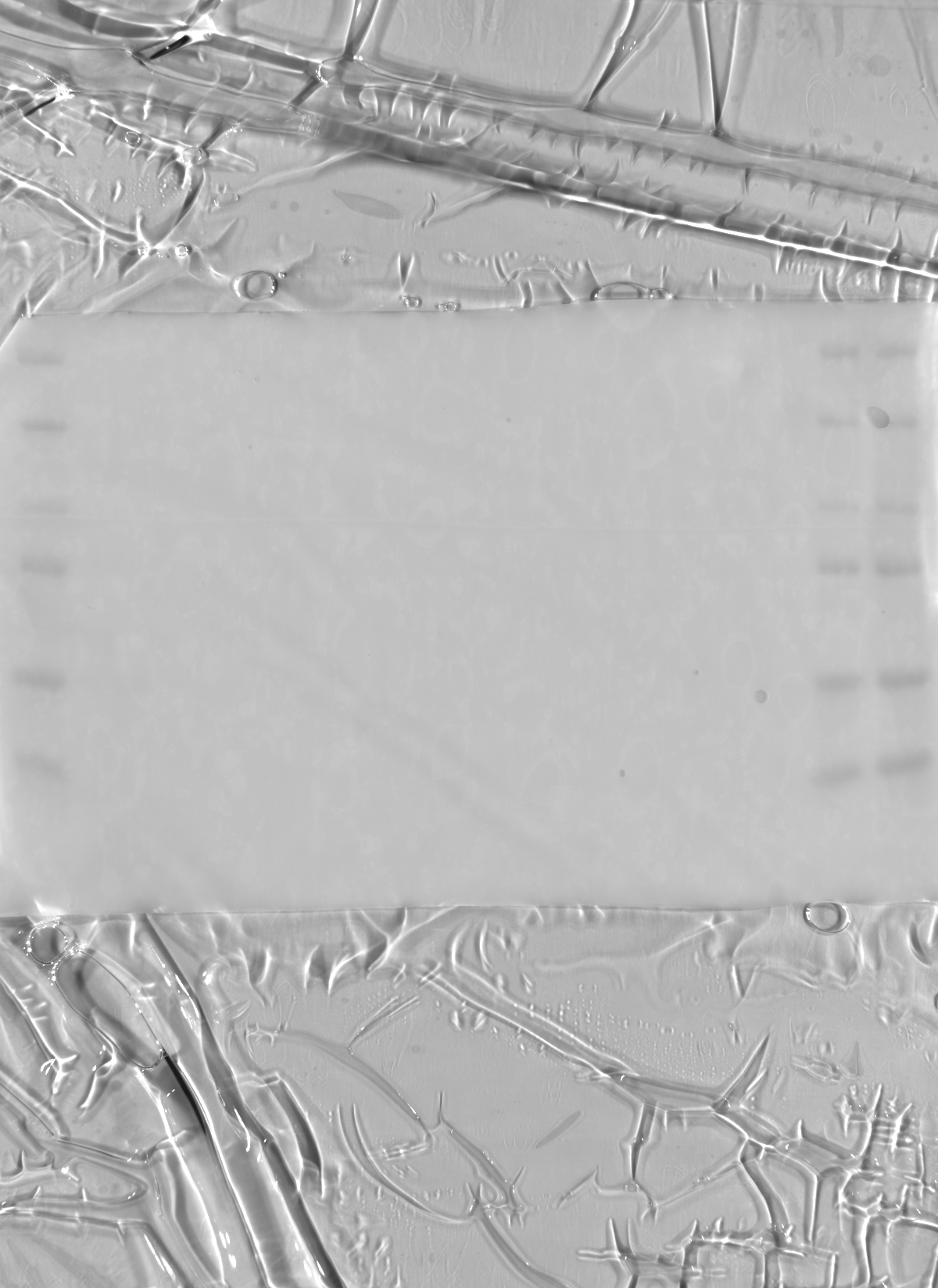

Supplement: Supplementary file 5 — Source data Fig. 4 [file 44318_2025_620_MOESM5_ESM.zip › Figure 4/4C/Western Blot Image Files/P-p44 42 2 20230208_184032_Ch/P-p44 42 2 20230208_184032_Ch-Marker.tif]

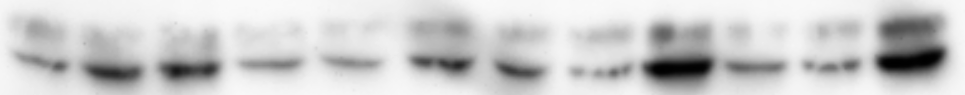

Supplement: Supplementary file 5 — Source data Fig. 4 [file 44318_2025_620_MOESM5_ESM.zip › Figure 4/4C/Western Blot Image Files/P-p44 42 2 20230208_184032_Ch/P-p44 42 2.tif]

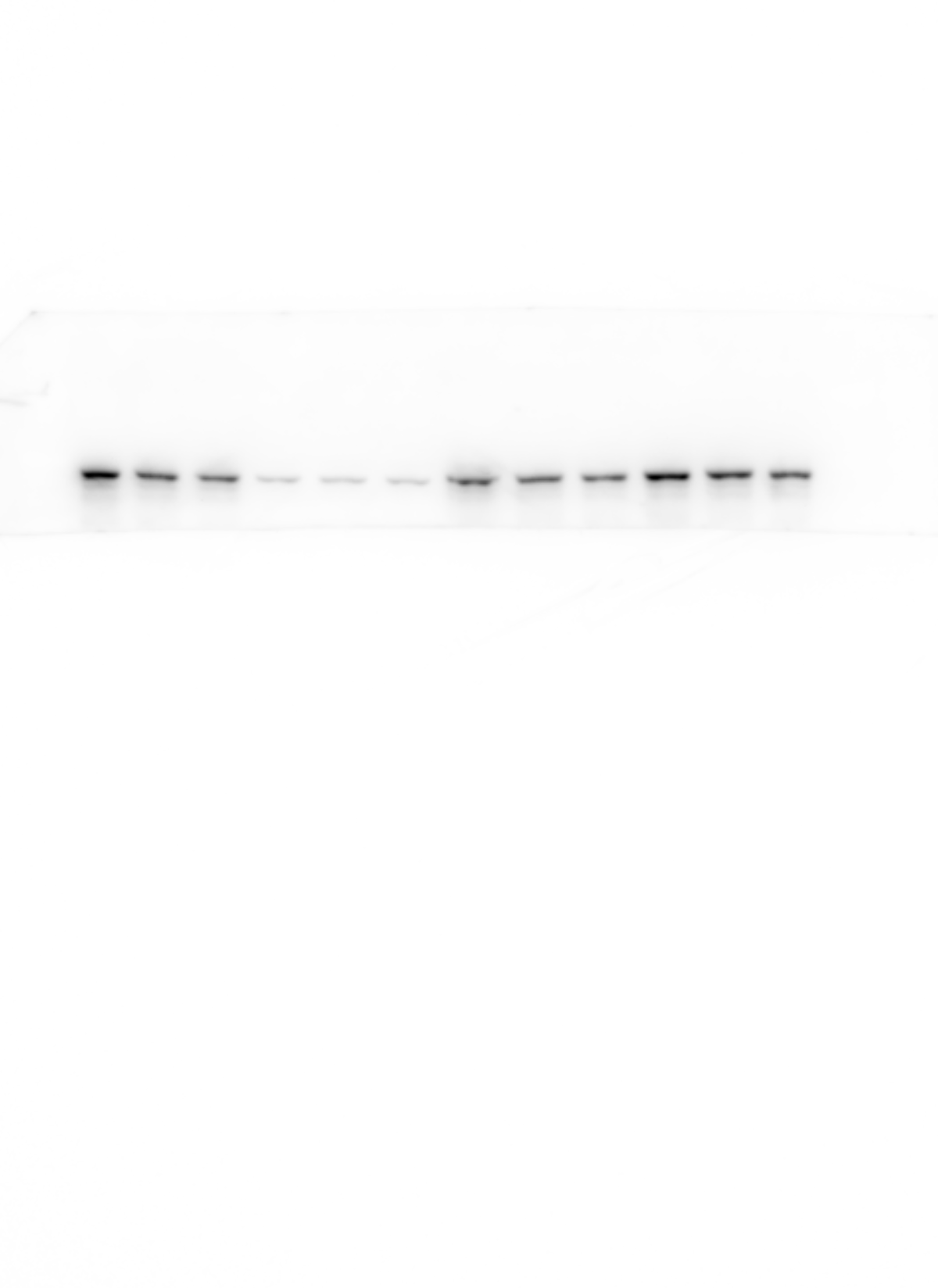

Supplement: Supplementary file 5 — Source data Fig. 4 [file 44318_2025_620_MOESM5_ESM.zip › Figure 4/4C/Western Blot Image Files/P-SHIP1 STC 20230208_145512_Ch/P-SHIP1 STC 20230208_145512_Ch_Chemi.tif]

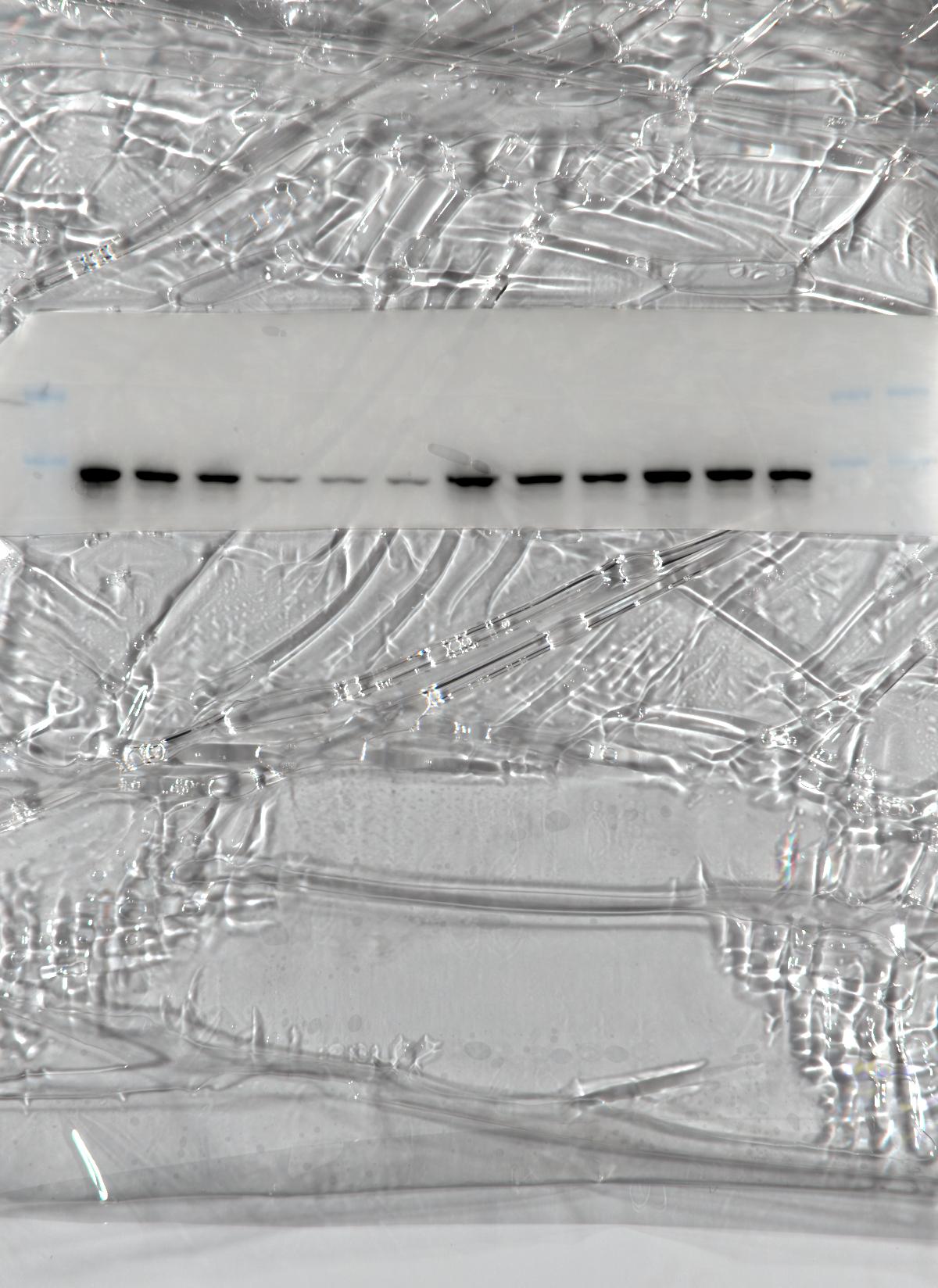

Supplement: Supplementary file 5 — Source data Fig. 4 [file 44318_2025_620_MOESM5_ESM.zip › Figure 4/4C/Western Blot Image Files/P-SHIP1 STC 20230208_145512_Ch/P-SHIP1 STC 20230208_145512_Ch_Chemi+Marker.jpg]

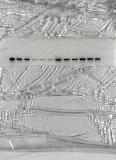

Supplement: Supplementary file 5 — Source data Fig. 4 [file 44318_2025_620_MOESM5_ESM.zip › Figure 4/4C/Western Blot Image Files/P-SHIP1 STC 20230208_145512_Ch/P-SHIP1 STC 20230208_145512_Ch_Thumb.jpg]

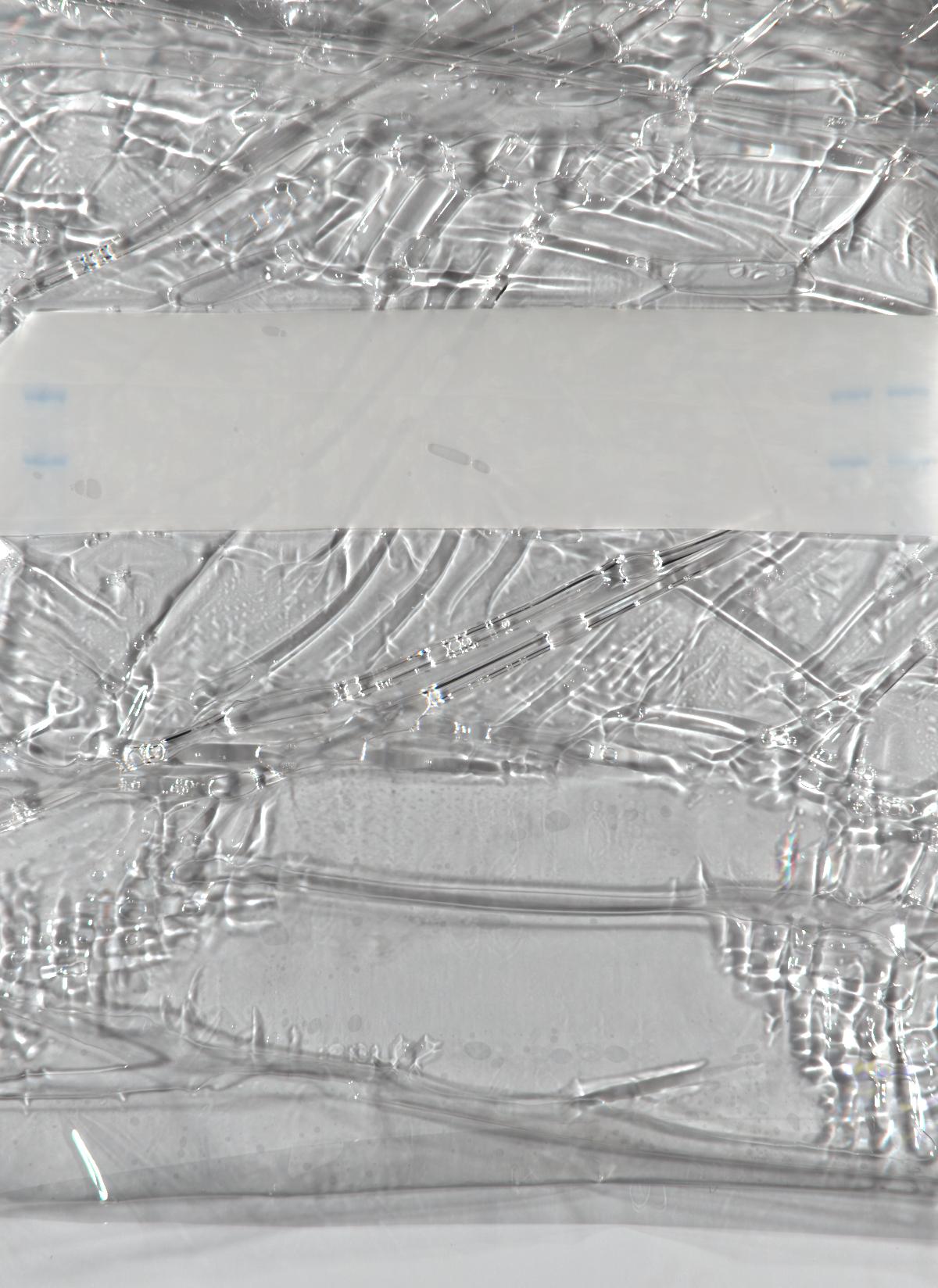

Supplement: Supplementary file 5 — Source data Fig. 4 [file 44318_2025_620_MOESM5_ESM.zip › Figure 4/4C/Western Blot Image Files/P-SHIP1 STC 20230208_145512_Ch/P-SHIP1 STC 20230208_145512_Ch-Marker.jpg]

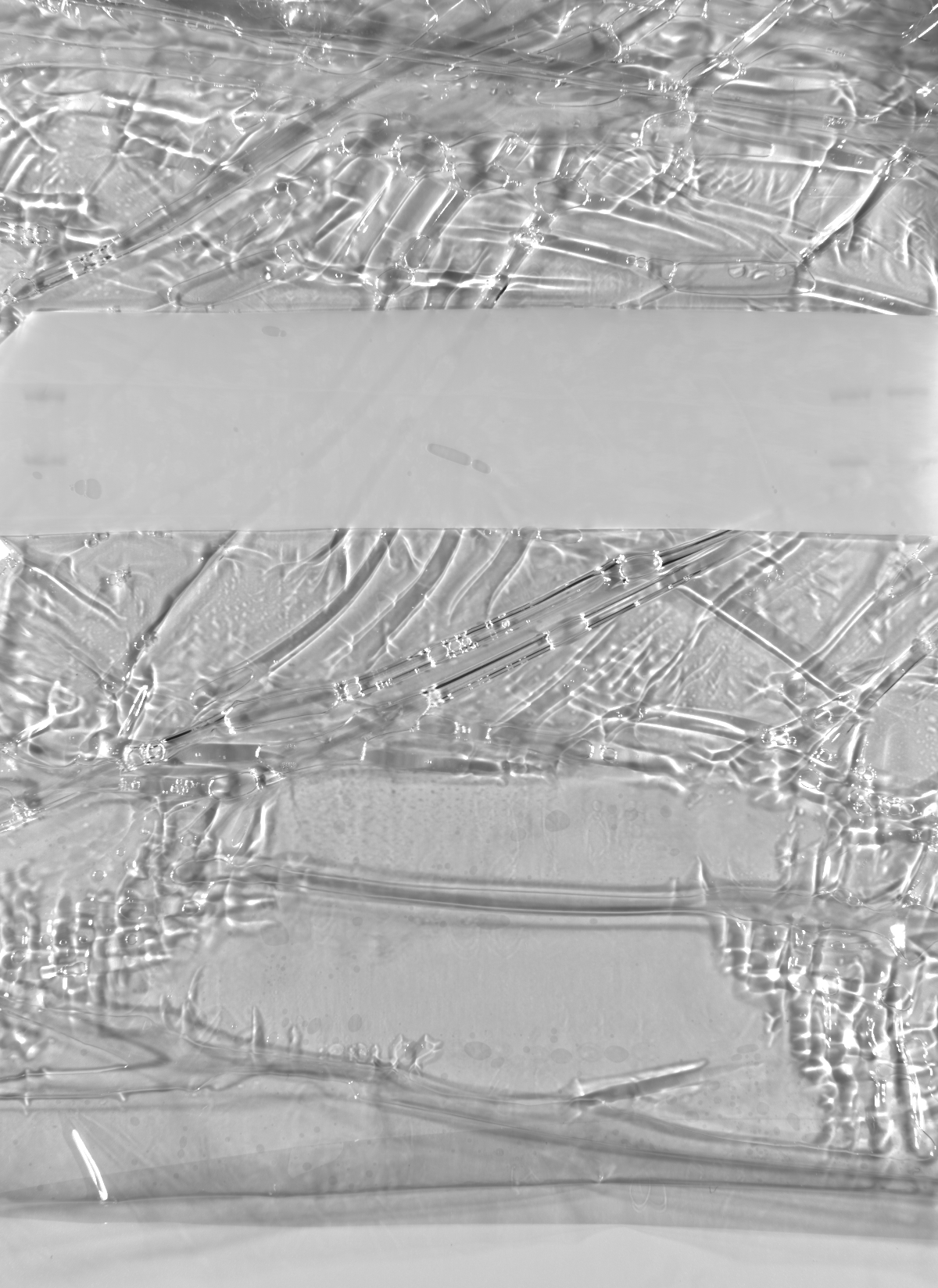

Supplement: Supplementary file 5 — Source data Fig. 4 [file 44318_2025_620_MOESM5_ESM.zip › Figure 4/4C/Western Blot Image Files/P-SHIP1 STC 20230208_145512_Ch/P-SHIP1 STC 20230208_145512_Ch-Marker.tif]

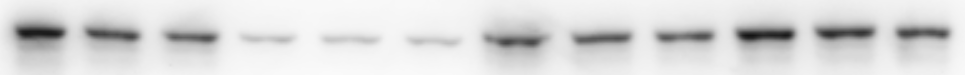

Supplement: Supplementary file 5 — Source data Fig. 4 [file 44318_2025_620_MOESM5_ESM.zip › Figure 4/4C/Western Blot Image Files/P-SHIP1 STC 20230208_145512_Ch/P-SHIP1 STC.tif]

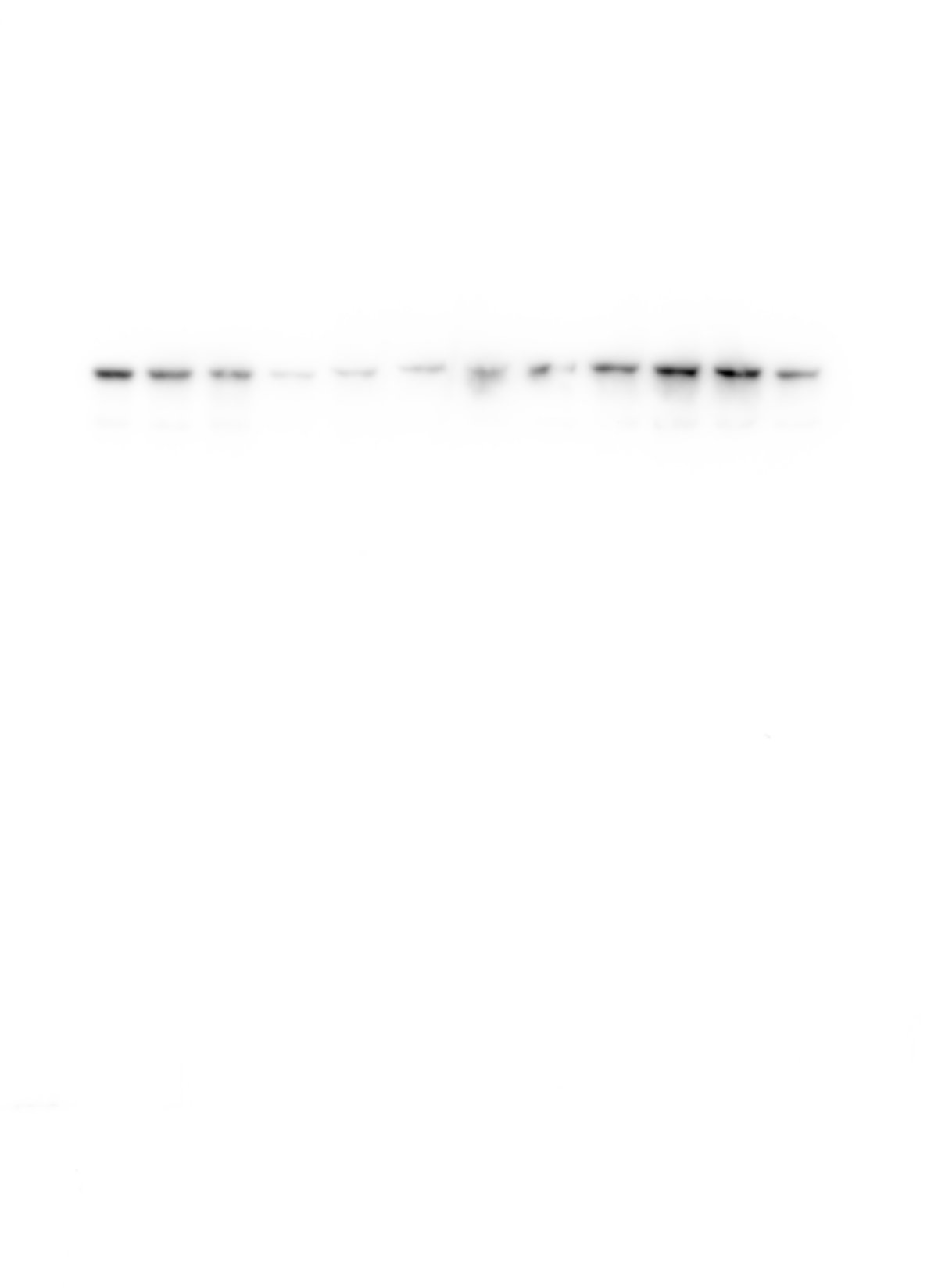

Supplement: Supplementary file 5 — Source data Fig. 4 [file 44318_2025_620_MOESM5_ESM.zip › Figure 4/4C/Western Blot Image Files/P-SHP-1 2 20230208_151055_Ch/P-SHP-1 2 20230208_151055_Ch_Chemi.tif]

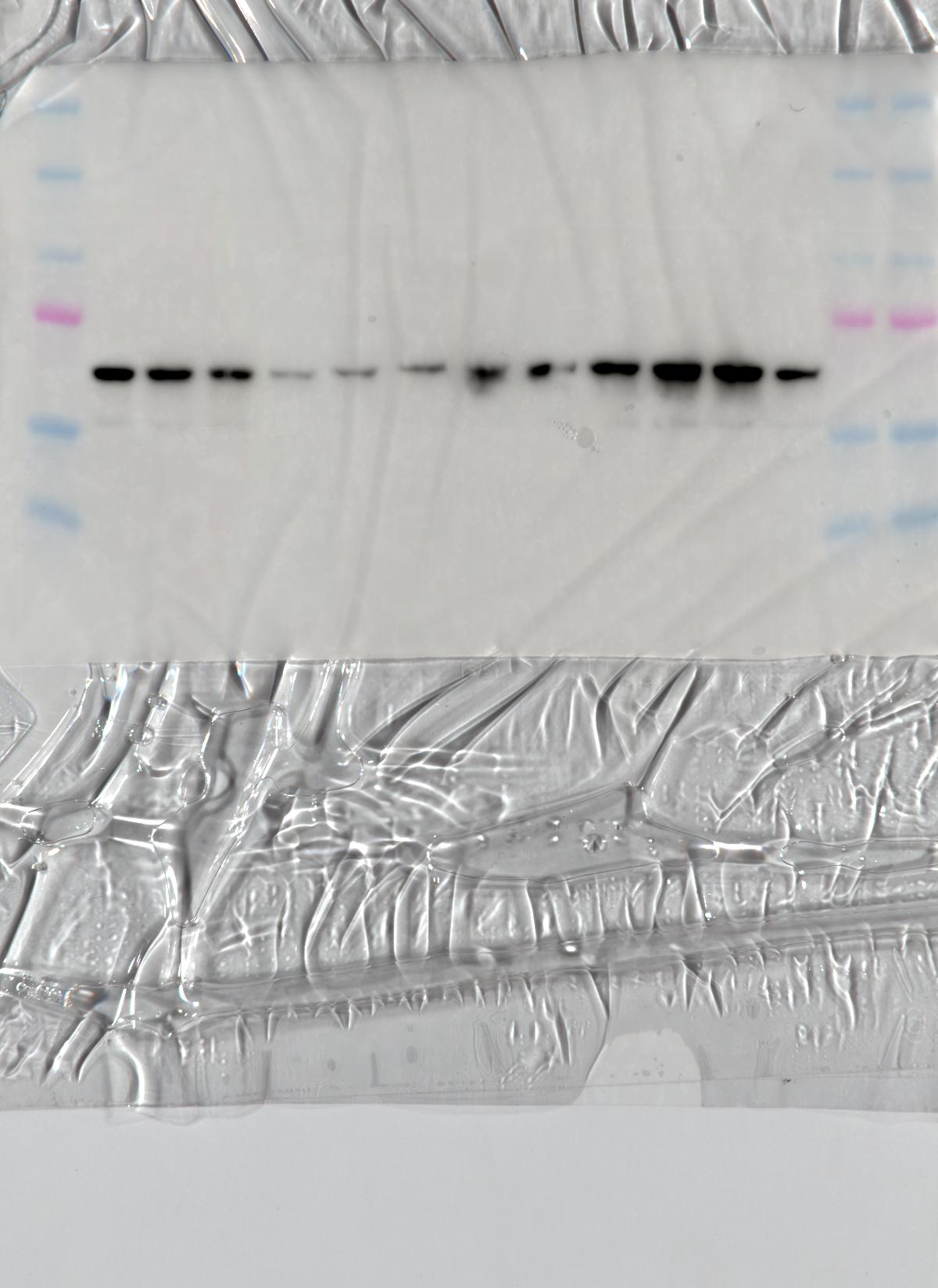

Supplement: Supplementary file 5 — Source data Fig. 4 [file 44318_2025_620_MOESM5_ESM.zip › Figure 4/4C/Western Blot Image Files/P-SHP-1 2 20230208_151055_Ch/P-SHP-1 2 20230208_151055_Ch_Chemi+Marker.jpg]

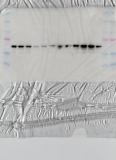

Supplement: Supplementary file 5 — Source data Fig. 4 [file 44318_2025_620_MOESM5_ESM.zip › Figure 4/4C/Western Blot Image Files/P-SHP-1 2 20230208_151055_Ch/P-SHP-1 2 20230208_151055_Ch_Thumb.jpg]

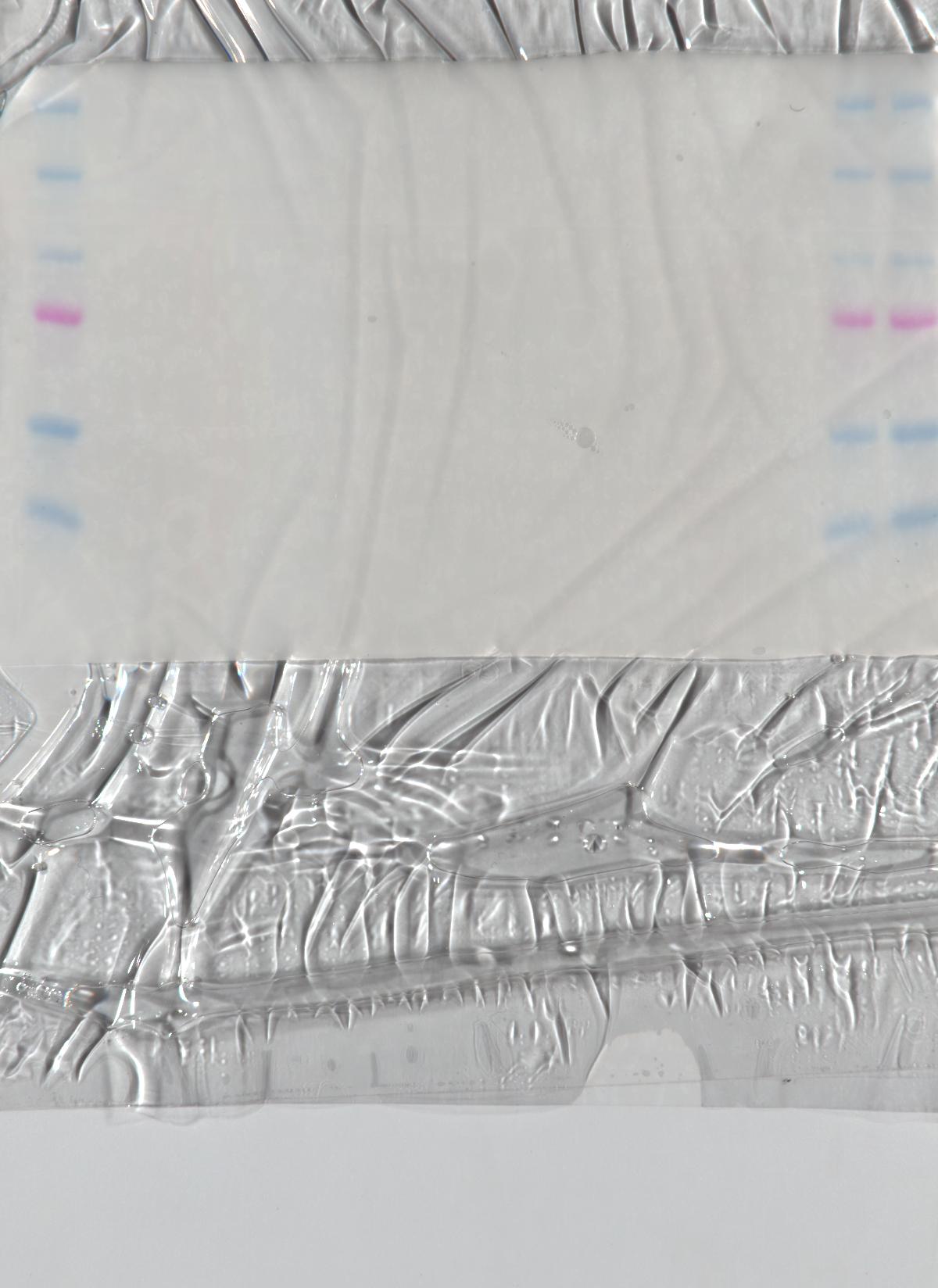

Supplement: Supplementary file 5 — Source data Fig. 4 [file 44318_2025_620_MOESM5_ESM.zip › Figure 4/4C/Western Blot Image Files/P-SHP-1 2 20230208_151055_Ch/P-SHP-1 2 20230208_151055_Ch-Marker.jpg]

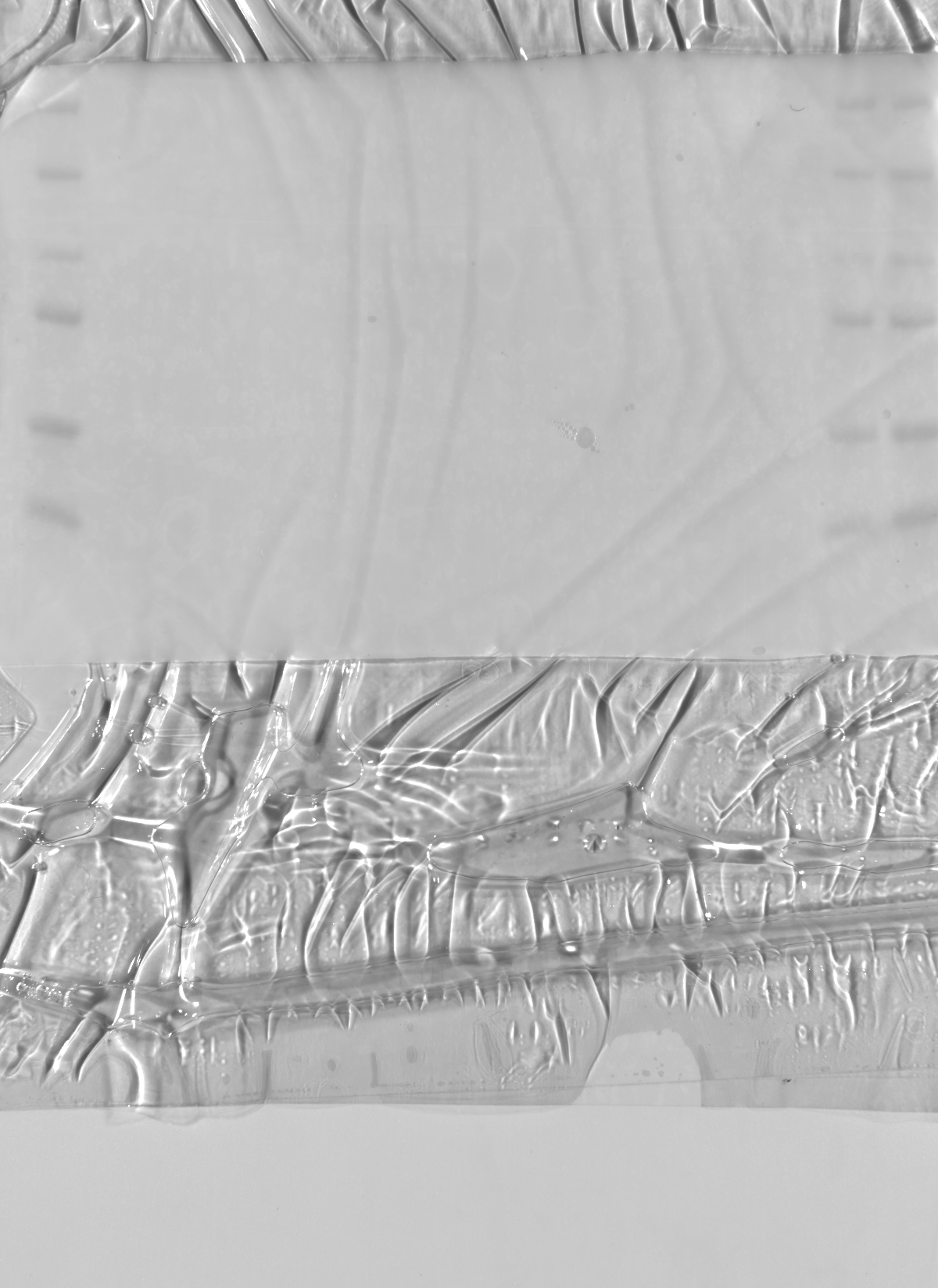

Supplement: Supplementary file 5 — Source data Fig. 4 [file 44318_2025_620_MOESM5_ESM.zip › Figure 4/4C/Western Blot Image Files/P-SHP-1 2 20230208_151055_Ch/P-SHP-1 2 20230208_151055_Ch-Marker.tif]

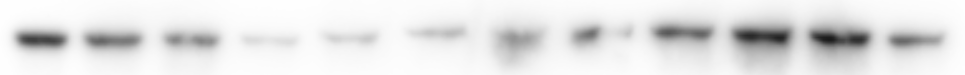

Supplement: Supplementary file 5 — Source data Fig. 4 [file 44318_2025_620_MOESM5_ESM.zip › Figure 4/4C/Western Blot Image Files/P-SHP-1 2 20230208_151055_Ch/P-SHP-1 2.tif]

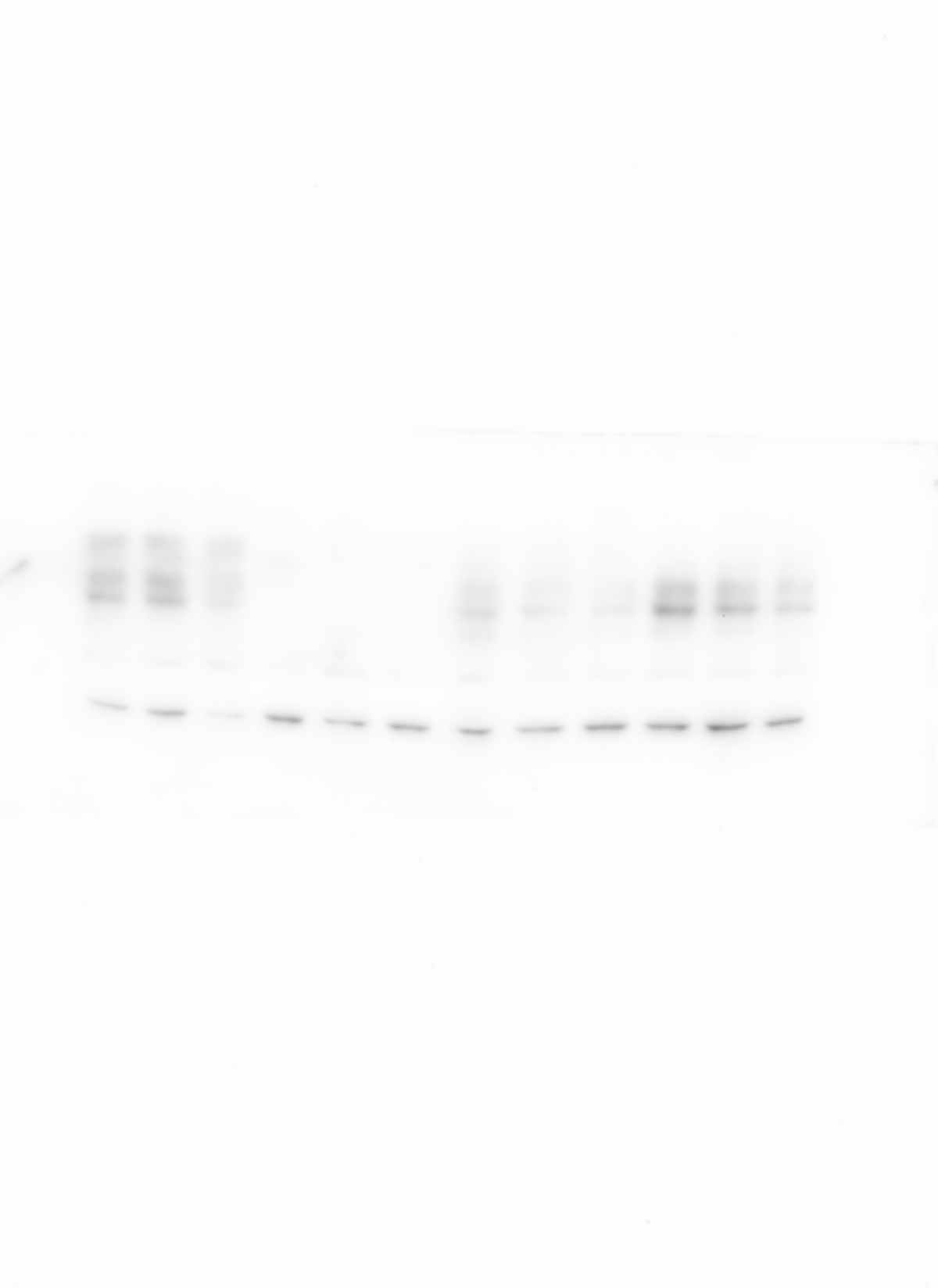

Supplement: Supplementary file 5 — Source data Fig. 4 [file 44318_2025_620_MOESM5_ESM.zip › Figure 4/4C/Western Blot Image Files/P-SHP-2 Y542 20230208_135035_Ch/P-SHP-2 Y542 20230208_135035_Ch_Chemi.tif]

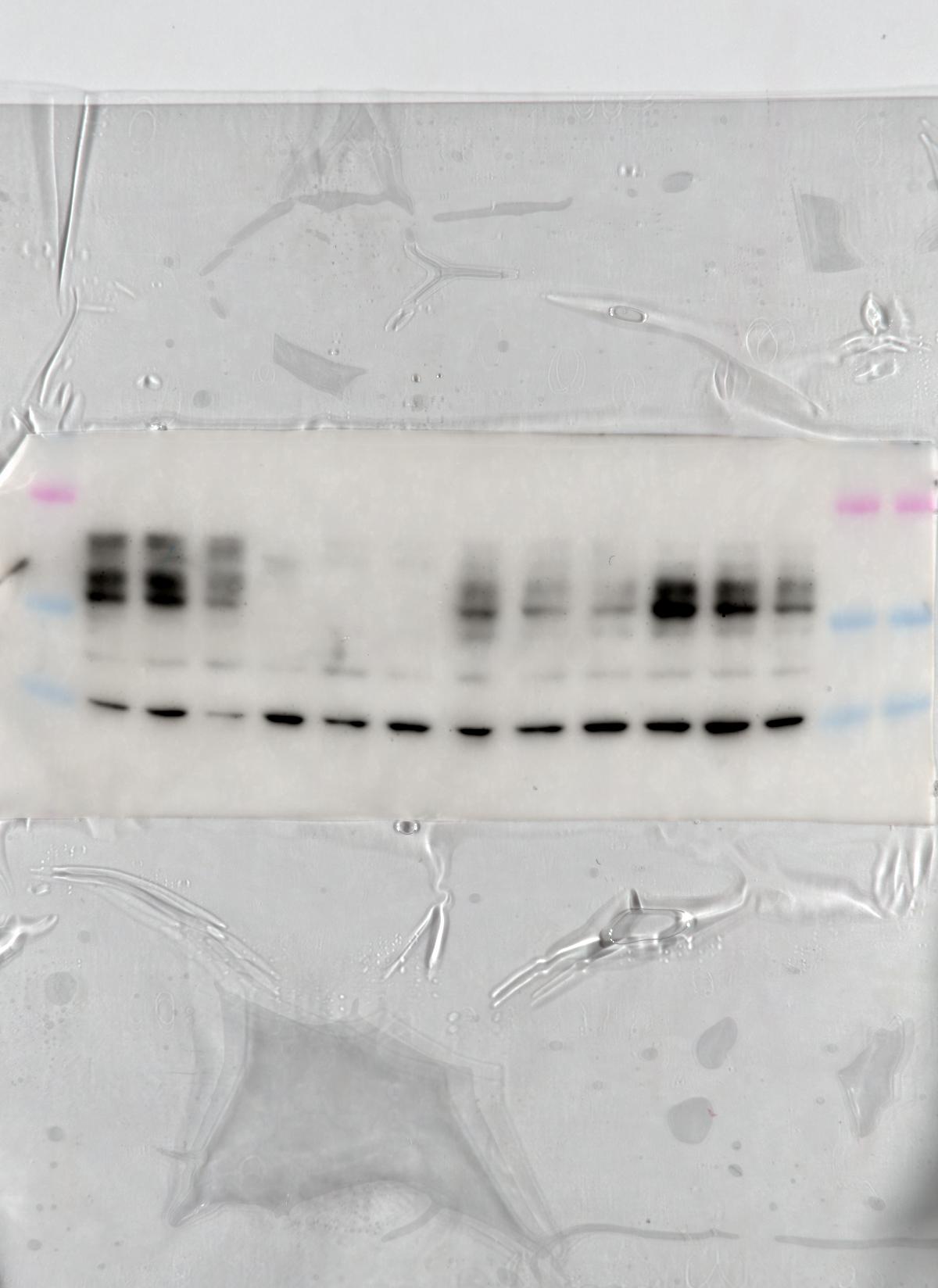

Supplement: Supplementary file 5 — Source data Fig. 4 [file 44318_2025_620_MOESM5_ESM.zip › Figure 4/4C/Western Blot Image Files/P-SHP-2 Y542 20230208_135035_Ch/P-SHP-2 Y542 20230208_135035_Ch_Chemi+Marker.jpg]

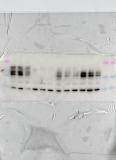

Supplement: Supplementary file 5 — Source data Fig. 4 [file 44318_2025_620_MOESM5_ESM.zip › Figure 4/4C/Western Blot Image Files/P-SHP-2 Y542 20230208_135035_Ch/P-SHP-2 Y542 20230208_135035_Ch_Thumb.jpg]

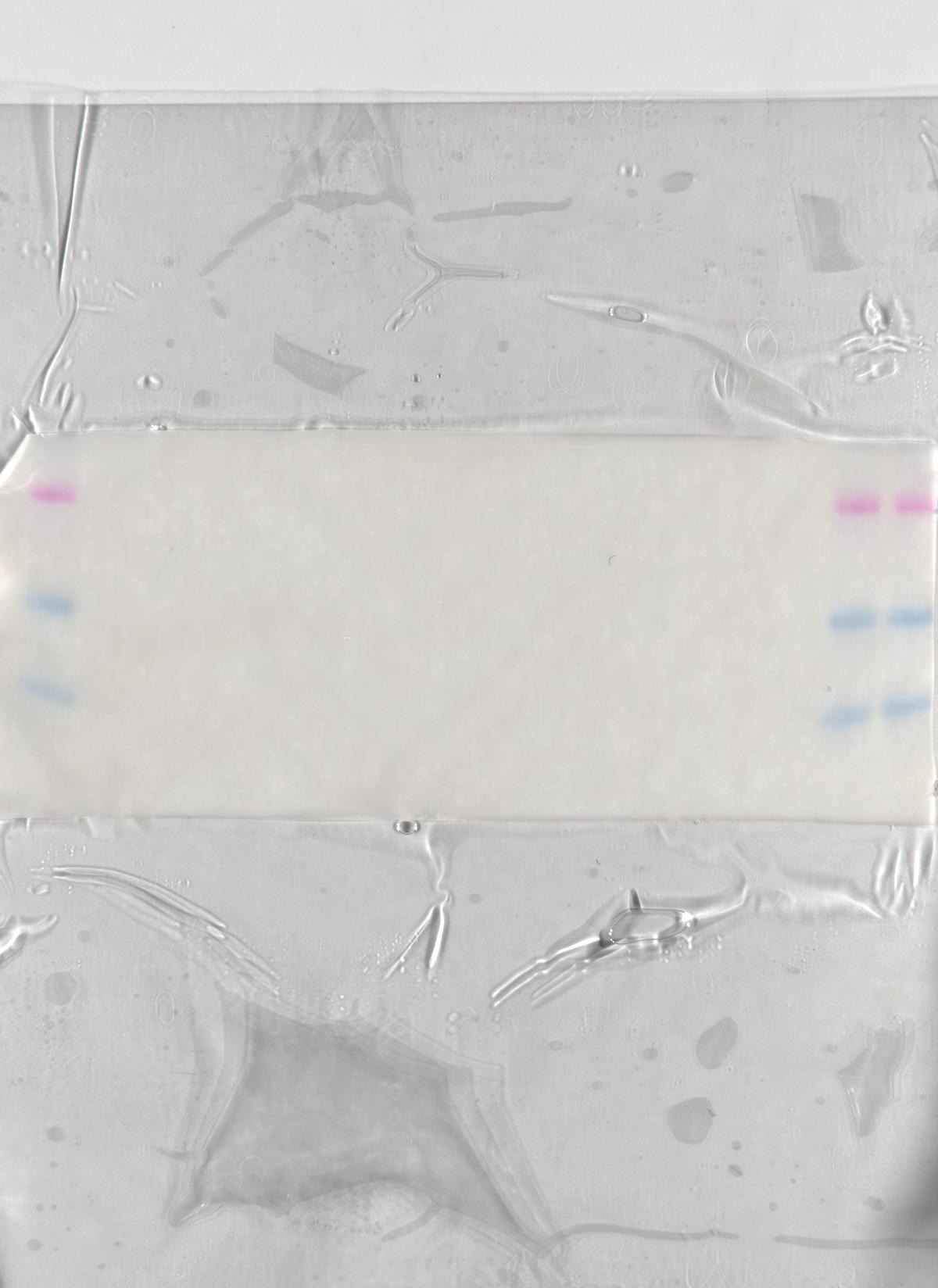

Supplement: Supplementary file 5 — Source data Fig. 4 [file 44318_2025_620_MOESM5_ESM.zip › Figure 4/4C/Western Blot Image Files/P-SHP-2 Y542 20230208_135035_Ch/P-SHP-2 Y542 20230208_135035_Ch-Marker.jpg]

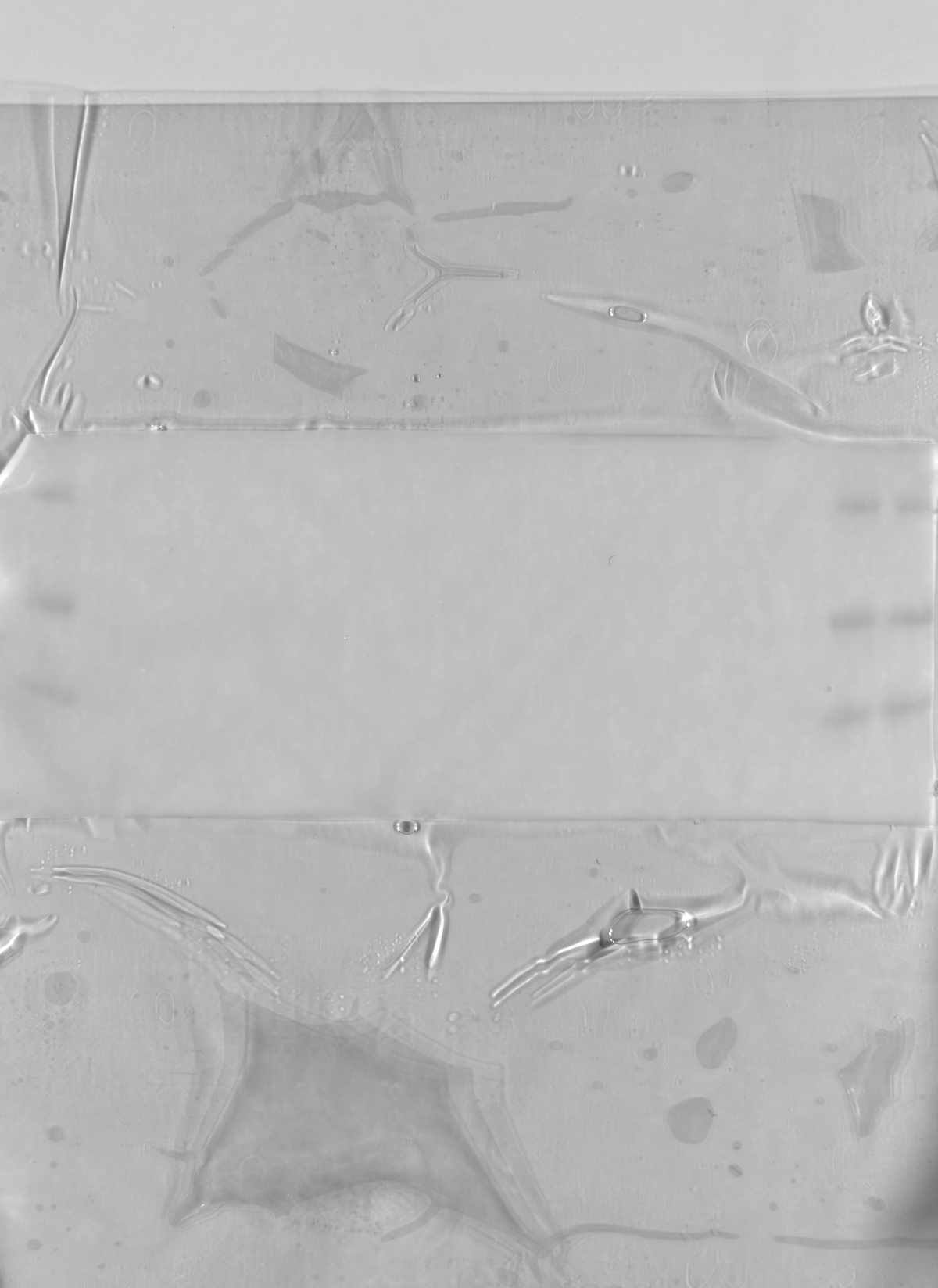

Supplement: Supplementary file 5 — Source data Fig. 4 [file 44318_2025_620_MOESM5_ESM.zip › Figure 4/4C/Western Blot Image Files/P-SHP-2 Y542 20230208_135035_Ch/P-SHP-2 Y542 20230208_135035_Ch-Marker.tif]

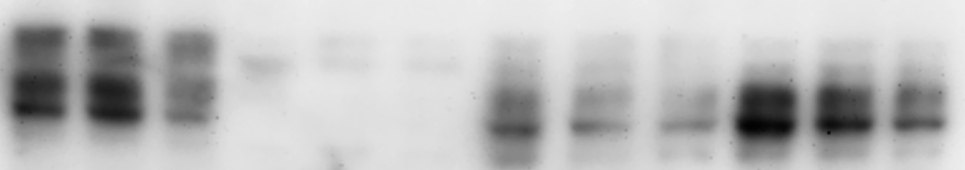

Supplement: Supplementary file 5 — Source data Fig. 4 [file 44318_2025_620_MOESM5_ESM.zip › Figure 4/4C/Western Blot Image Files/P-SHP-2 Y542 20230208_135035_Ch/P-SHP-2 Y542.tif]

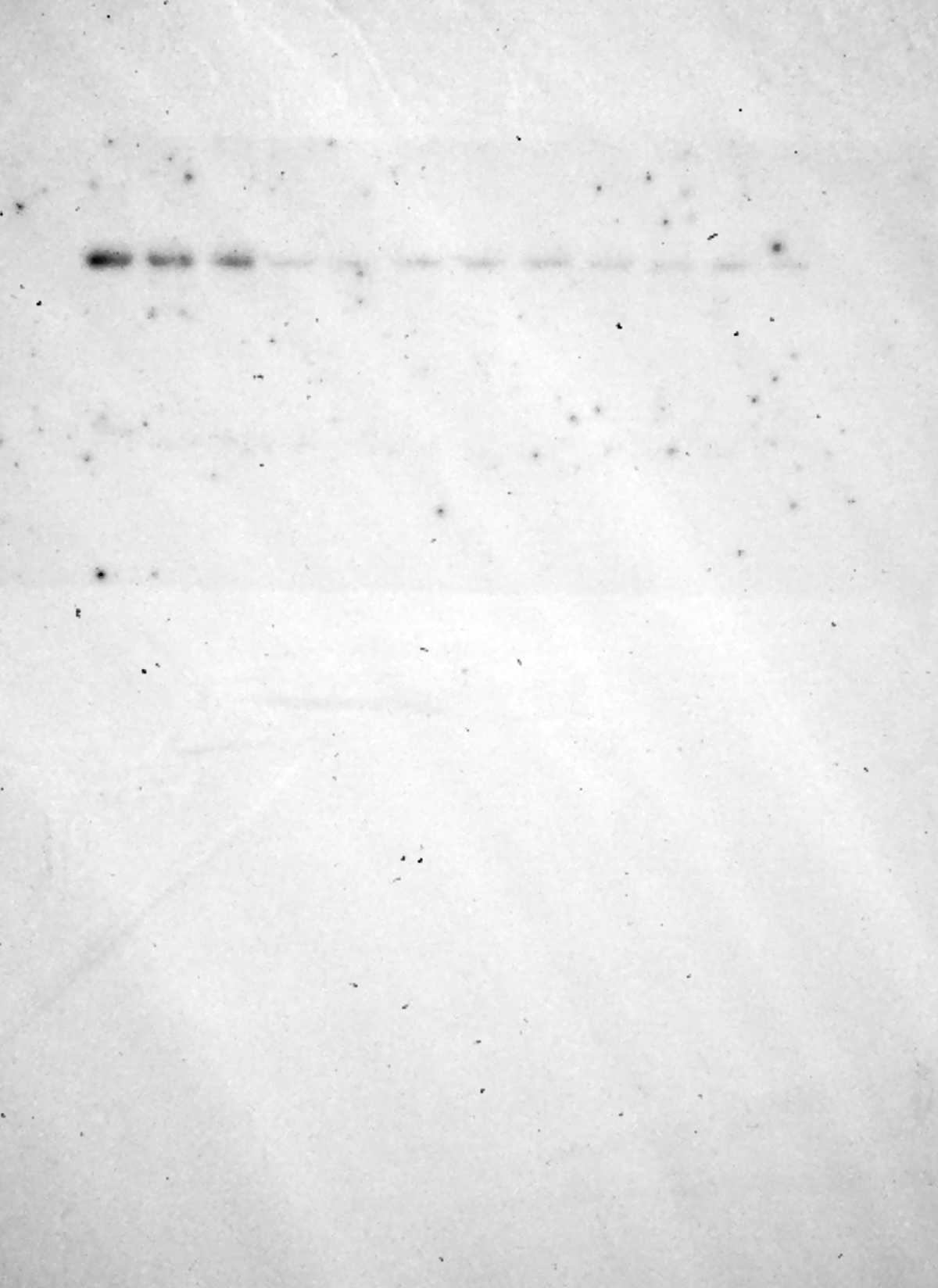

Supplement: Supplementary file 5 — Source data Fig. 4 [file 44318_2025_620_MOESM5_ESM.zip › Figure 4/4C/Western Blot Image Files/P-SHP-2 Y580 20230208_132431_Ch/P-SHP-2 Y580 20230208_132431_Ch_Chemi.tif]

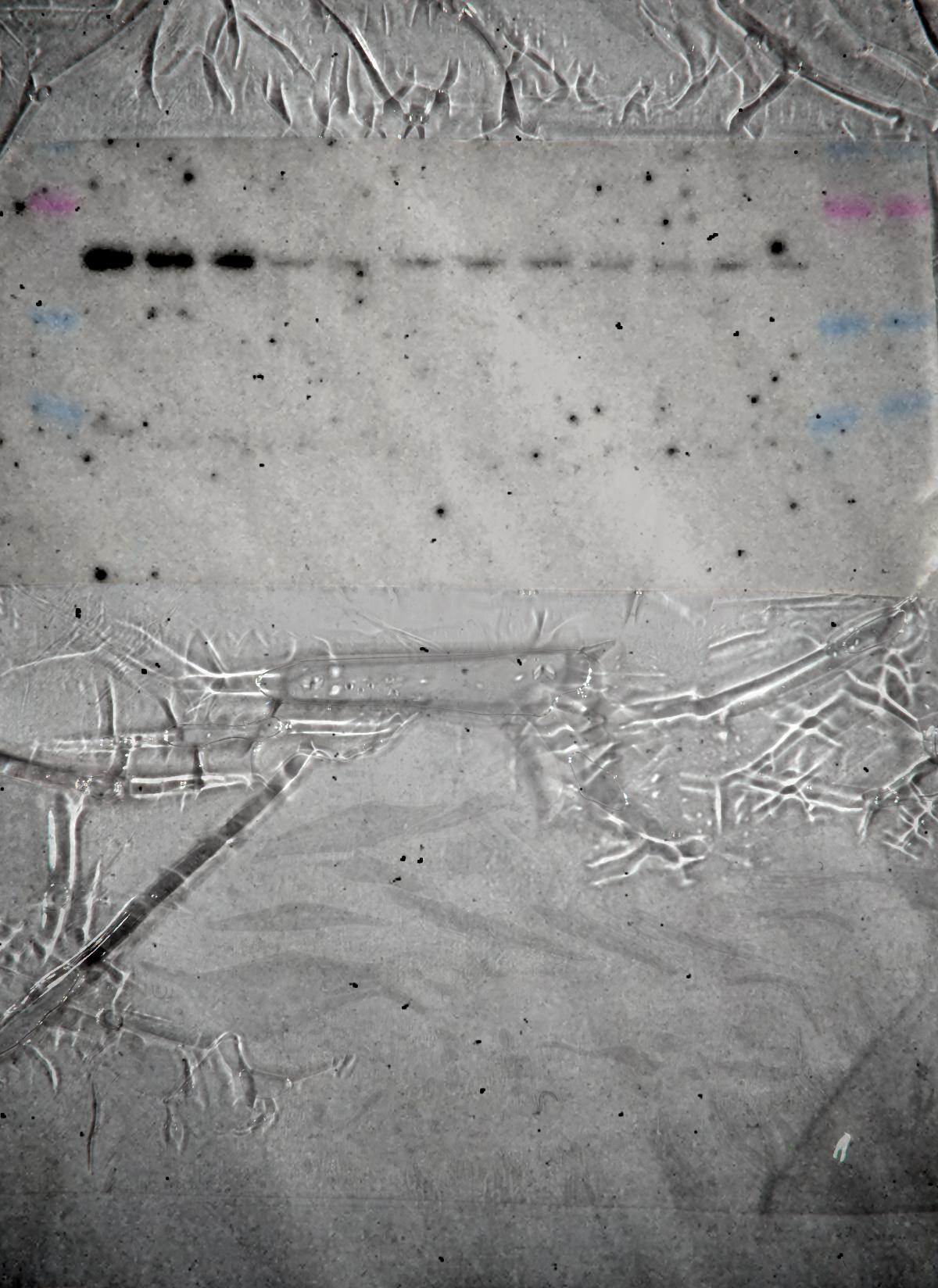

Supplement: Supplementary file 5 — Source data Fig. 4 [file 44318_2025_620_MOESM5_ESM.zip › Figure 4/4C/Western Blot Image Files/P-SHP-2 Y580 20230208_132431_Ch/P-SHP-2 Y580 20230208_132431_Ch_Chemi+Marker.jpg]

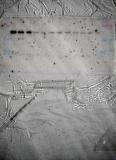

Supplement: Supplementary file 5 — Source data Fig. 4 [file 44318_2025_620_MOESM5_ESM.zip › Figure 4/4C/Western Blot Image Files/P-SHP-2 Y580 20230208_132431_Ch/P-SHP-2 Y580 20230208_132431_Ch_Thumb.jpg]

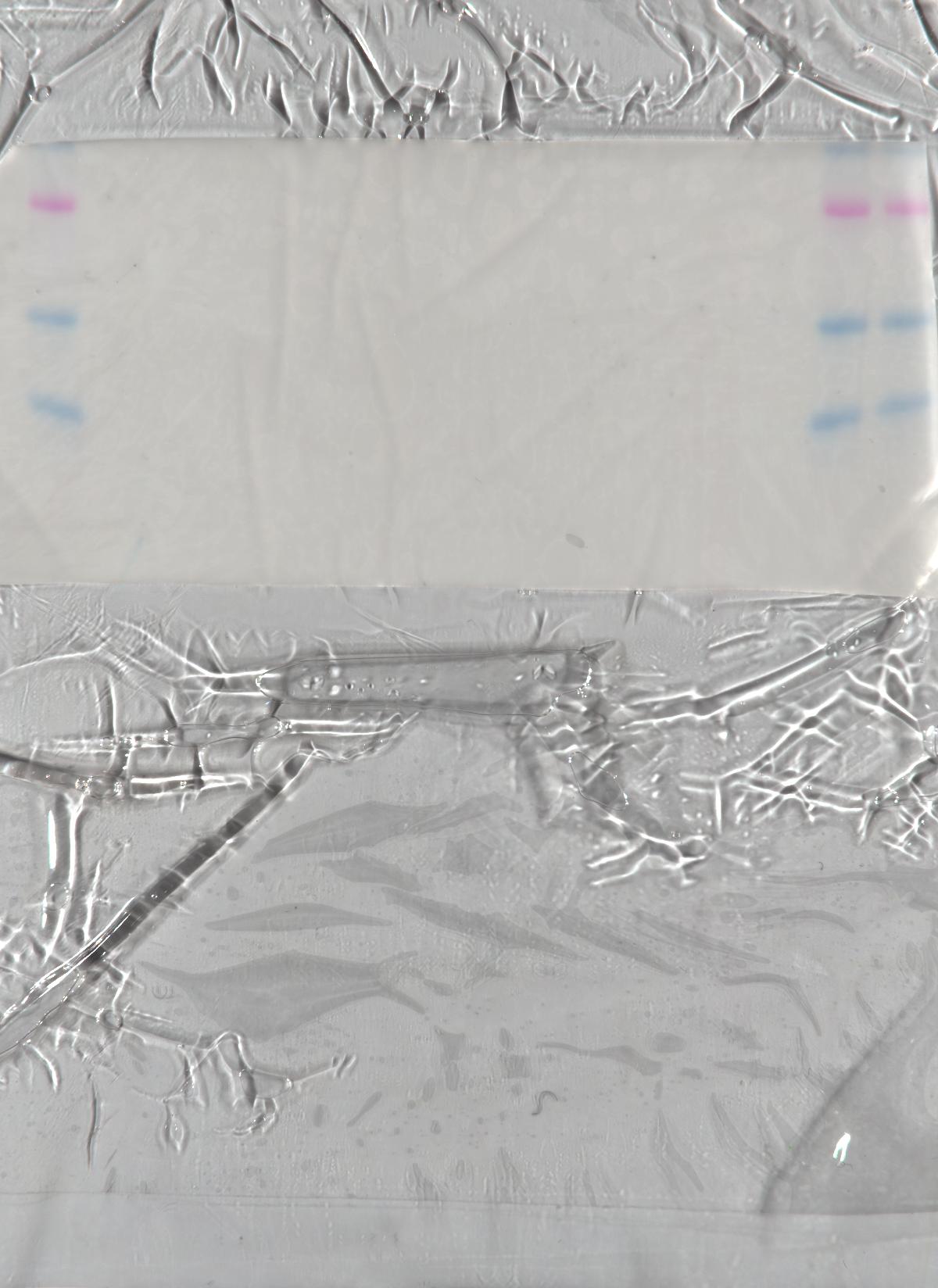

Supplement: Supplementary file 5 — Source data Fig. 4 [file 44318_2025_620_MOESM5_ESM.zip › Figure 4/4C/Western Blot Image Files/P-SHP-2 Y580 20230208_132431_Ch/P-SHP-2 Y580 20230208_132431_Ch-Marker.jpg]

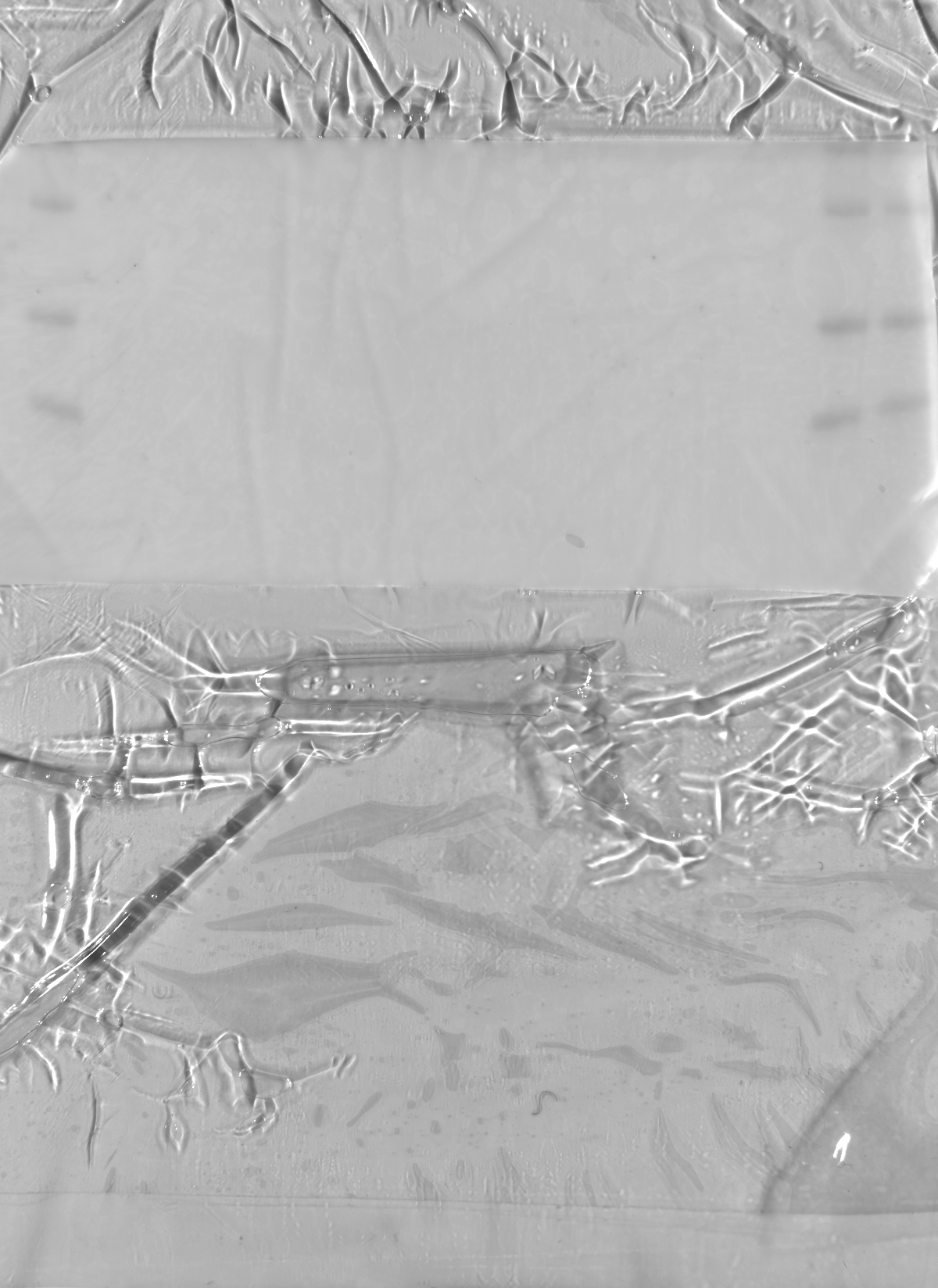

Supplement: Supplementary file 5 — Source data Fig. 4 [file 44318_2025_620_MOESM5_ESM.zip › Figure 4/4C/Western Blot Image Files/P-SHP-2 Y580 20230208_132431_Ch/P-SHP-2 Y580 20230208_132431_Ch-Marker.tif]

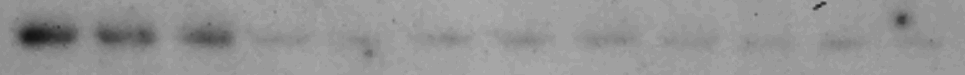

Supplement: Supplementary file 5 — Source data Fig. 4 [file 44318_2025_620_MOESM5_ESM.zip › Figure 4/4C/Western Blot Image Files/P-SHP-2 Y580 20230208_132431_Ch/P-SHP-2 Y580.tif]

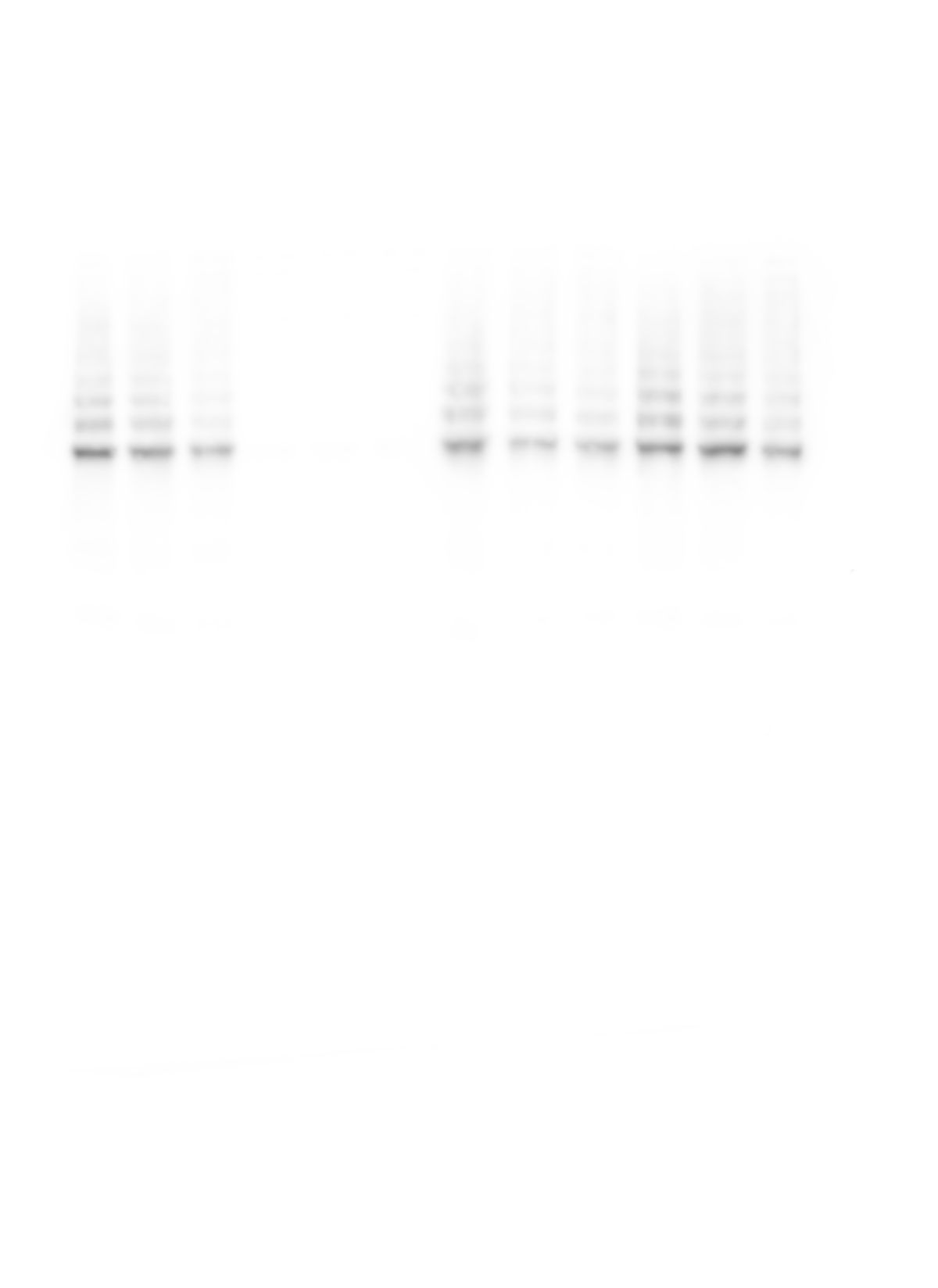

Supplement: Supplementary file 5 — Source data Fig. 4 [file 44318_2025_620_MOESM5_ESM.zip › Figure 4/4C/Western Blot Image Files/P-Syk Y346 2 20230208_145025_Ch/P-Syk Y346 2 20230208_145025_Ch_Chemi.tif]

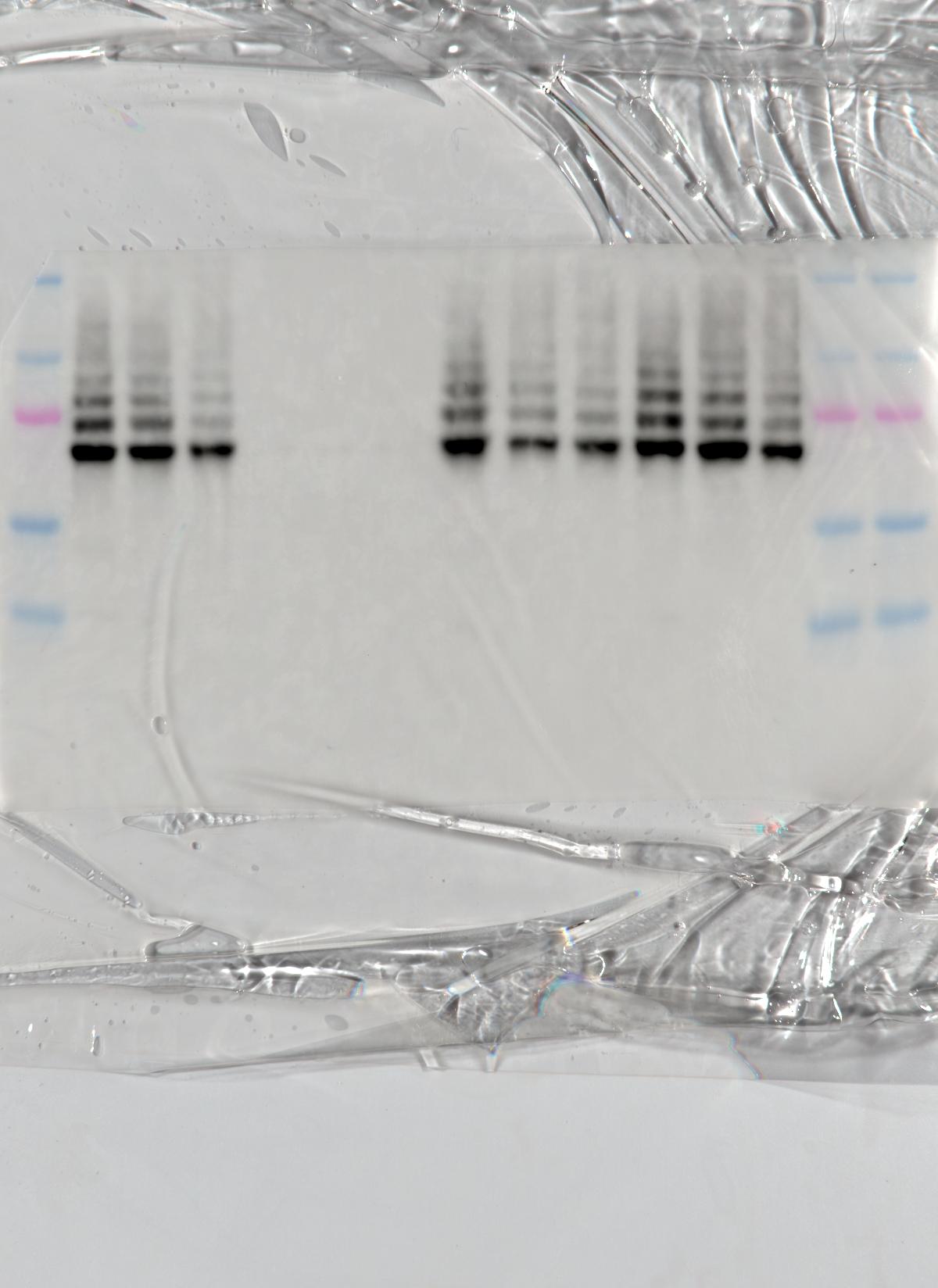

Supplement: Supplementary file 5 — Source data Fig. 4 [file 44318_2025_620_MOESM5_ESM.zip › Figure 4/4C/Western Blot Image Files/P-Syk Y346 2 20230208_145025_Ch/P-Syk Y346 2 20230208_145025_Ch_Chemi+Marker.jpg]

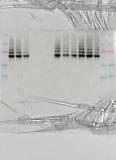

Supplement: Supplementary file 5 — Source data Fig. 4 [file 44318_2025_620_MOESM5_ESM.zip › Figure 4/4C/Western Blot Image Files/P-Syk Y346 2 20230208_145025_Ch/P-Syk Y346 2 20230208_145025_Ch_Thumb.jpg]

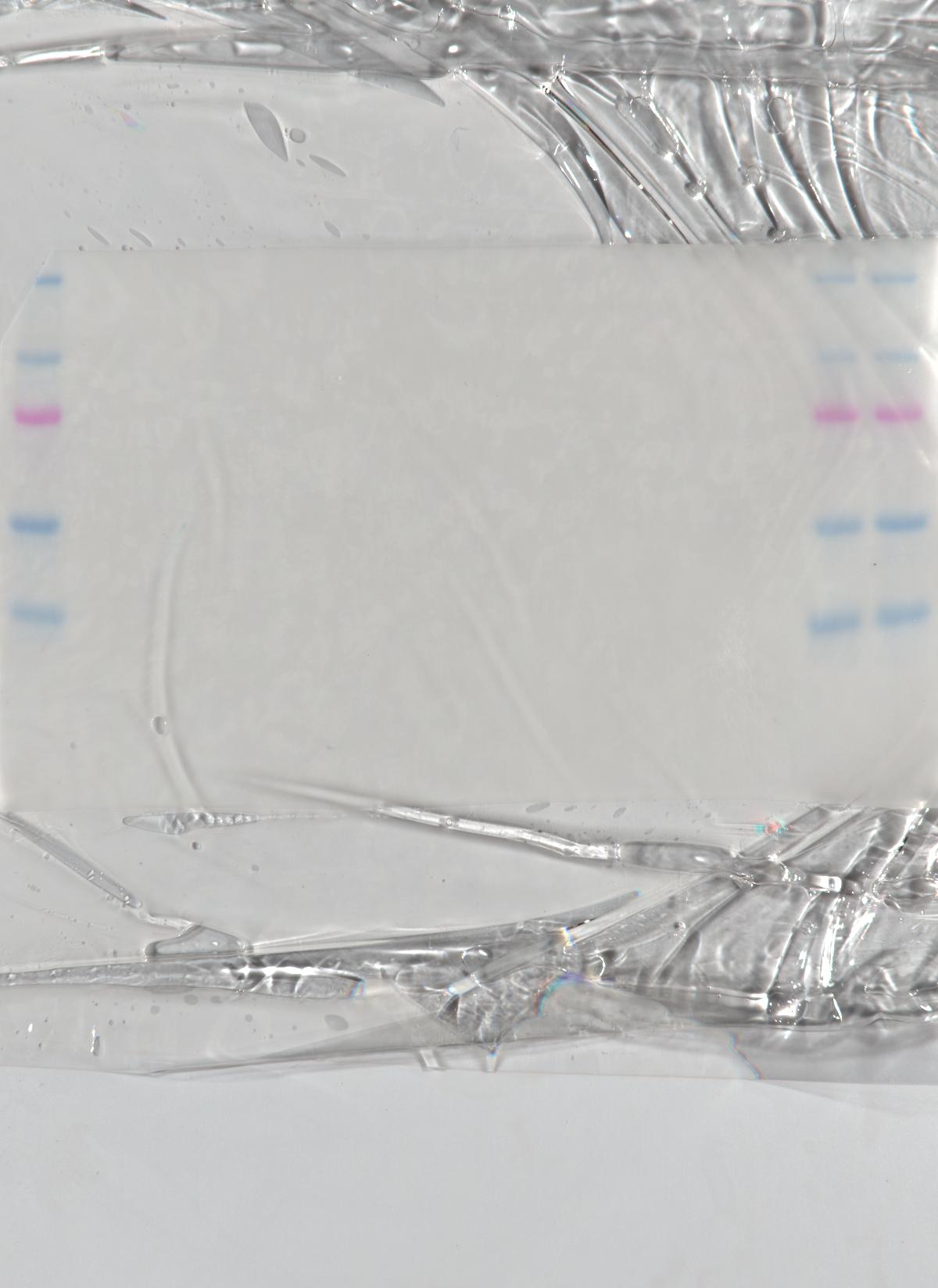

Supplement: Supplementary file 5 — Source data Fig. 4 [file 44318_2025_620_MOESM5_ESM.zip › Figure 4/4C/Western Blot Image Files/P-Syk Y346 2 20230208_145025_Ch/P-Syk Y346 2 20230208_145025_Ch-Marker.jpg]

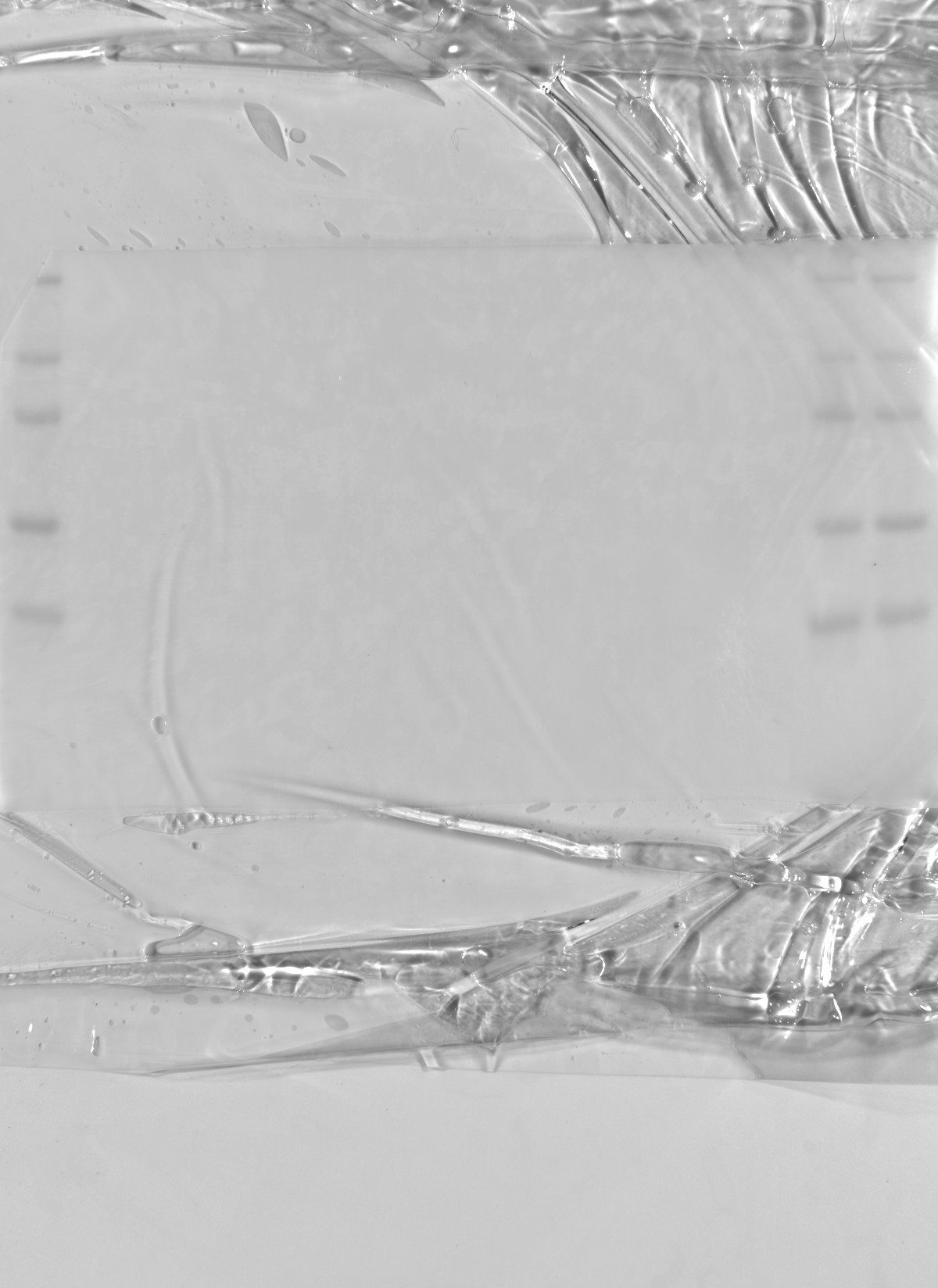

Supplement: Supplementary file 5 — Source data Fig. 4 [file 44318_2025_620_MOESM5_ESM.zip › Figure 4/4C/Western Blot Image Files/P-Syk Y346 2 20230208_145025_Ch/P-Syk Y346 2 20230208_145025_Ch-Marker.tif]

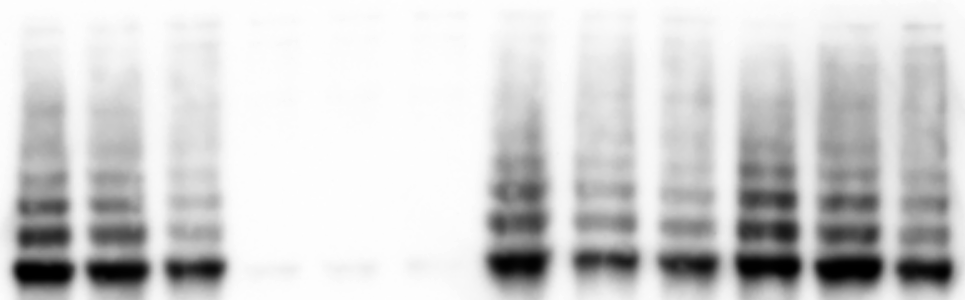

Supplement: Supplementary file 5 — Source data Fig. 4 [file 44318_2025_620_MOESM5_ESM.zip › Figure 4/4C/Western Blot Image Files/P-Syk Y346 2 20230208_145025_Ch/P-Syk Y346 2.tif]

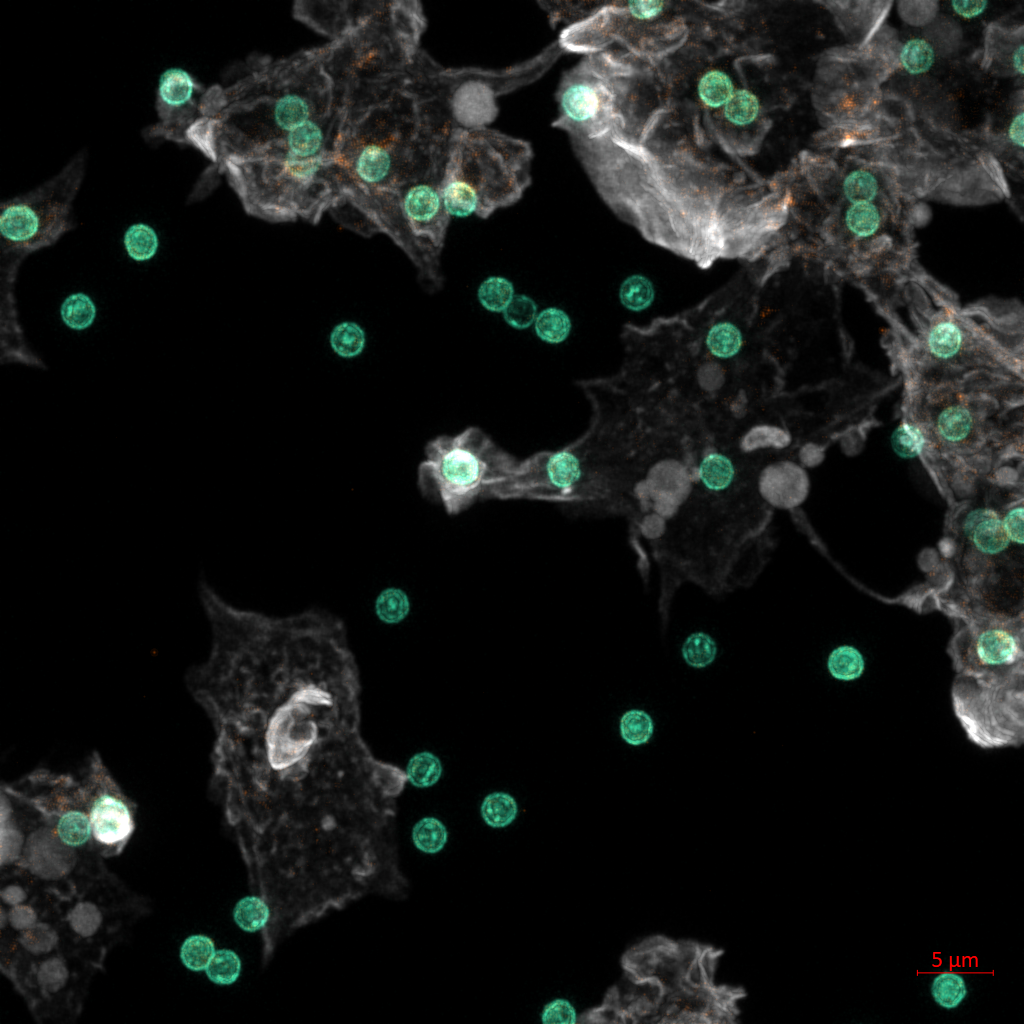

Supplement: Supplementary file 5 — Source data Fig. 4 [file 44318_2025_620_MOESM5_ESM.zip › Figure 4/4E/PSHIP1 2WA ruler_c1+2+3.tif]

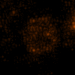

Supplement: Supplementary file 5 — Source data Fig. 4 [file 44318_2025_620_MOESM5_ESM.zip › Figure 4/4E/PSHIP1 2WA_c1 copy 2.tif]

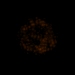

Supplement: Supplementary file 5 — Source data Fig. 4 [file 44318_2025_620_MOESM5_ESM.zip › Figure 4/4E/PSHIP1 2WA_c1 copy 3.tif]

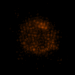

Supplement: Supplementary file 5 — Source data Fig. 4 [file 44318_2025_620_MOESM5_ESM.zip › Figure 4/4E/PSHIP1 2WA_c1 copy.tif]

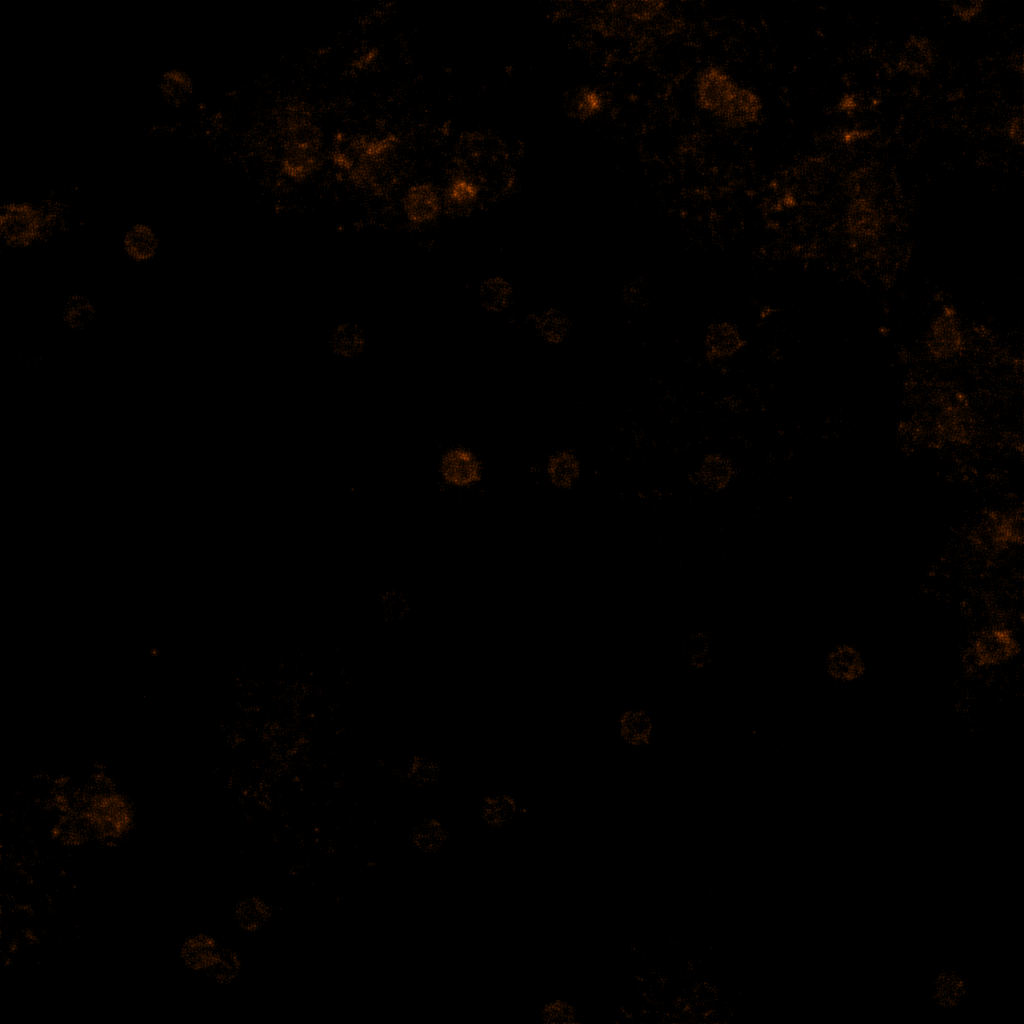

Supplement: Supplementary file 5 — Source data Fig. 4 [file 44318_2025_620_MOESM5_ESM.zip › Figure 4/4E/PSHIP1 2WA_c1.tif]

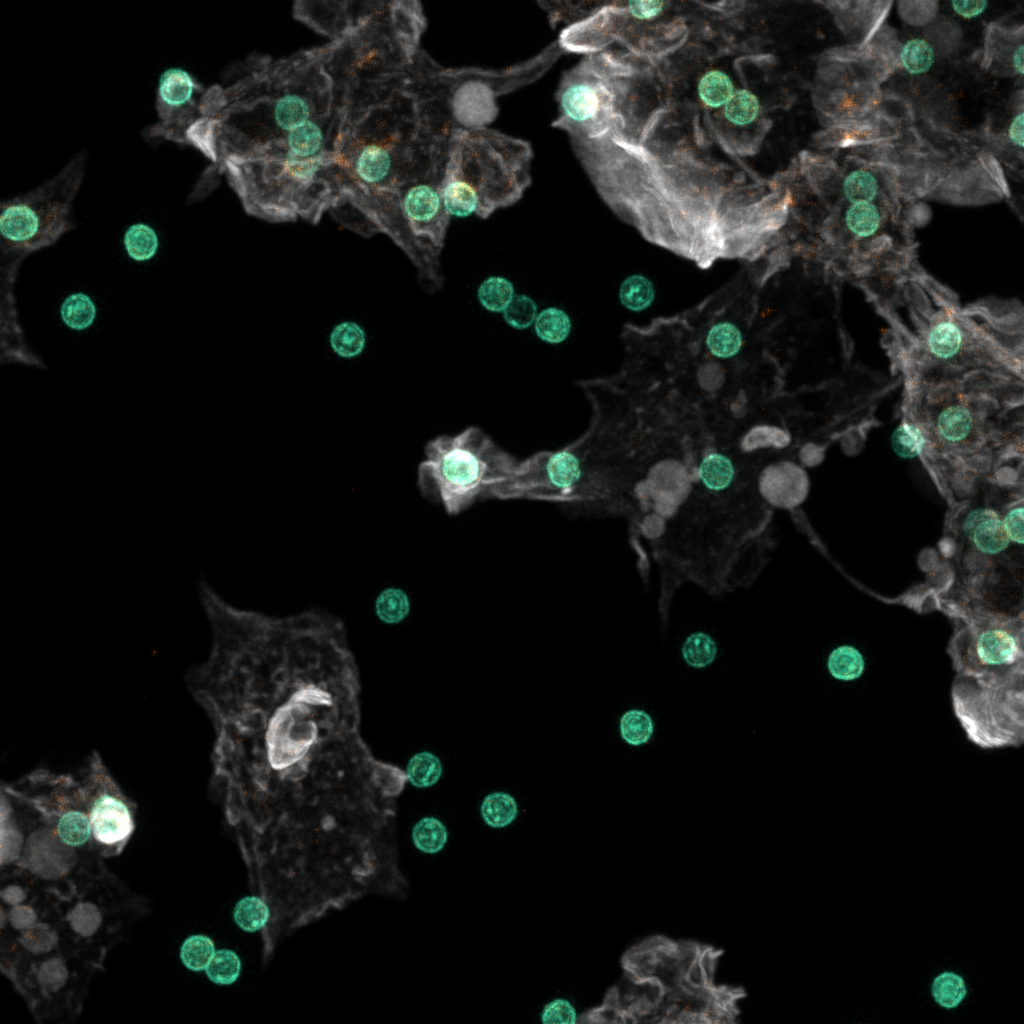

Supplement: Supplementary file 5 — Source data Fig. 4 [file 44318_2025_620_MOESM5_ESM.zip › Figure 4/4E/PSHIP1 2WA_c1+2+3.tif]

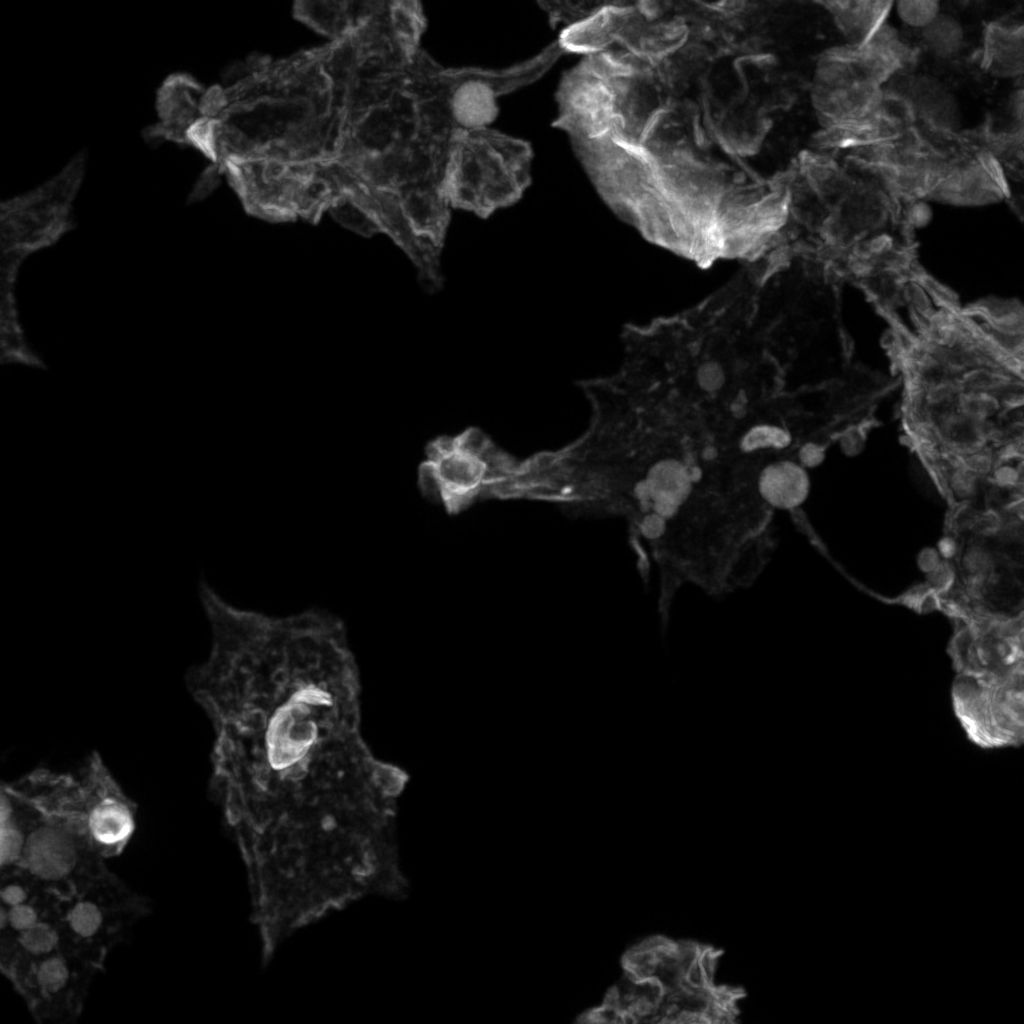

Supplement: Supplementary file 5 — Source data Fig. 4 [file 44318_2025_620_MOESM5_ESM.zip › Figure 4/4E/PSHIP1 2WA_c2.tif]

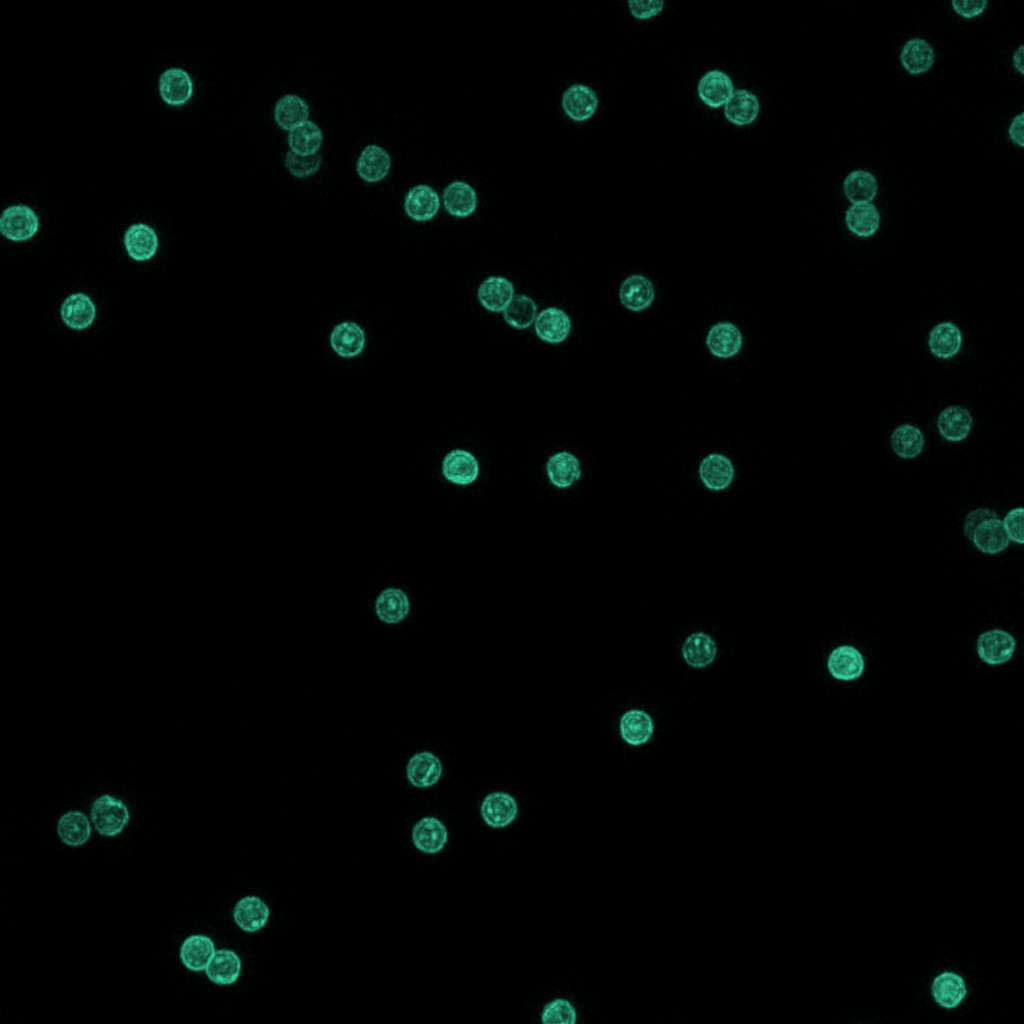

Supplement: Supplementary file 5 — Source data Fig. 4 [file 44318_2025_620_MOESM5_ESM.zip › Figure 4/4E/PSHIP1 2WA_c3.tif]

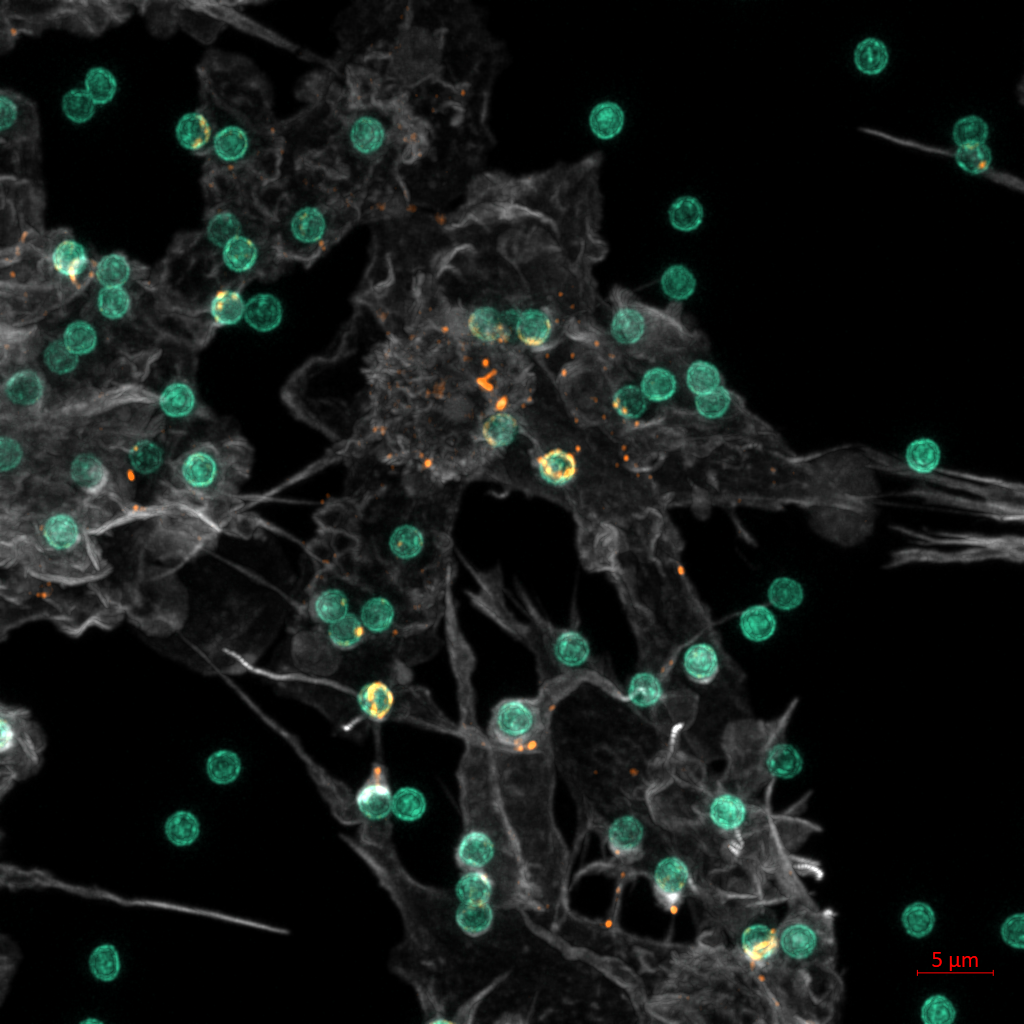

Supplement: Supplementary file 5 — Source data Fig. 4 [file 44318_2025_620_MOESM5_ESM.zip › Figure 4/4E/PSHIP1 C9 ruler_c1+2+3.tif]

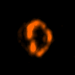

Supplement: Supplementary file 5 — Source data Fig. 4 [file 44318_2025_620_MOESM5_ESM.zip › Figure 4/4E/PSHIP1 C9_c1 copy 2.tif]

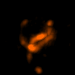

Supplement: Supplementary file 5 — Source data Fig. 4 [file 44318_2025_620_MOESM5_ESM.zip › Figure 4/4E/PSHIP1 C9_c1 copy 3.tif]

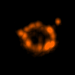

Supplement: Supplementary file 5 — Source data Fig. 4 [file 44318_2025_620_MOESM5_ESM.zip › Figure 4/4E/PSHIP1 C9_c1 copy.tif]

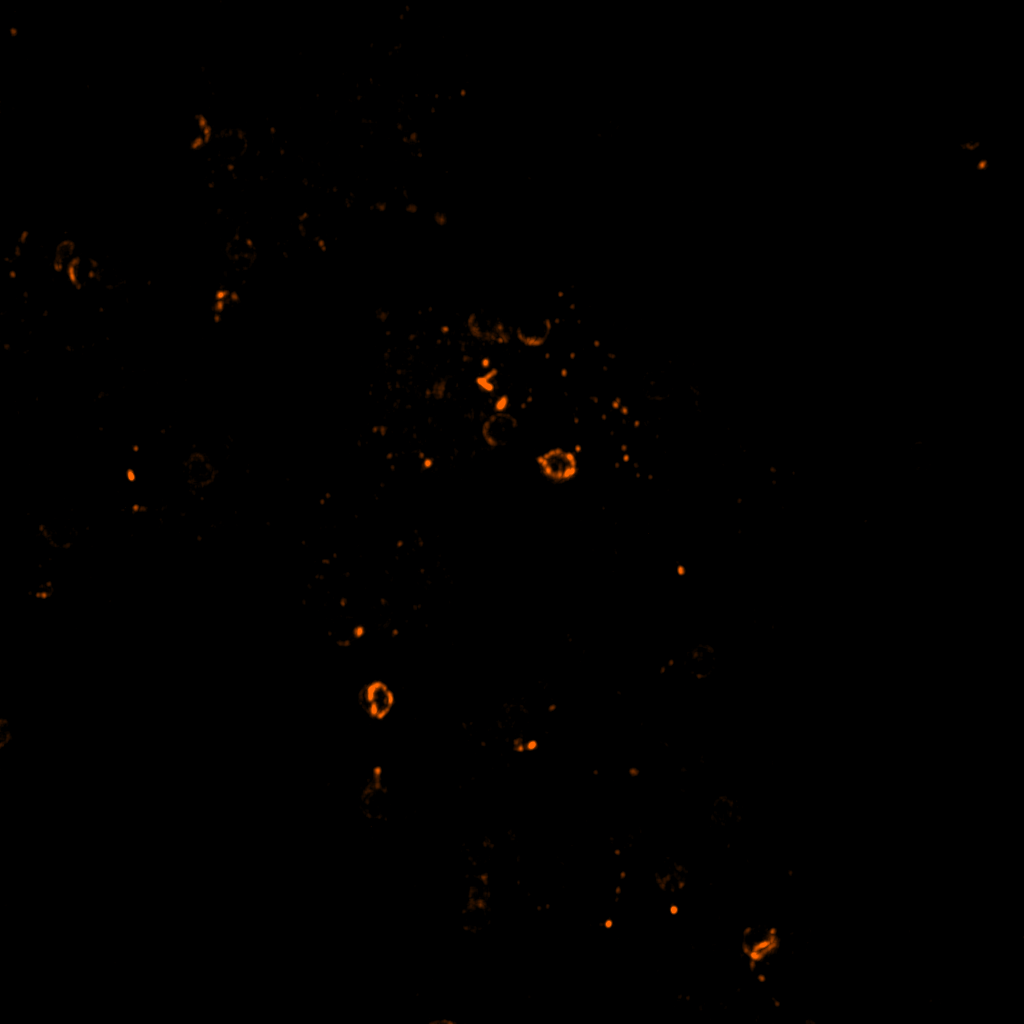

Supplement: Supplementary file 5 — Source data Fig. 4 [file 44318_2025_620_MOESM5_ESM.zip › Figure 4/4E/PSHIP1 C9_c1.tif]

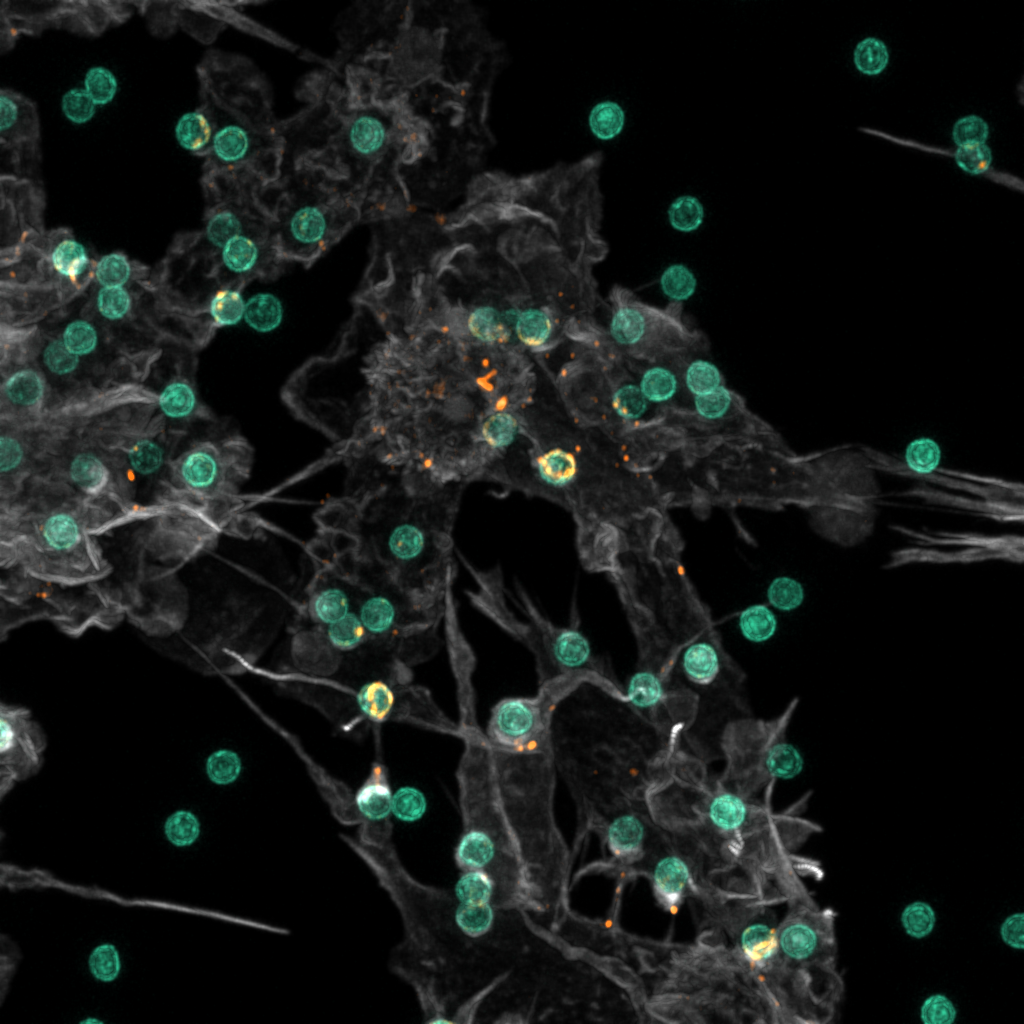

Supplement: Supplementary file 5 — Source data Fig. 4 [file 44318_2025_620_MOESM5_ESM.zip › Figure 4/4E/PSHIP1 C9_c1+2+3.tif]

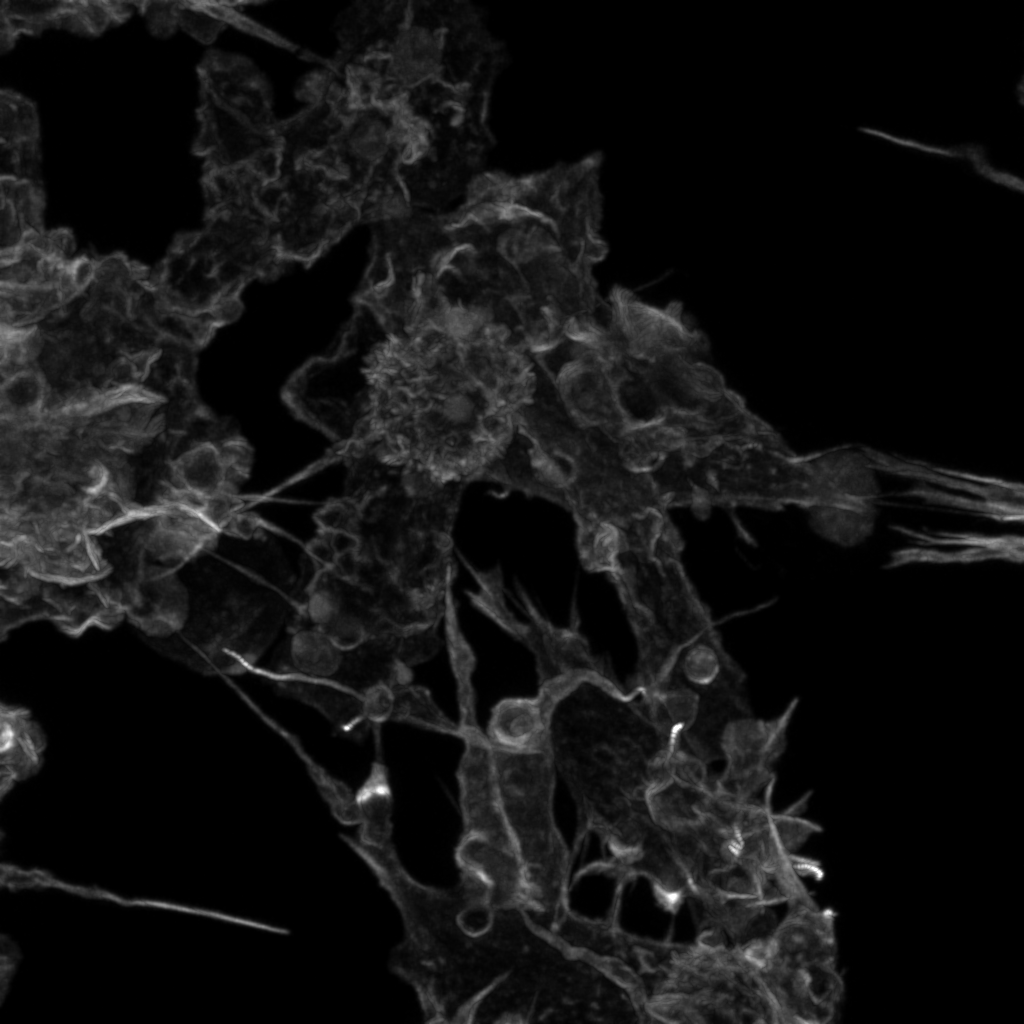

Supplement: Supplementary file 5 — Source data Fig. 4 [file 44318_2025_620_MOESM5_ESM.zip › Figure 4/4E/PSHIP1 C9_c2.tif]

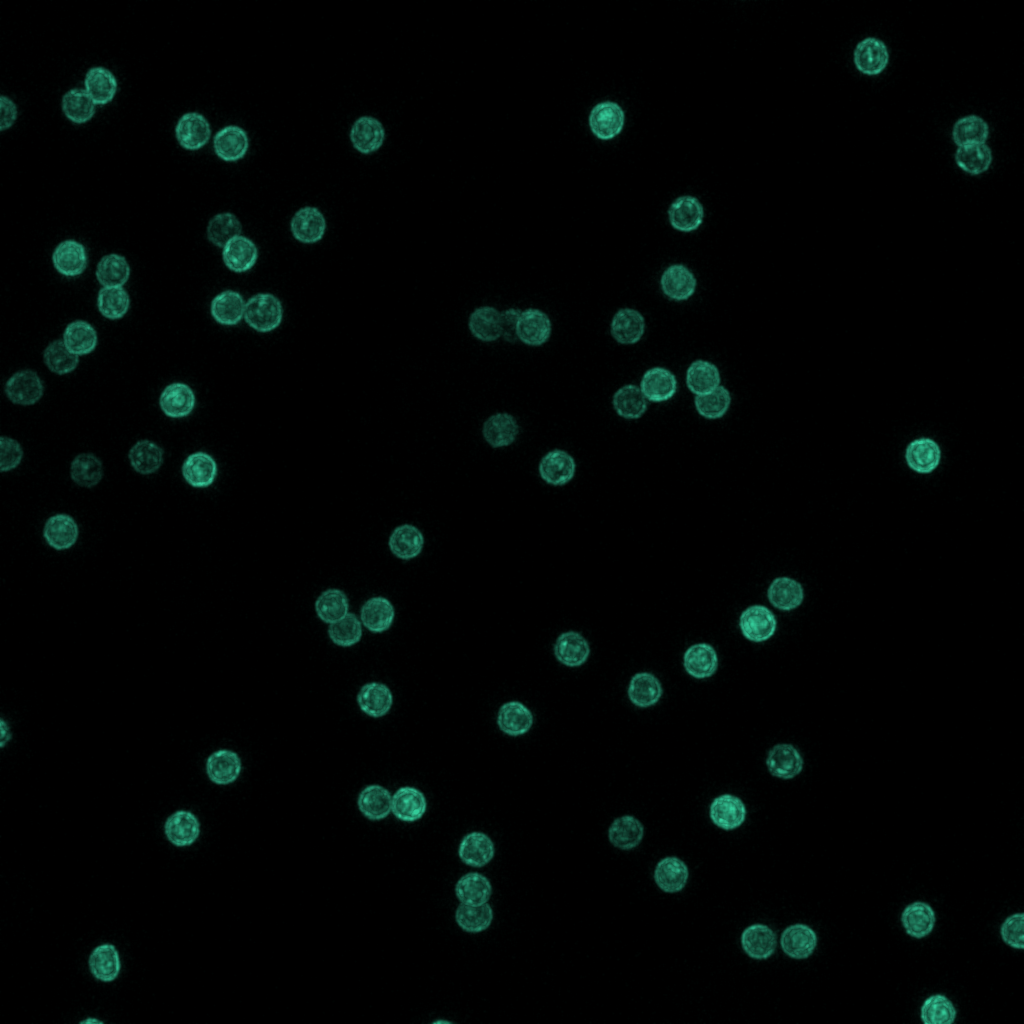

Supplement: Supplementary file 5 — Source data Fig. 4 [file 44318_2025_620_MOESM5_ESM.zip › Figure 4/4E/PSHIP1 C9_c3.tif]

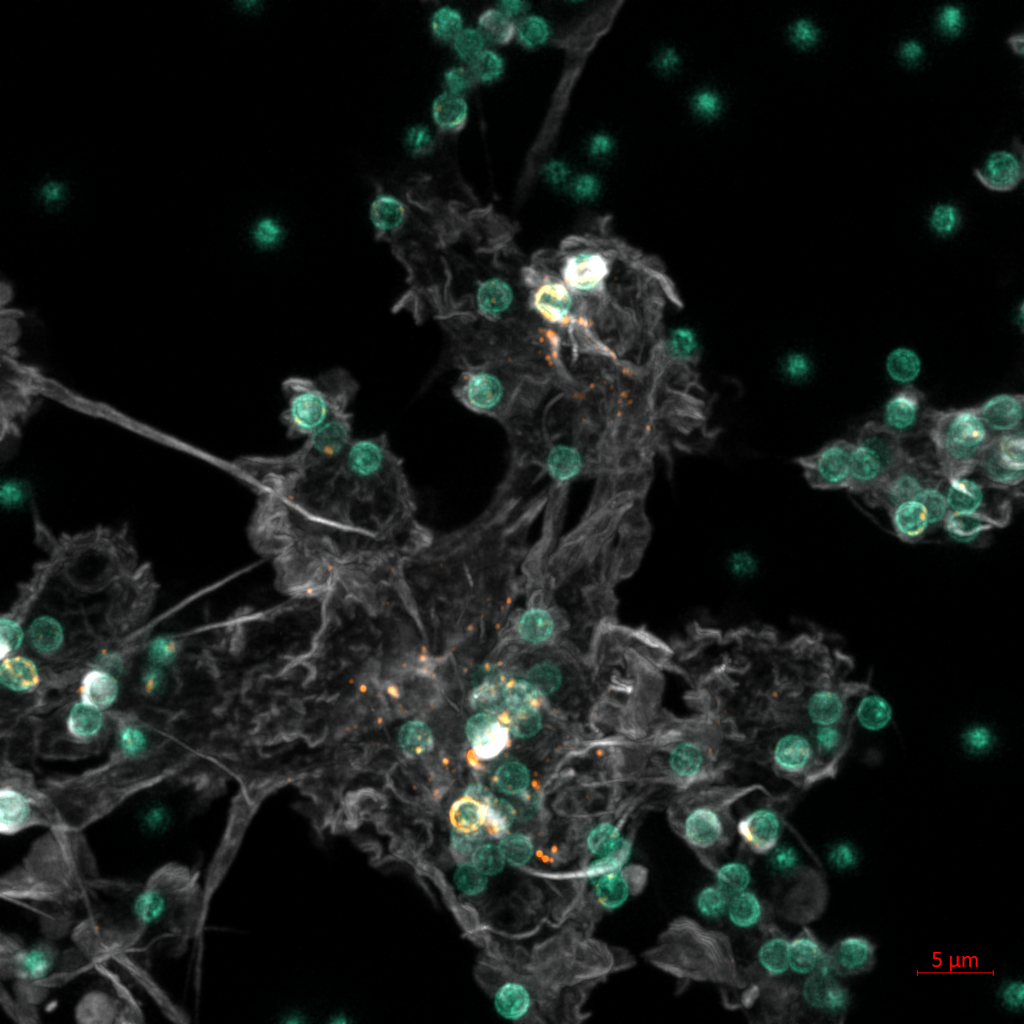

Supplement: Supplementary file 5 — Source data Fig. 4 [file 44318_2025_620_MOESM5_ESM.zip › Figure 4/4E/PSHIP1 I6G ruler_c1+2+3.tif]

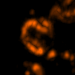

Supplement: Supplementary file 5 — Source data Fig. 4 [file 44318_2025_620_MOESM5_ESM.zip › Figure 4/4E/PSHIP1 I6G_c1 copy 2.tif]

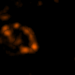

Supplement: Supplementary file 5 — Source data Fig. 4 [file 44318_2025_620_MOESM5_ESM.zip › Figure 4/4E/PSHIP1 I6G_c1 copy 3.tif]

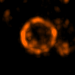

Supplement: Supplementary file 5 — Source data Fig. 4 [file 44318_2025_620_MOESM5_ESM.zip › Figure 4/4E/PSHIP1 I6G_c1 copy.tif]

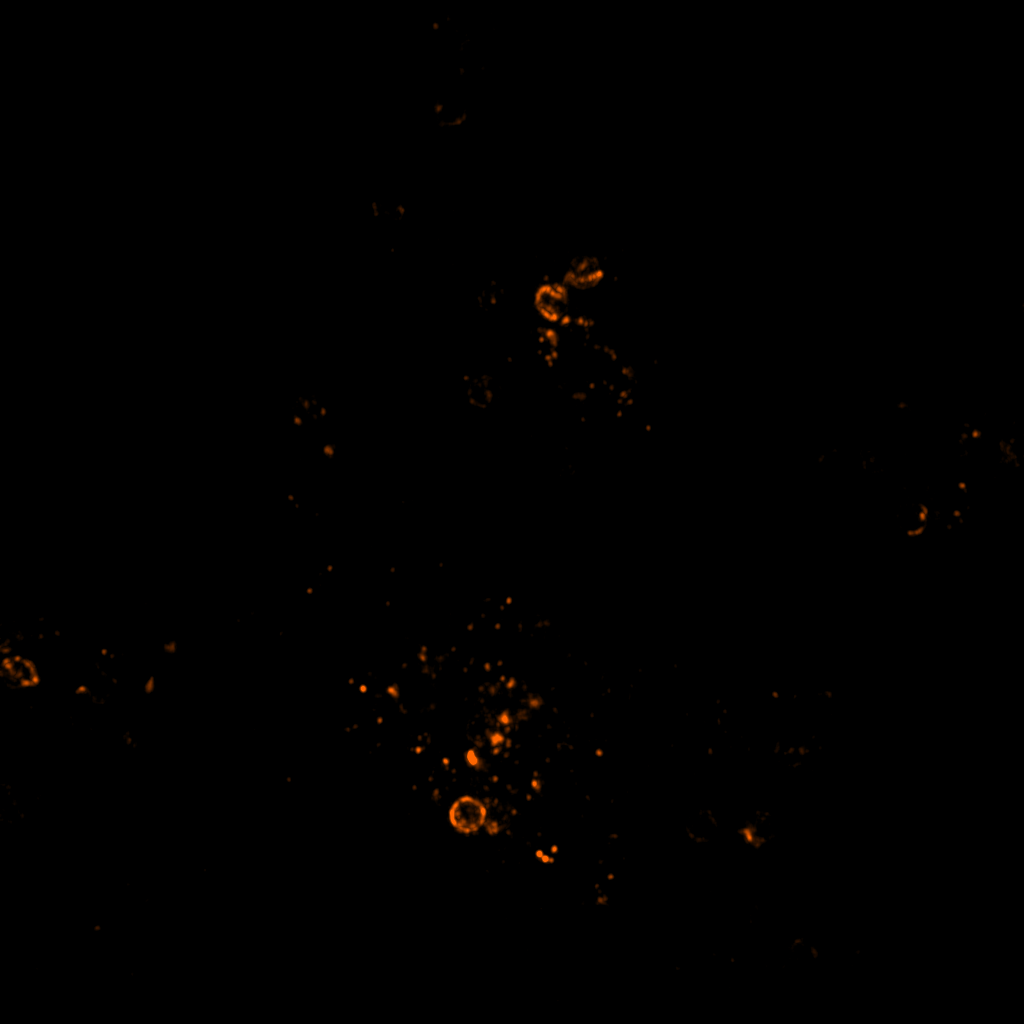

Supplement: Supplementary file 5 — Source data Fig. 4 [file 44318_2025_620_MOESM5_ESM.zip › Figure 4/4E/PSHIP1 I6G_c1.tif]

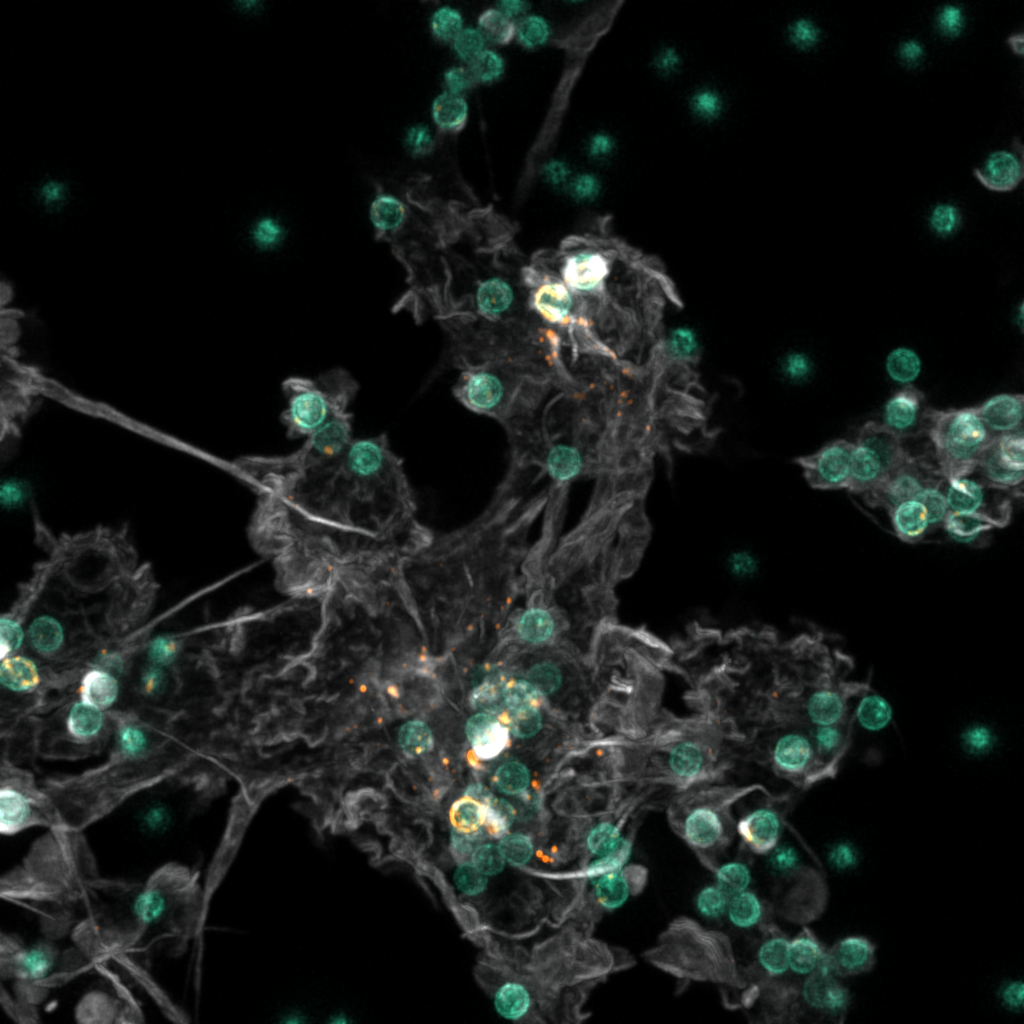

Supplement: Supplementary file 5 — Source data Fig. 4 [file 44318_2025_620_MOESM5_ESM.zip › Figure 4/4E/PSHIP1 I6G_c1+2+3.tif]
