# Supplementary figures and images for: DNGR-1 signalling limits dendritic cell activation for optimal antigen cross-presentation (part 2 of 2)
Source: EMBO J. 2025 Oct 29;44(23):6857–91. doi: 10.1038/s44318-025-00620-z (PMC12669754; doi:10.1038/s44318-025-00620-z)

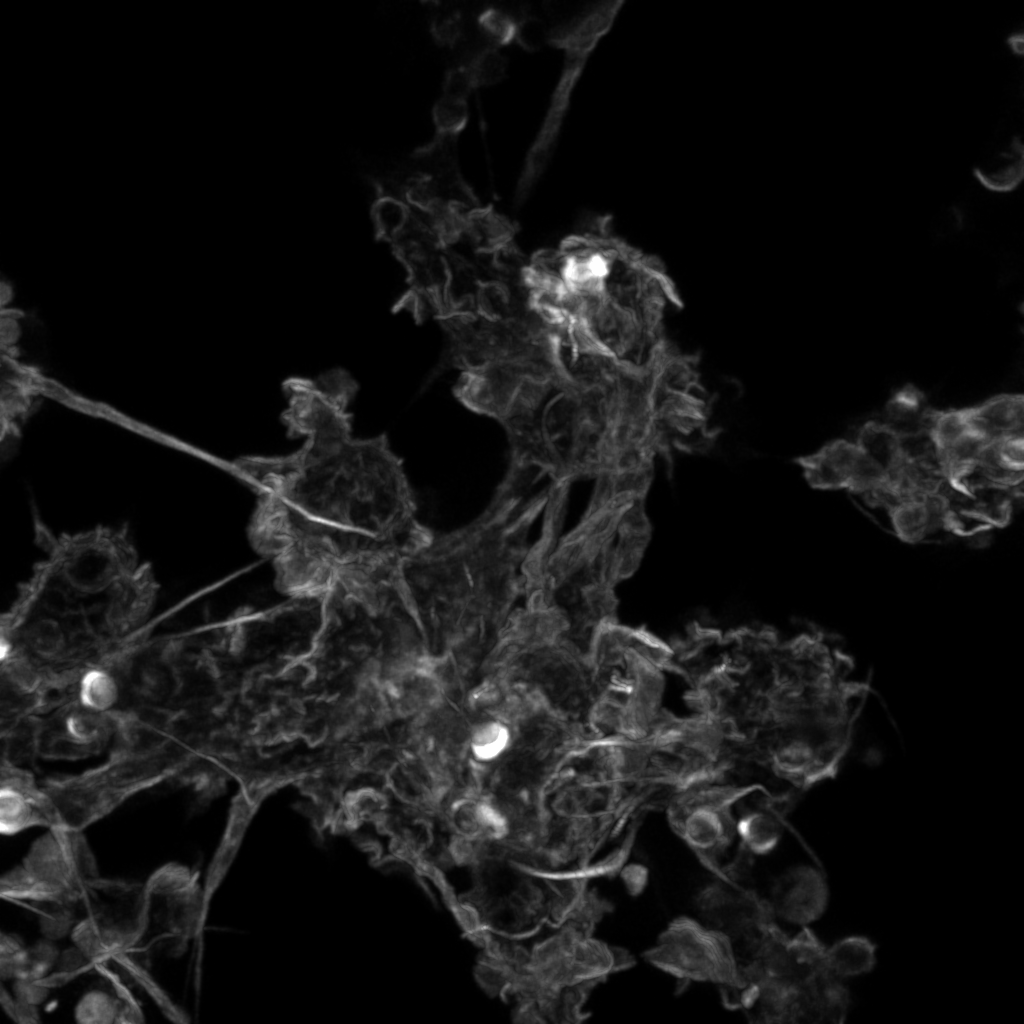

Supplement: Supplementary file 5 — Source data Fig. 4 [file 44318_2025_620_MOESM5_ESM.zip › Figure 4/4E/PSHIP1 I6G_c2.tif]

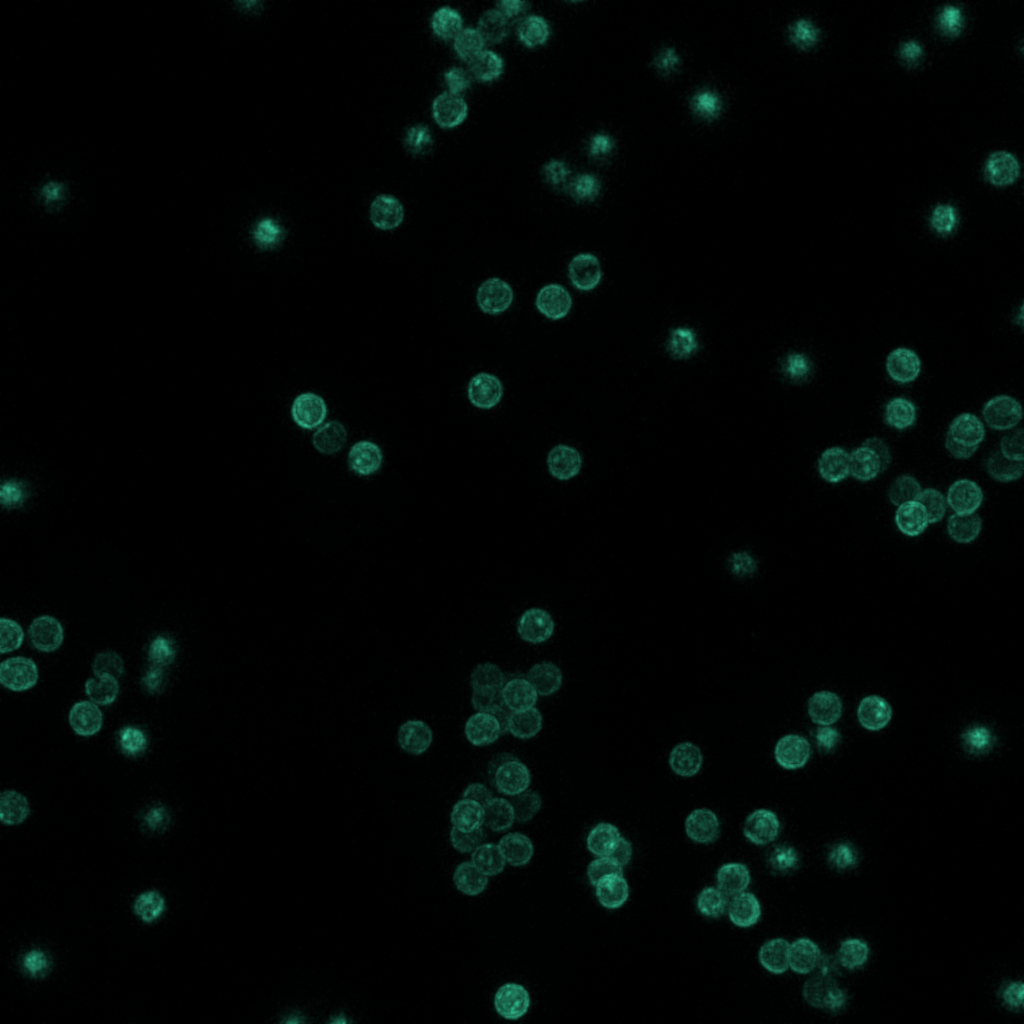

Supplement: Supplementary file 5 — Source data Fig. 4 [file 44318_2025_620_MOESM5_ESM.zip › Figure 4/4E/PSHIP1 I6G_c3.tif]

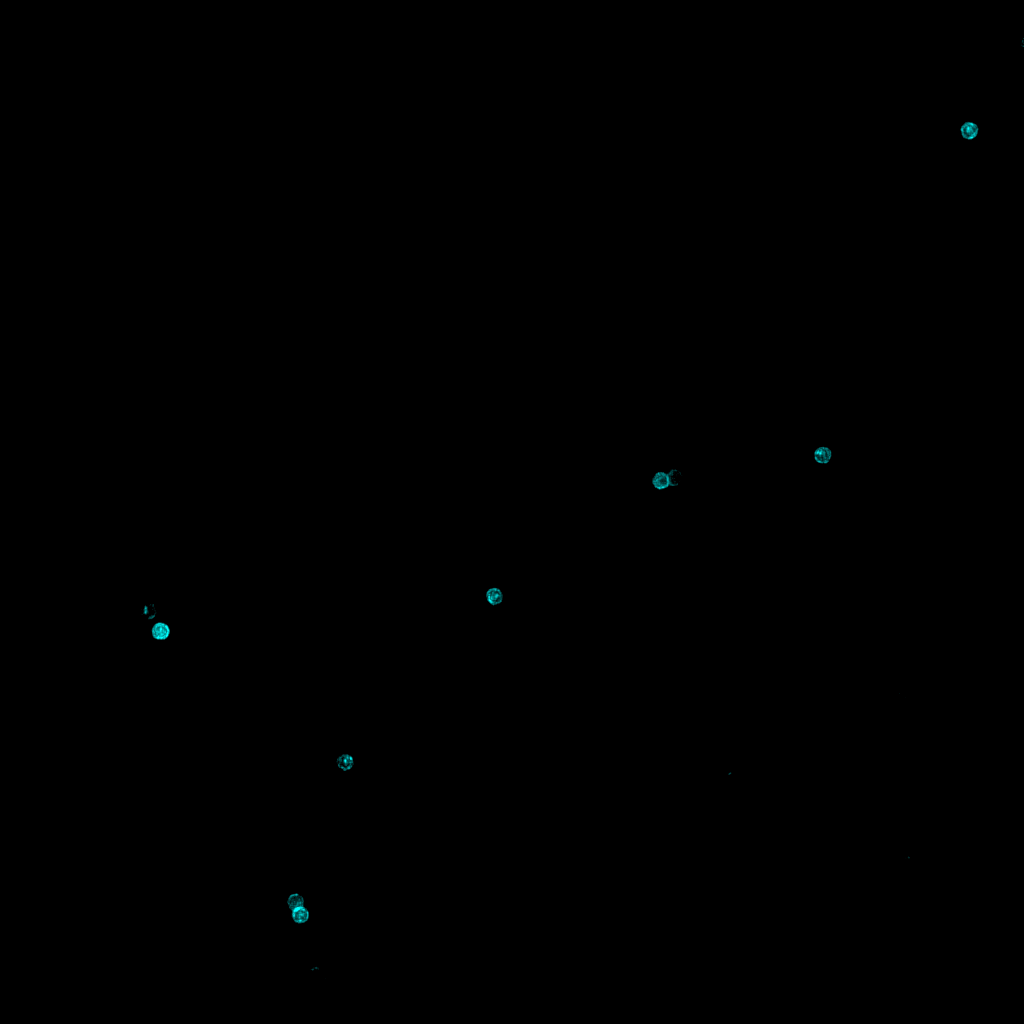

Supplement: Supplementary file 6 — Source data Fig. 5 [file 44318_2025_620_MOESM6_ESM.zip › Figure 5/5H/C9 Wide_c2.tif]

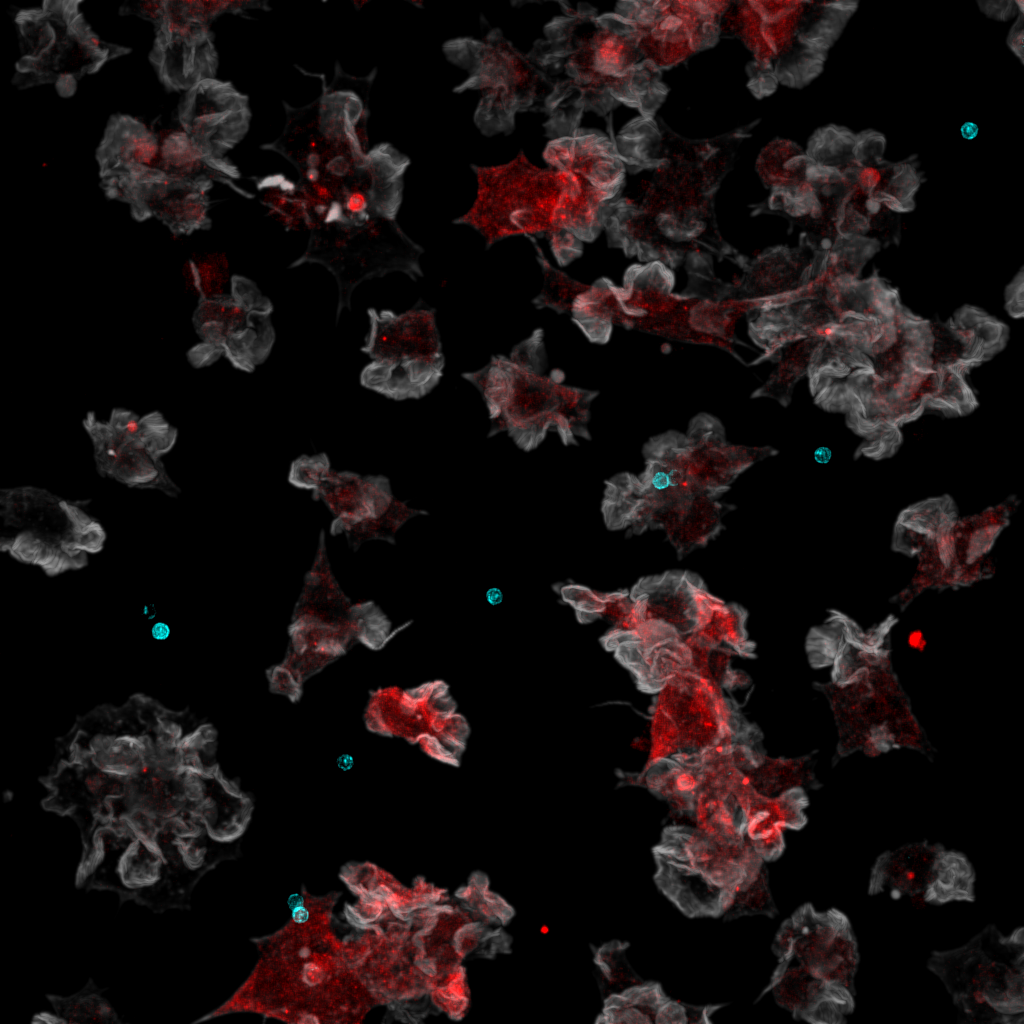

Supplement: Supplementary file 6 — Source data Fig. 5 [file 44318_2025_620_MOESM6_ESM.zip › Figure 5/5H/C9 Wide_c2+4+5.tif]

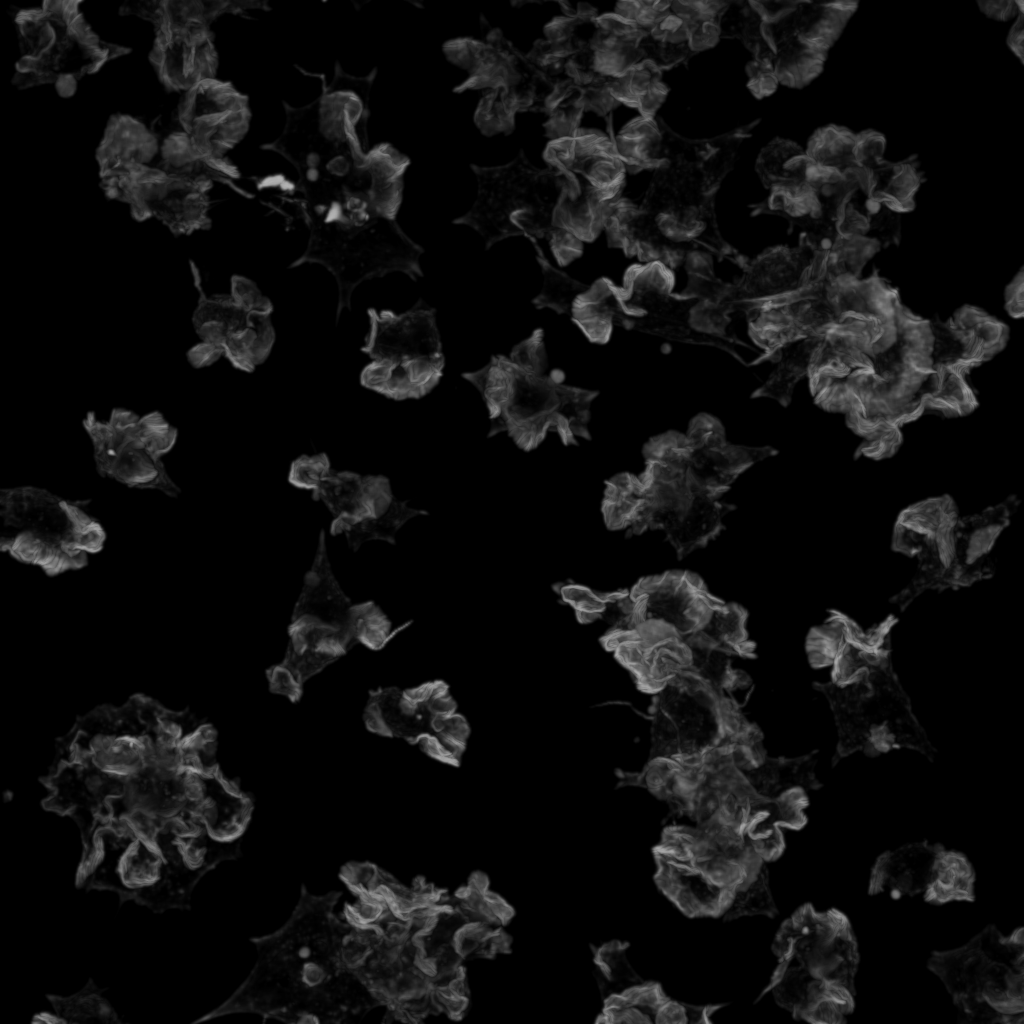

Supplement: Supplementary file 6 — Source data Fig. 5 [file 44318_2025_620_MOESM6_ESM.zip › Figure 5/5H/C9 Wide_c4.tif]

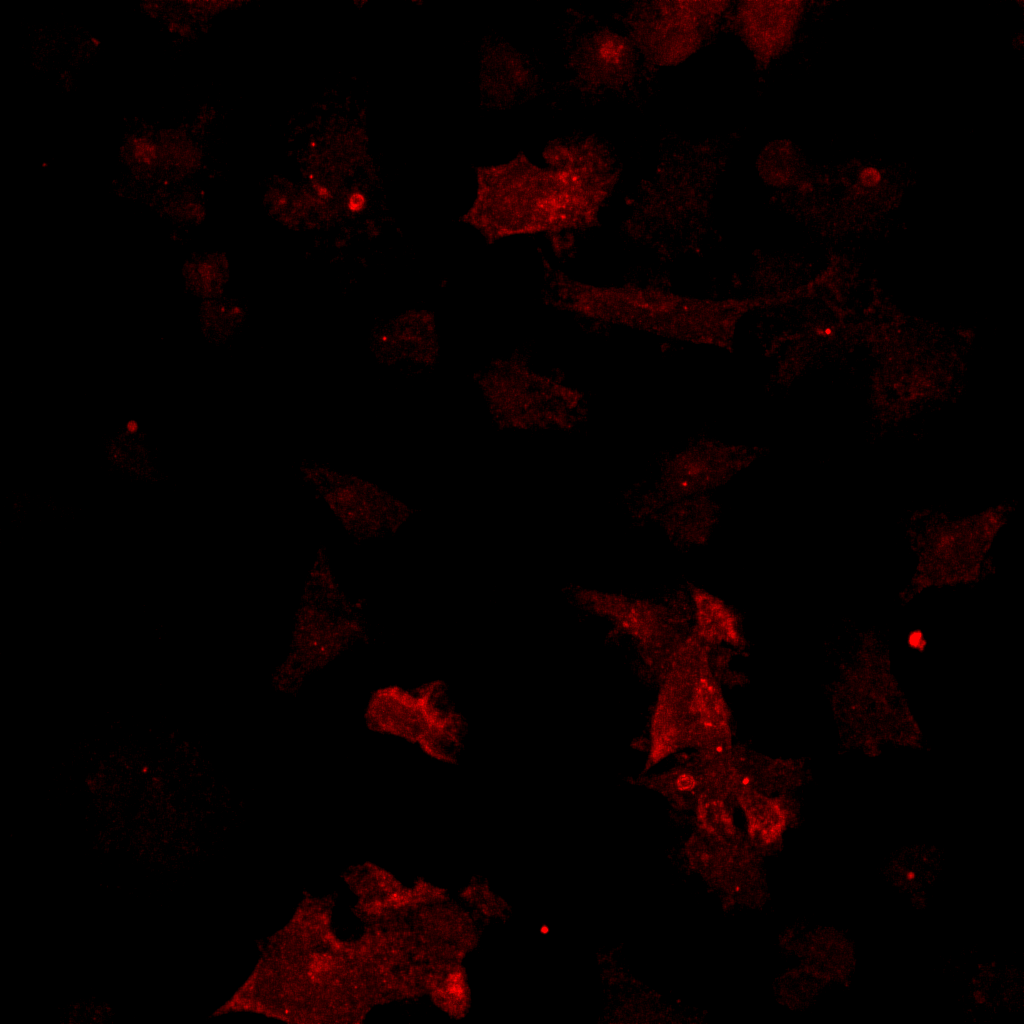

Supplement: Supplementary file 6 — Source data Fig. 5 [file 44318_2025_620_MOESM6_ESM.zip › Figure 5/5H/C9 Wide_c5.tif]

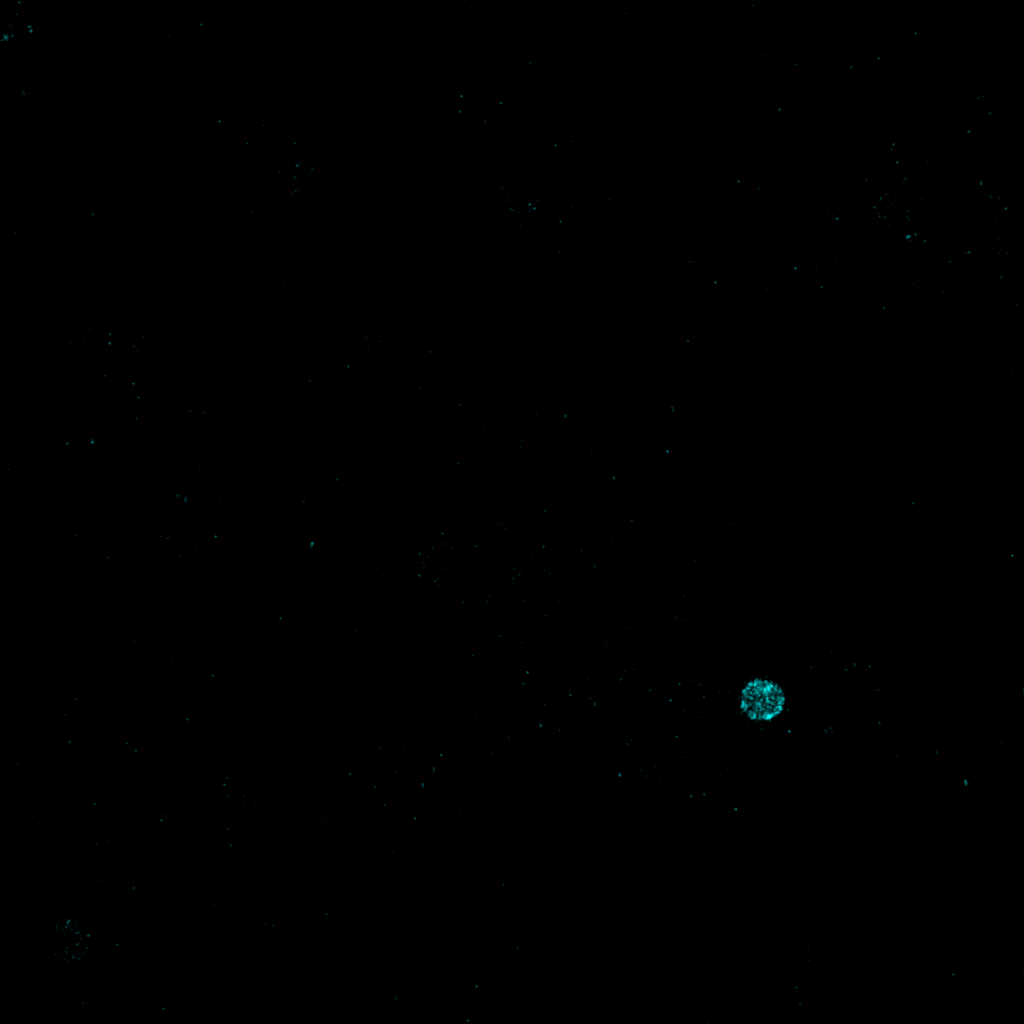

Supplement: Supplementary file 6 — Source data Fig. 5 [file 44318_2025_620_MOESM6_ESM.zip › Figure 5/5H/C9 Zoom_c2.tif]

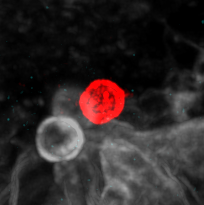

Supplement: Supplementary file 6 — Source data Fig. 5 [file 44318_2025_620_MOESM6_ESM.zip › Figure 5/5H/C9 Zoom_c2+4+5 copy.tif]

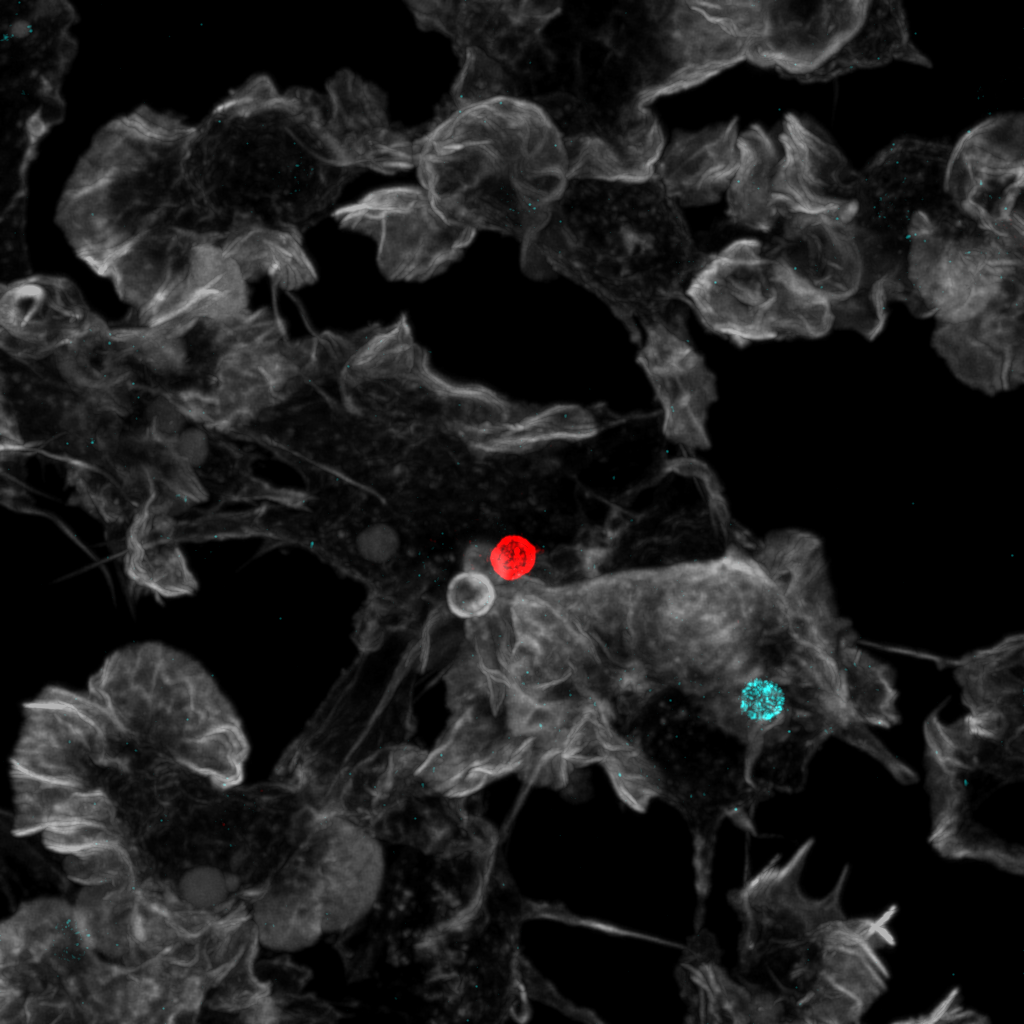

Supplement: Supplementary file 6 — Source data Fig. 5 [file 44318_2025_620_MOESM6_ESM.zip › Figure 5/5H/C9 Zoom_c2+4+5.tif]

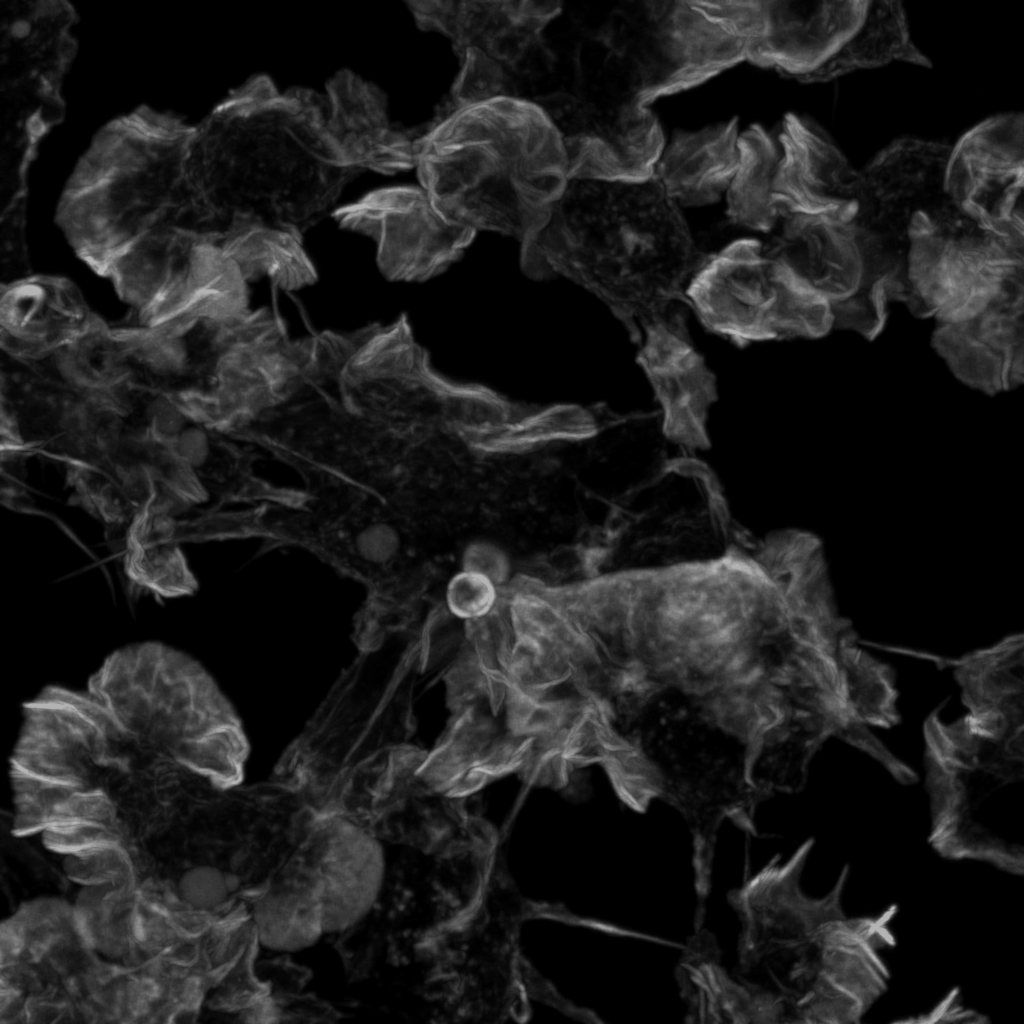

Supplement: Supplementary file 6 — Source data Fig. 5 [file 44318_2025_620_MOESM6_ESM.zip › Figure 5/5H/C9 Zoom_c4.tif]

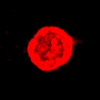

Supplement: Supplementary file 6 — Source data Fig. 5 [file 44318_2025_620_MOESM6_ESM.zip › Figure 5/5H/C9 Zoom_c5 copy.tif]

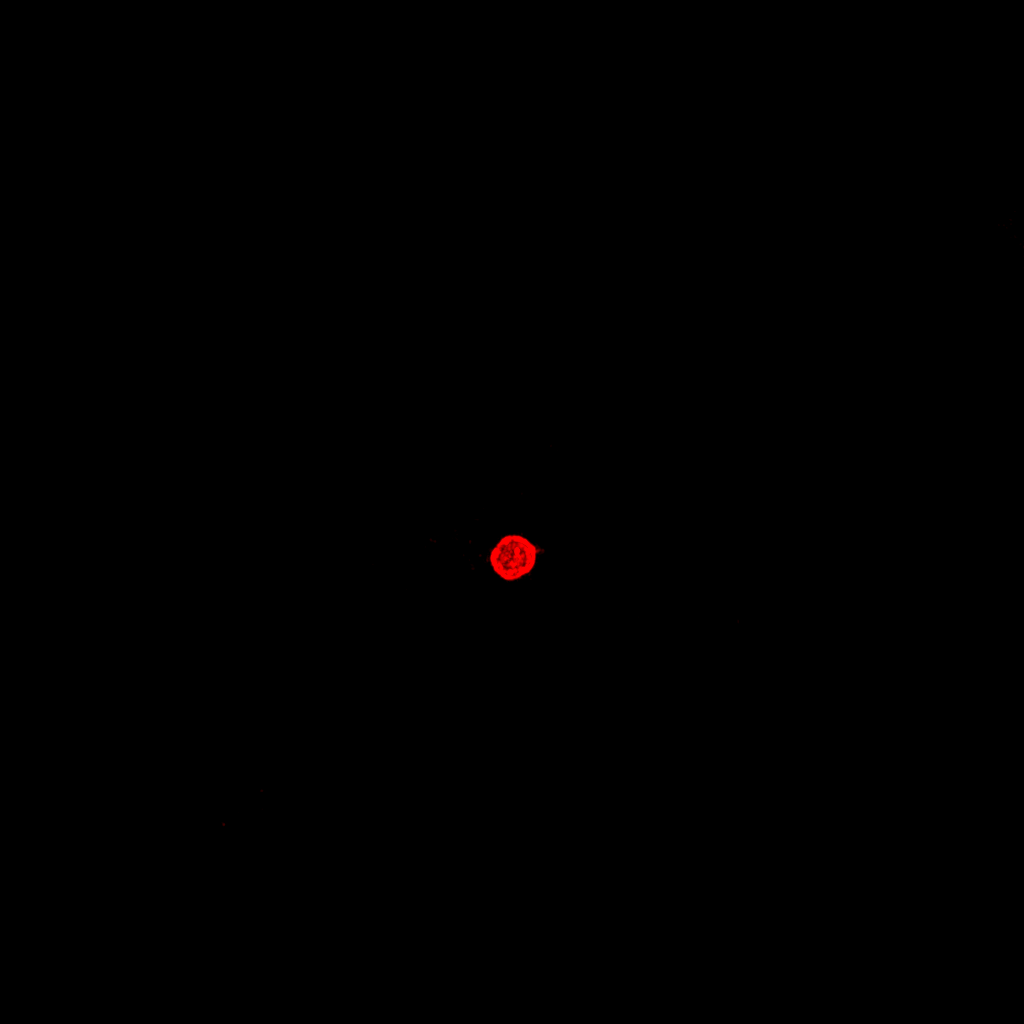

Supplement: Supplementary file 6 — Source data Fig. 5 [file 44318_2025_620_MOESM6_ESM.zip › Figure 5/5H/C9 Zoom_c5.tif]

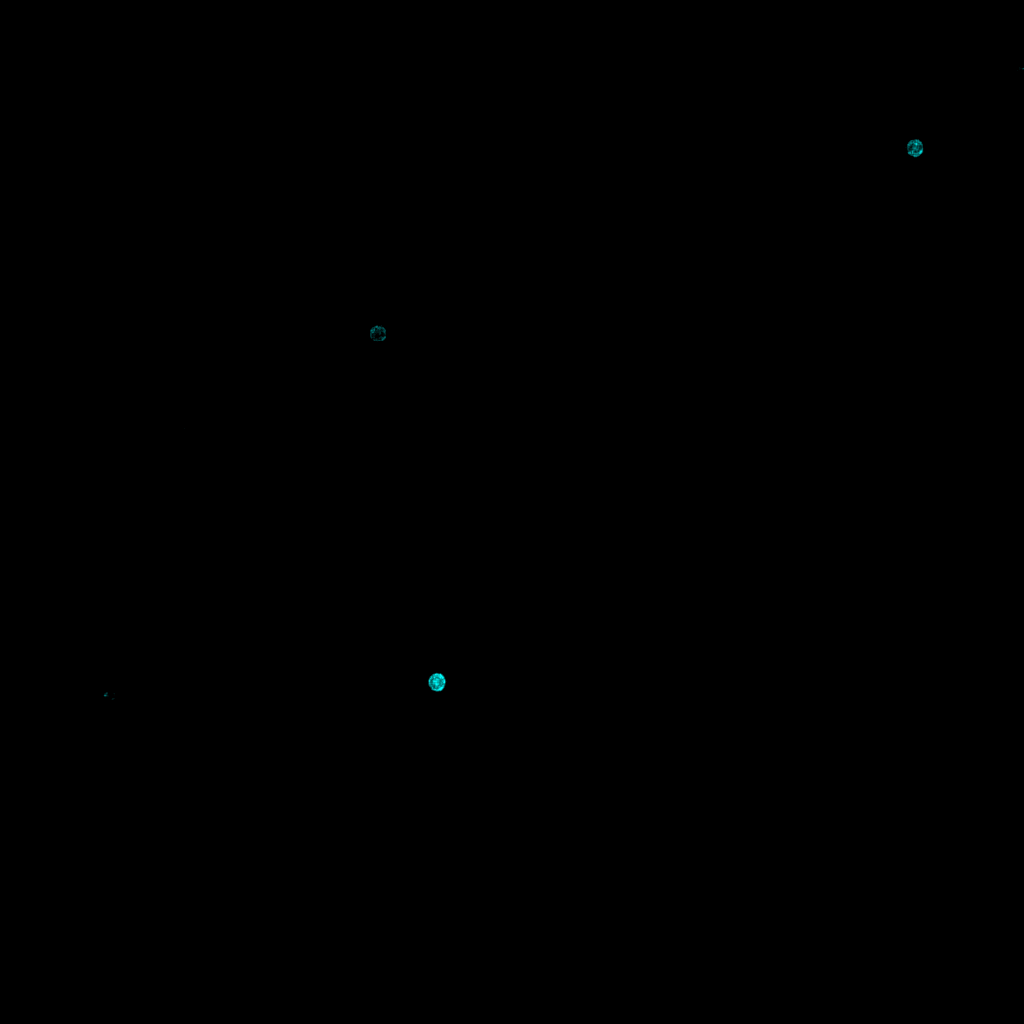

Supplement: Supplementary file 6 — Source data Fig. 5 [file 44318_2025_620_MOESM6_ESM.zip › Figure 5/5H/I6G Wide_c2.tif]

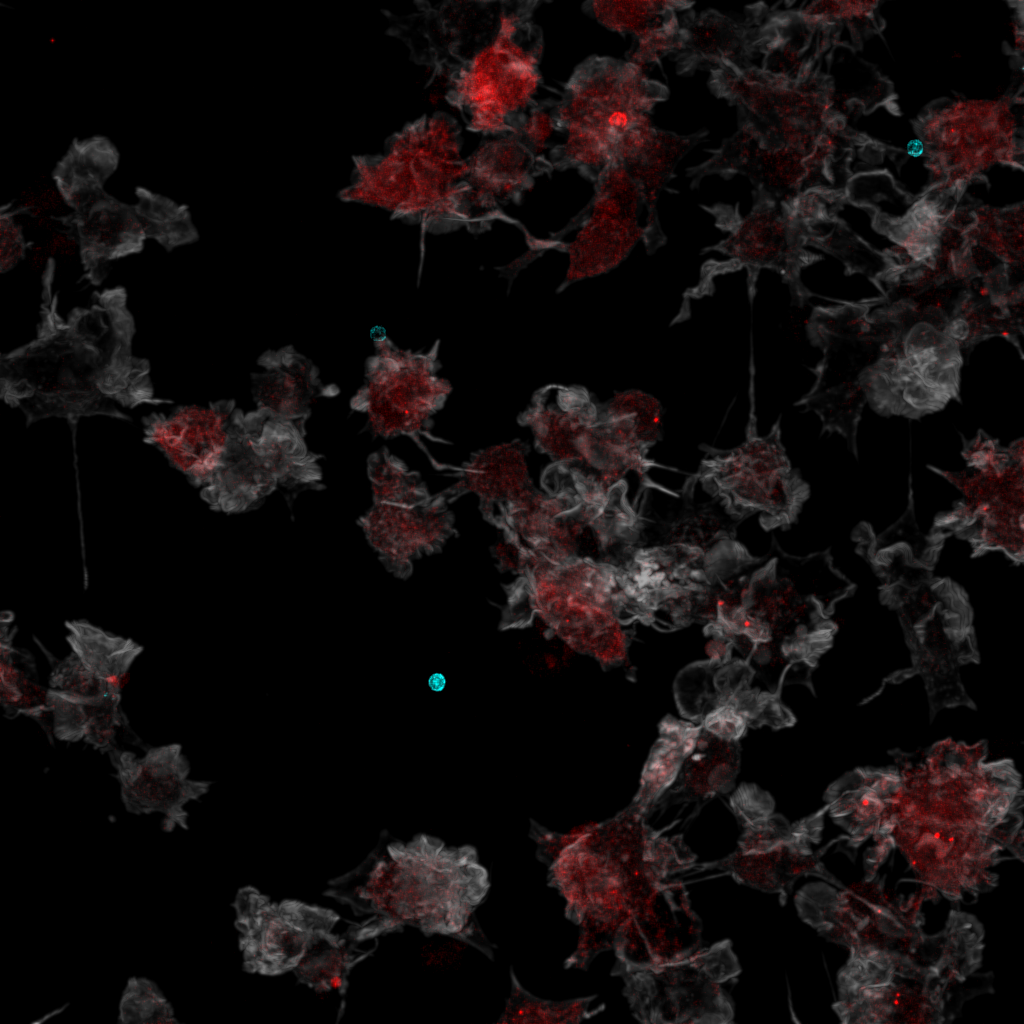

Supplement: Supplementary file 6 — Source data Fig. 5 [file 44318_2025_620_MOESM6_ESM.zip › Figure 5/5H/I6G Wide_c2+4+5.tif]

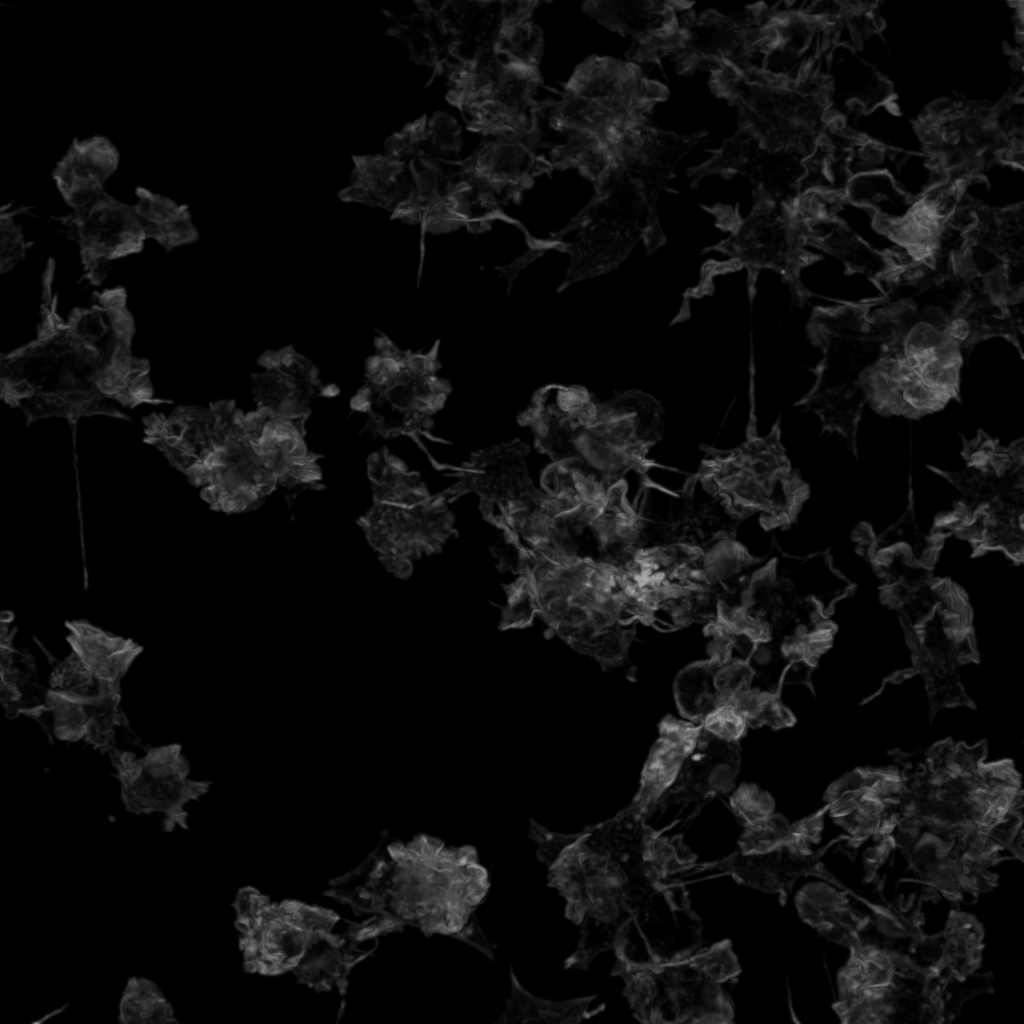

Supplement: Supplementary file 6 — Source data Fig. 5 [file 44318_2025_620_MOESM6_ESM.zip › Figure 5/5H/I6G Wide_c4.tif]

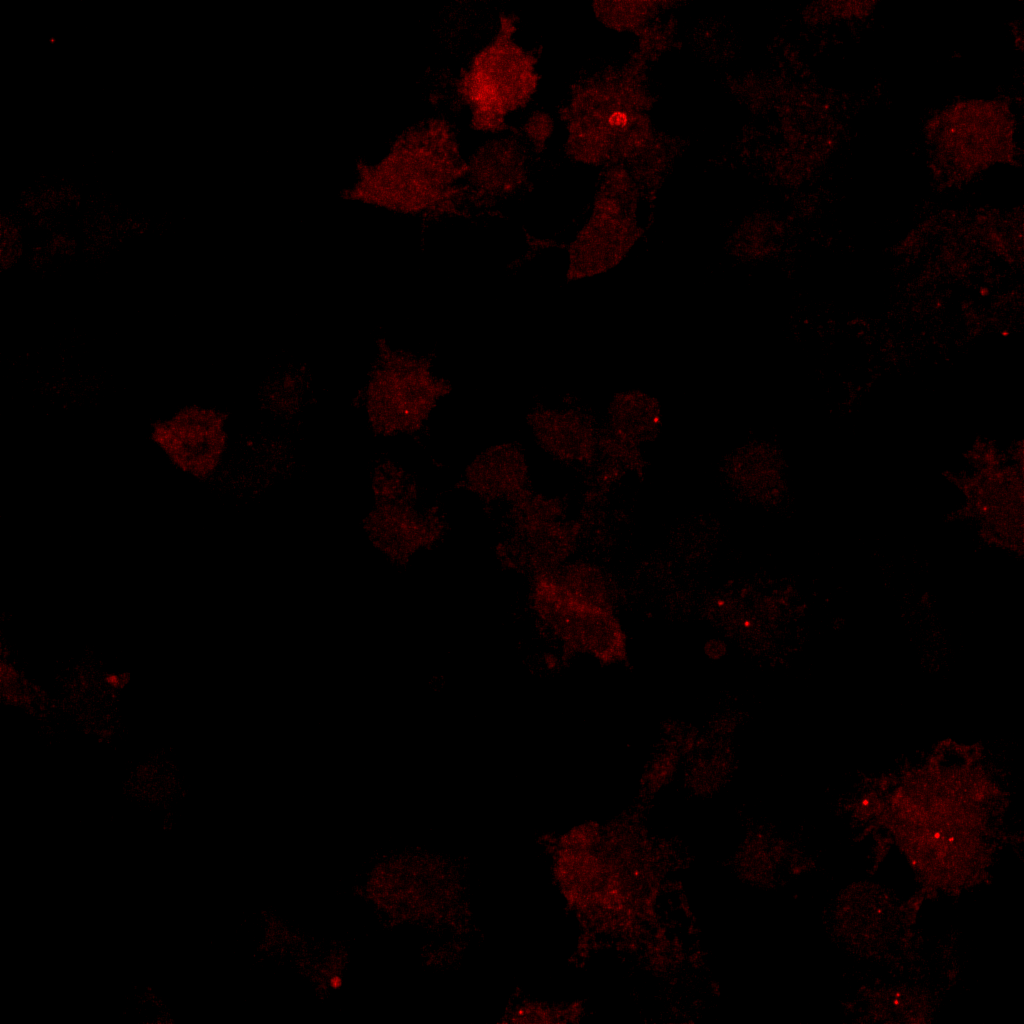

Supplement: Supplementary file 6 — Source data Fig. 5 [file 44318_2025_620_MOESM6_ESM.zip › Figure 5/5H/I6G Wide_c5.tif]

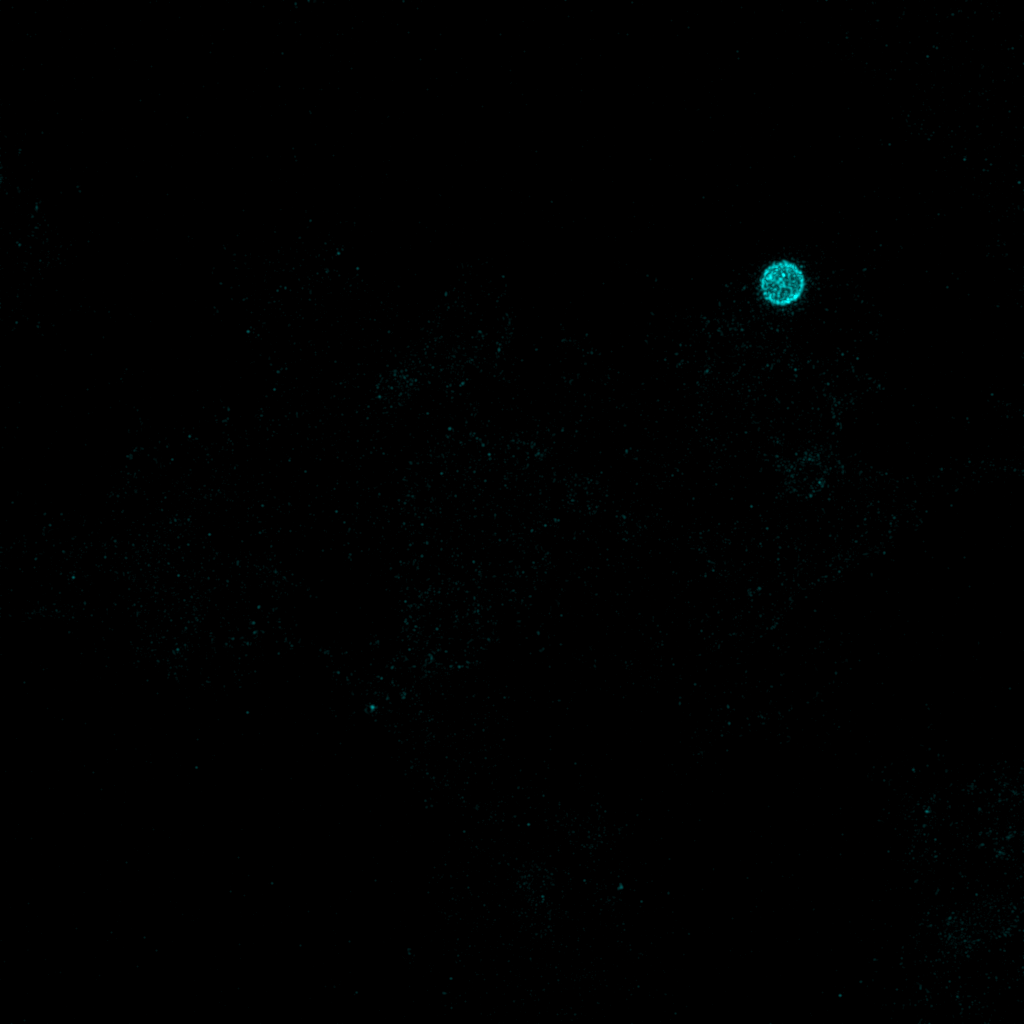

Supplement: Supplementary file 6 — Source data Fig. 5 [file 44318_2025_620_MOESM6_ESM.zip › Figure 5/5H/I6G Zoom_c2.tif]

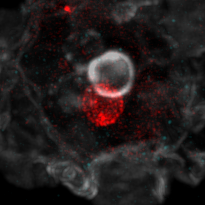

Supplement: Supplementary file 6 — Source data Fig. 5 [file 44318_2025_620_MOESM6_ESM.zip › Figure 5/5H/I6G Zoom_c2+4+5 copy.tif]

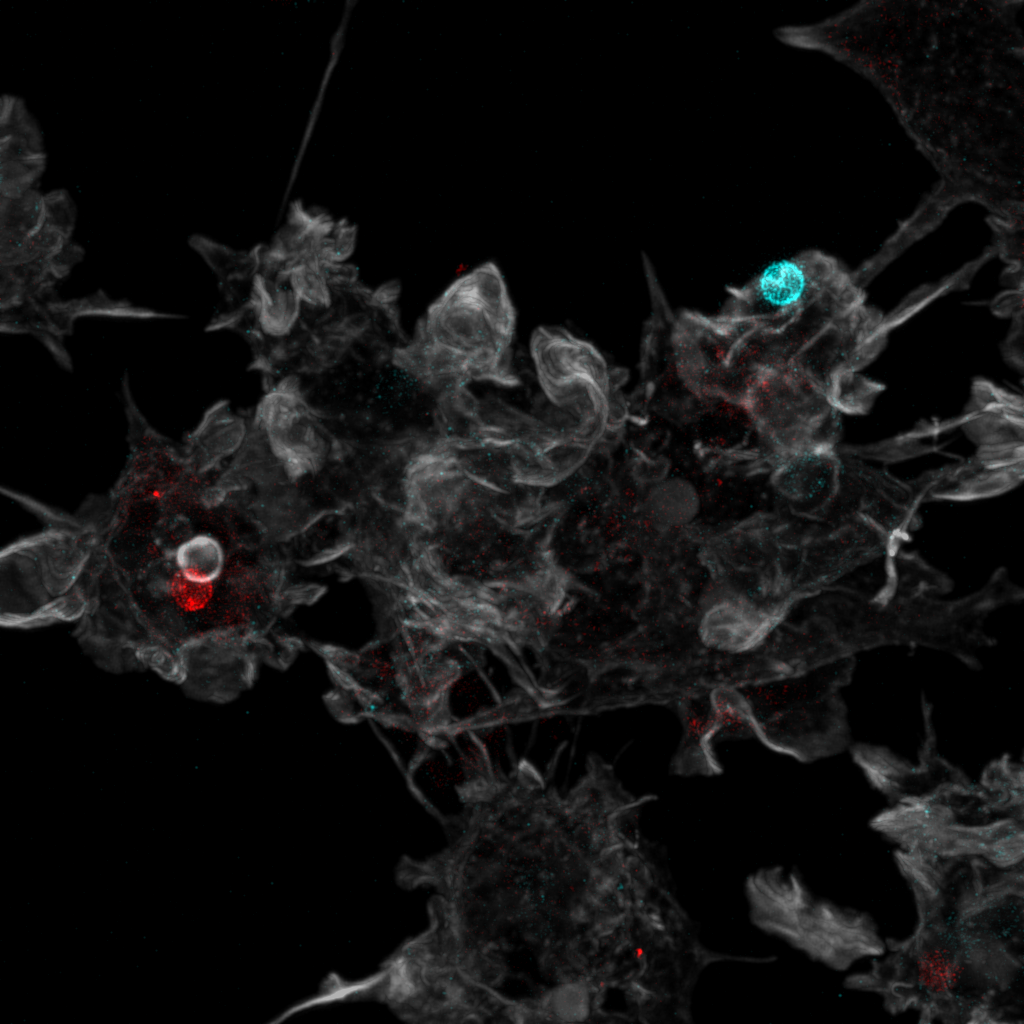

Supplement: Supplementary file 6 — Source data Fig. 5 [file 44318_2025_620_MOESM6_ESM.zip › Figure 5/5H/I6G Zoom_c2+4+5.tif]

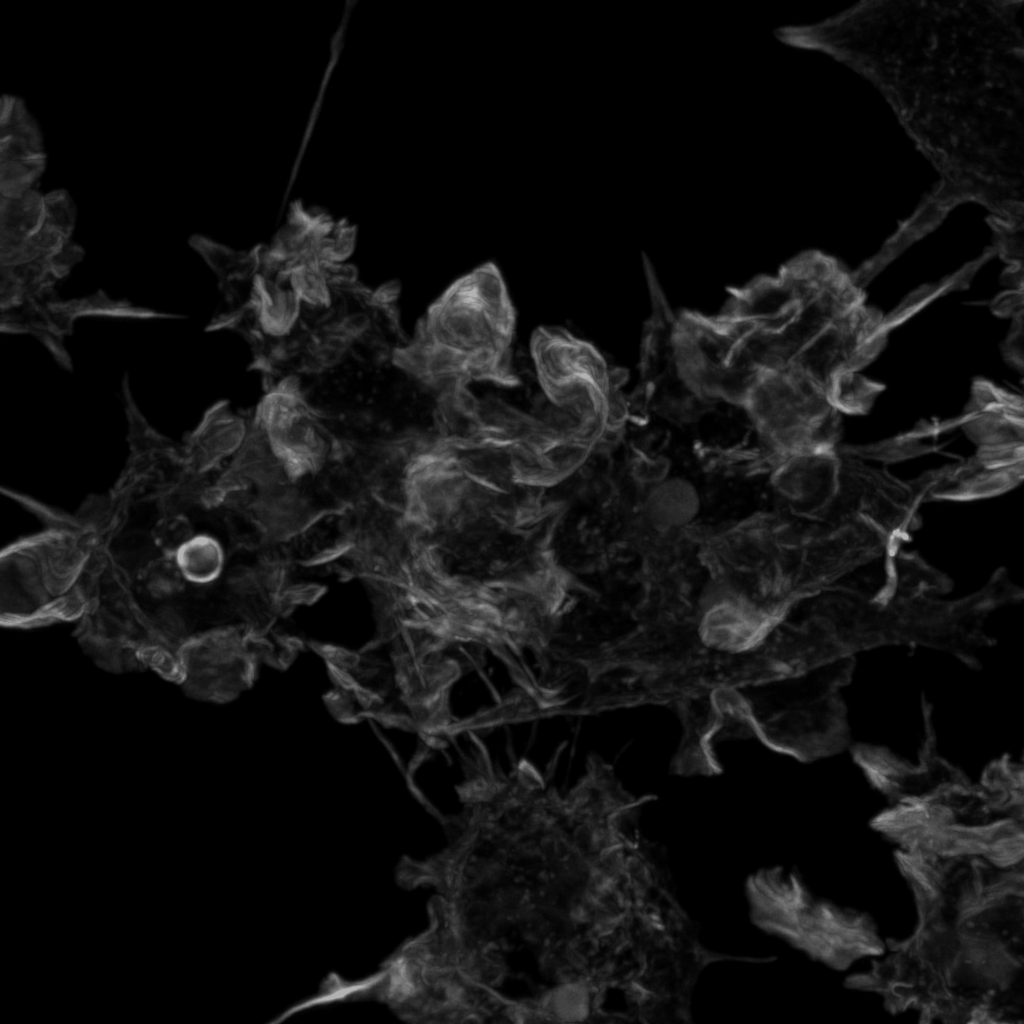

Supplement: Supplementary file 6 — Source data Fig. 5 [file 44318_2025_620_MOESM6_ESM.zip › Figure 5/5H/I6G Zoom_c4.tif]

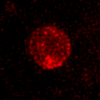

Supplement: Supplementary file 6 — Source data Fig. 5 [file 44318_2025_620_MOESM6_ESM.zip › Figure 5/5H/I6G Zoom_c5 copy.tif]

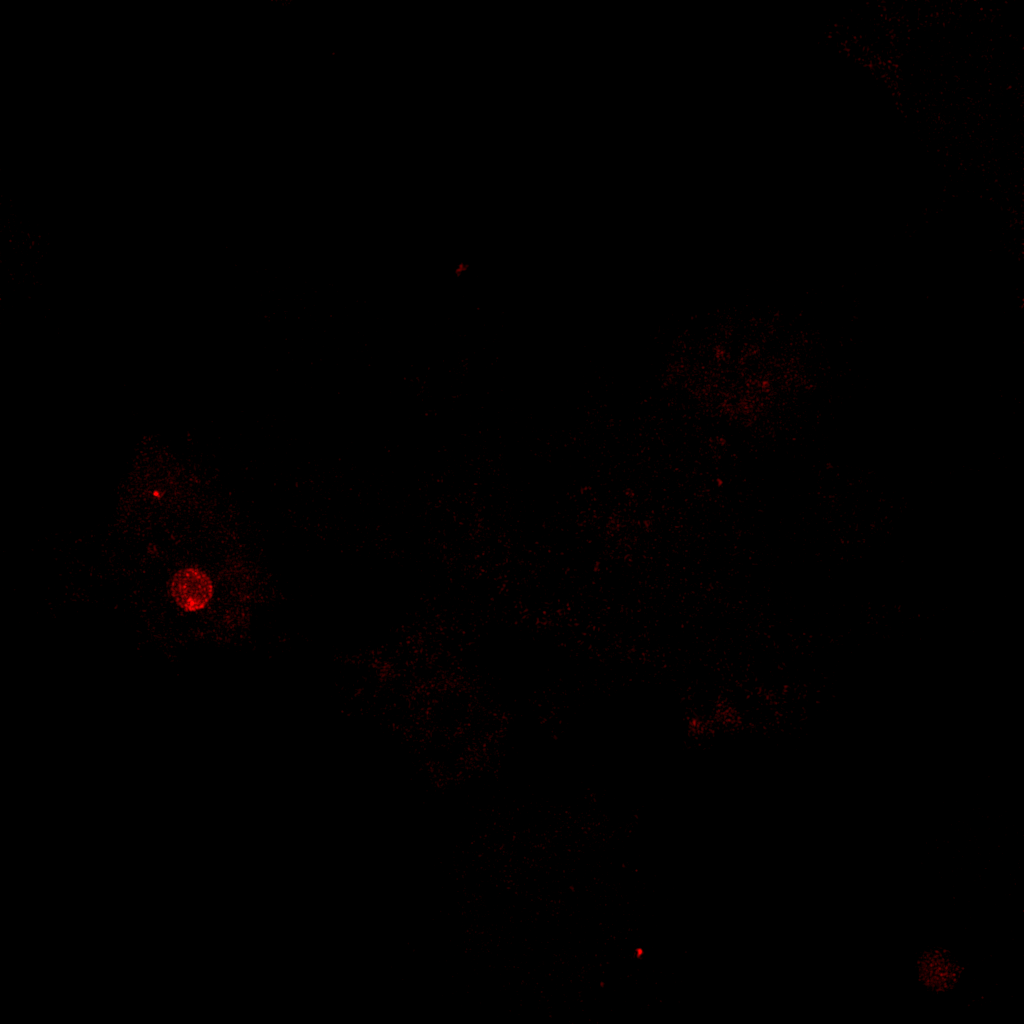

Supplement: Supplementary file 6 — Source data Fig. 5 [file 44318_2025_620_MOESM6_ESM.zip › Figure 5/5H/I6G Zoom_c5.tif]
