# Supplementary material for: DNGR-1 signalling limits dendritic cell activation for optimal antigen cross-presentation
Source: EMBO J. 2025 Oct 29;44(23):6857–91. doi: 10.1038/s44318-025-00620-z (PMC12669754; doi:10.1038/s44318-025-00620-z)
Supplement: Supplementary file 8 — Expanded View Figures [file 44318_2025_620_MOESM8_ESM.pdf]

Expanded View Figures

Figure EV1. Assessment of DNGR-1 stimulation on cDC1 activation (related to Fig. 1).

(**A**) Representative flow cytometry gating strategy for analyzing cDCs derived from bone marrow-FLT3L cultures (BM-FLT3L) before and after $XCR1^{+}$ MACS enrichment. (**B**) DNGR-1 expression by WT BM-FLT3L cDC1 and cDC2 compared to $C9^{KI\text{-}Cre}$ cDC1. (**C–E**) WT, $C9^{KI\text{-}Cre}$ BM-FLT3L cDC1s or (**F**, **G**) C9 KO MuTuDCs or those reconstituted with indicated receptors were cultured overnight ± designated stimuli and assessed for surface expression of the specified markers by flow cytometry. (**C**) $H2\text{-}K^{b}$ expression from biological duplicates combined from two experiments with mean ± SEM plotted (left) and representative histograms (right). (**D**) Representative histograms of staining for indicated markers. (**E**) Cells were cultured overnight on uncoated plates or plates coated with different concentrations of α-DNGR-1 IgG (clone 1F6 or 7H11, as indicated) and assessed for indicated surface marker expression by flow cytometry. MFI values from biological duplicates pooled from two independent experiments with mean ± SEM is plotted. (**F**) Representative histograms of staining for indicated markers. (**G**) $H2\text{-}K^{b}$ expression from biological duplicates pooled from two independent experiments with mean ± SEM is plotted. Data are representative of two (**A–G**) independent experiments. (**C**, **E**, **G**) Data were analysed using Tukey-corrected two-way ANOVA with significant values comparing against untreated samples plotted. (**C**) ****$P < 0.0001$.

© The Author(s)

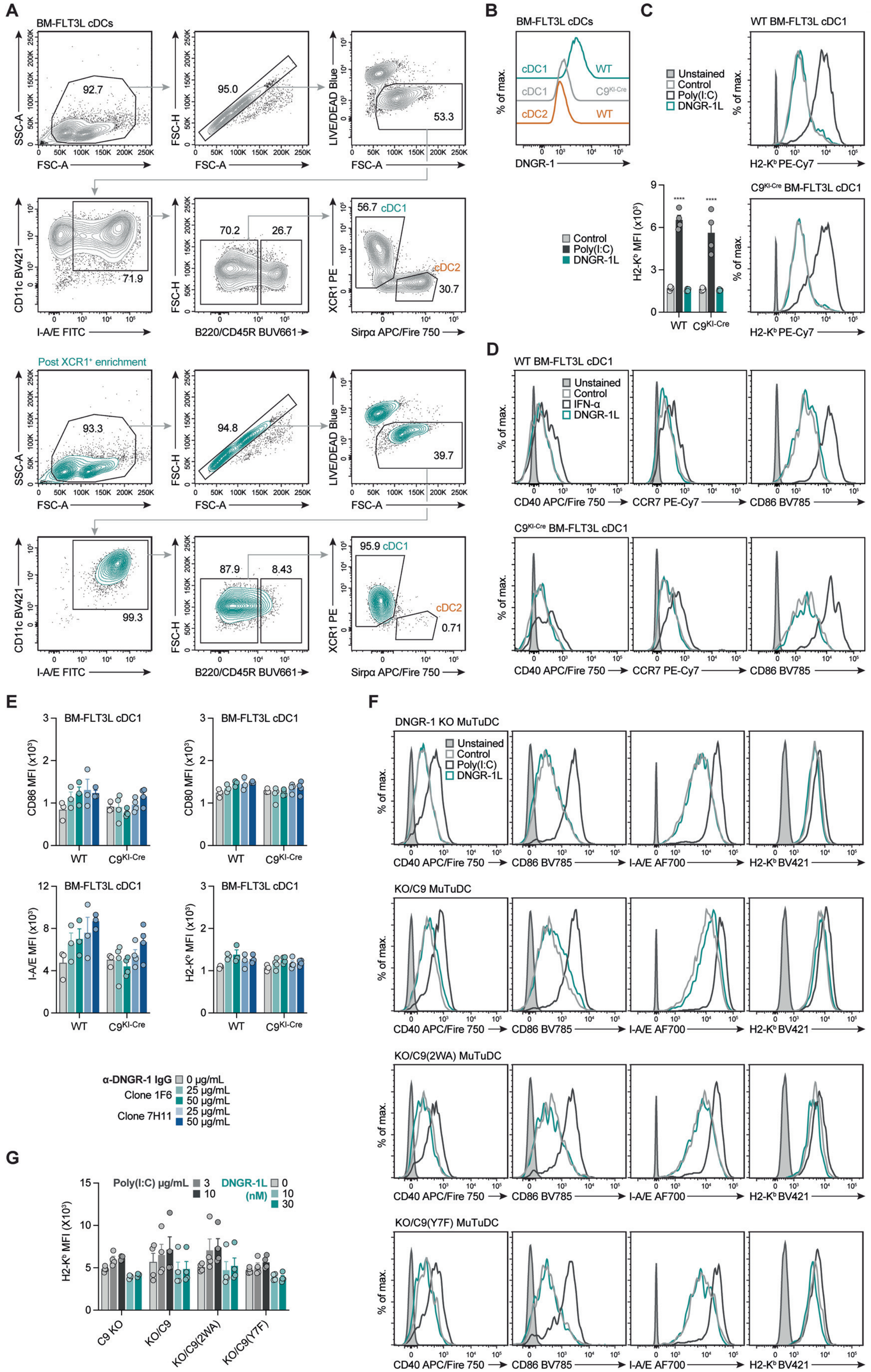
A
BM-FLT3L cDCs
Post XCR1⁺ enrichment
SSC-A
FSC-A
FSC-H
LIVE/DEAD Blue
CD11c BV421
I-A/E FITC
B220/CD45R BUV661
XCR1 PE
Sirpα APC/Fire 750
cDC1
cDC2
B
BM-FLT3L cDCs
DNGR-1
% of max.
C
WT BM-FLT3L cDC1
C9KI-Cre BM-FLT3L cDC1
Unstained
Control
Poly(I:C)
DNGR-1L
H2-Kb PE-Cy7
H2-Kb MFI (x10³)
WT
D
WT BM-FLT3L cDC1
C9KI-Cre BM-FLT3L cDC1
IFN-α
CD40 APC/Fire 750
CCR7 PE-Cy7
CD86 BV785
E
BM-FLT3L cDC1
CD86 MFI (x10³)
CD80 MFI (x10³)
I-A/E MFI (x10³)
H2-Kb MFI (x10³)
α-DNGR-1 IgG
Clone 1F6
Clone 7H11
0 μg/mL
25 μg/mL
50 μg/mL
F
DNGR-1 KO MuTuDC
KO/C9 MuTuDC
KO/C9(2WA) MuTuDC
KO/C9(Y7F) MuTuDC
I-A/E AF700
H2-Kb BV421
G
Poly(I:C) μg/mL
DNGR-1L (nM)
H2-Kb MFI (X10³)
C9 KO
KO/C9
KO/C9(2WA)
KO/C9(Y7F)

 © The Author(s)

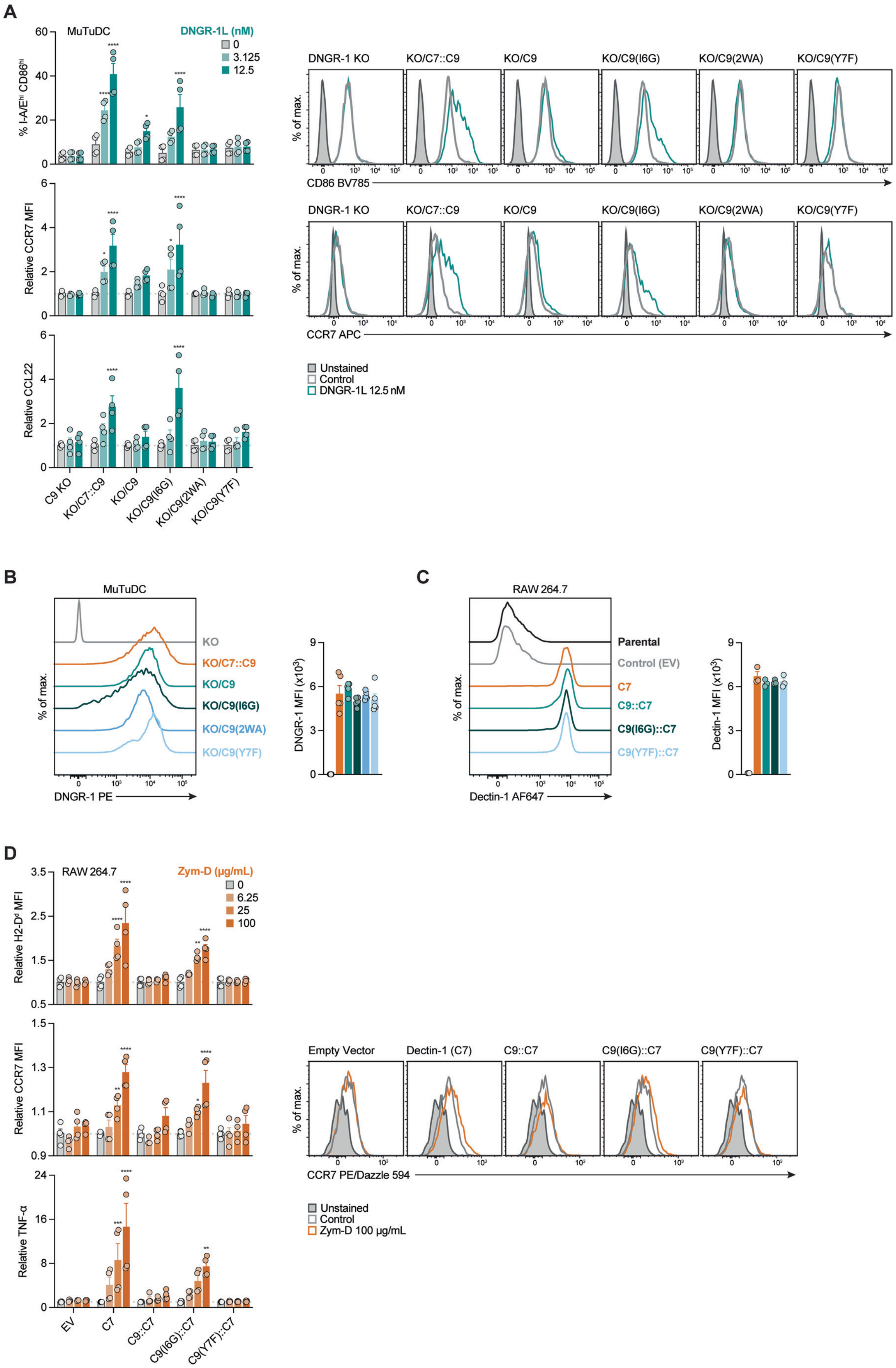
A
MuTuDC
DNGR-1L (nM)
0
3.125
12.5
% I-A/E^hi CD86^hi
Relative CCR7 MFI
Relative CCL22
C9 KO
KO/C7::C9
KO/C9
KO/C9(I6G)
KO/C9(2WA)
KO/C9(Y7F)
DNGR-1 KO
% of max.
CD86 BV785
CCR7 APC
Unstained
Control
DNGR-1L 12.5 nM
B
MuTuDC
KO
KO/C7::C9
KO/C9
KO/C9(I6G)
KO/C9(2WA)
KO/C9(Y7F)
DNGR-1 PE
DNGR-1 MFI (x10^3)
C
RAW 264.7
Parental
Control (EV)
C7
C9::C7
C9(I6G)::C7
C9(Y7F)::C7
Dectin-1 AF647
Dectin-1 MFI (x10^3)
D
RAW 264.7
Zym-D (μg/mL)
0
6.25
25
100
Relative H2-D^d MFI
Relative CCR7 MFI
Relative TNF-α
EV
C7
C9::C7
C9(I6G)::C7
C9(Y7F)::C7
Empty Vector
Dectin-1 (C7)
CCR7 PE/Dazzle 594
Unstained
Control
Zym-D 100 μg/mL

◀ **Figure EV2. Cell lines to analyse DNGR-1 function (related to Fig. 2).**

(**A**) Analysis of the indicated surface marker expression (top, middle; flow cytometry) or CCL22 released into cultured supernatants (bottom; ELISA) from C9 KO MuTuDCs reconstituted or not with the indicated receptors and stimulated overnight ± DNGR-1L. Mean ± SEM from biological replicates pooled from two independent experiments (left) and representative flow cytometry profiles (right) are plotted. Data here are partly represented in Fig. 2D,E and are relative to average of untreated controls to better emphasise the response to DNGR-1L. Dotted line represents 1. (**B**) Flow cytometric analysis of surface DNGR-1 (C9) expression in C9 KO MuTuDCs or those reconstituted or not with the indicated receptors. Cell lines were established after sorting for equal expression of DNGR-1. (**C**) Flow cytometric analysis of surface Dectin-1 (C7) expression by parental RAW 264.7 cells or cells ectopically expressing the indicated receptors or transduced with empty vector (EV). Cell lines were established after sorting for equal expression of Dectin-1. (**B**, **C**) Representative histograms (left) and mean MFI ± SEM (right) are plotted from biological (**B**) quintuplets or (**C**) triplicates. (**D**) Analysis of the indicated surface marker expression (top, middle; flow cytometry) or TNF-α released into cultured supernatants (bottom; ELISA) from RAW 264.7 cells ectopically expressing the indicated receptors or transduced with EV and stimulated overnight ± Zym-D. Mean ± SEM from biological replicates pooled from two independent experiments (left) and representative flow cytometry profiles (right) are plotted. Data here are partly represented in Fig. 2F and are relative to average of untreated controls to better emphasise the response to Zym-D. Dotted line represents 1. Data are representative of two (**D**), or three (**A–C**) independent experiments. Data were analysed using Tukey-corrected two-way ANOVA with significant values comparing against untreated samples plotted (**A**, **D**). (**A**) I-A/E^{hi} $CD86^{hi}$ *$P = 0.0117$, ****$P < 0.0001$; CCR7 *$P = 0.0279$ (KO/C7::C9), $P = 0.0134$ (KO/C9(I6G)), ****$P < 0.0001$; CCL22 ****$P < 0.0001$, (**D**) H2-D^d **$P = 0.0011$, ****$P < 0.0001$; CCR7 *$P = 0.0319$, **$P = 0.0040$, ****$P < 0.0001$; TNF-α **$P = 0.0033$, ***$P = 0.0004$, ****$P < 0.0001$.

 © The Author(s)

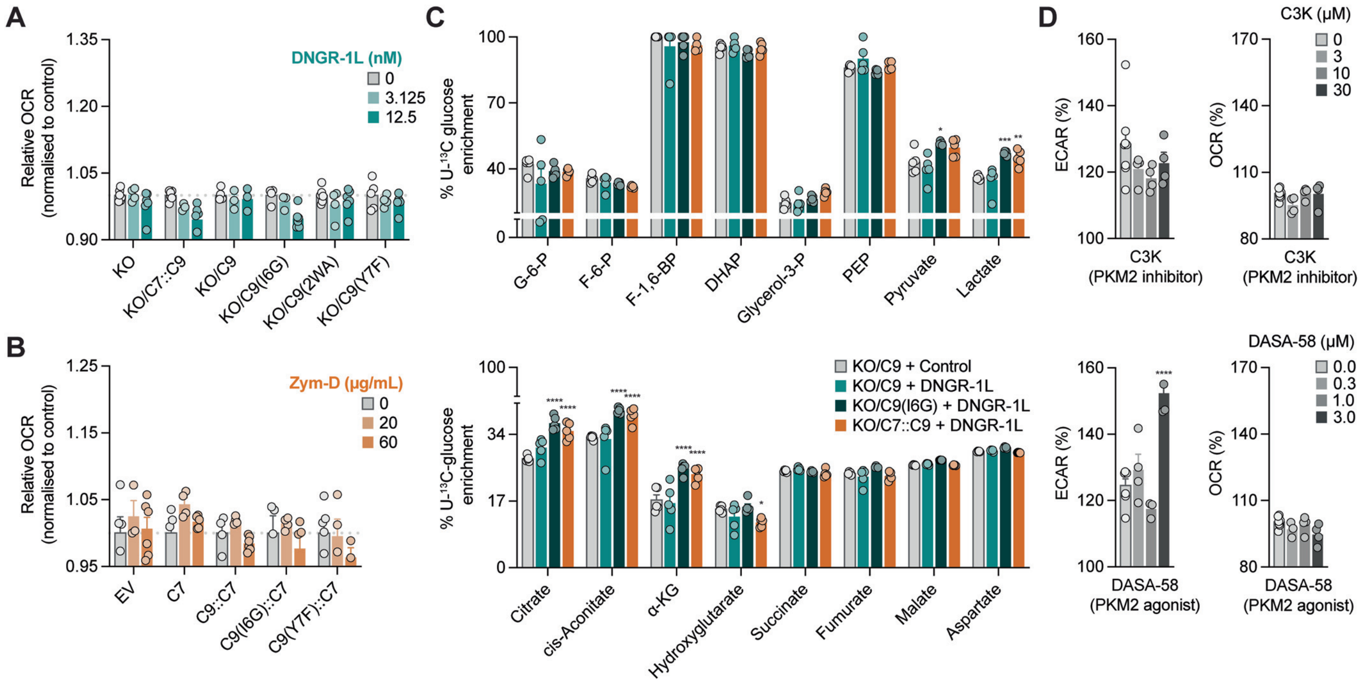

Figure EV3. Assessment of metabolism changes induced by DNGR-1 signalling (related to Fig. 3).

(**A**, **B**) Oxygen consumption rates (OCR) measured at 2 h after ± DNGR-1L injection of (**A**) C9 KO MuTuDCs or those reconstituted with the indicated receptors or (**B**) RAW 264.7 cells ectopically expressing the indicated receptors or EV injected ± Zym-D, $n = 3$–6 per group. Data normalised to baseline measurement immediately after injection with stimuli. Mean ± SEM relative to untreated samples is plotted. (**C**) Fractional labelling of glycolytic (top) or tricarboxylic acid cycle (bottom) metabolites in C9 KO MuTuDCs reconstituted with indicated receptors and stimulated ± 12.5 nM DNGR-1L cultured with uniformly-labelled U-^{13}C-glucose introduced at the time of stimulation. Data shown as mean ± SEM from five biological replicates. (**D**) Extracellular acidification rate (ECAR) and OCR of MuTuDCs treated for 2 h with C3K (PKM2 inhibitor) or DASA-58 (PKM2 agonist). Data normalised as in (**A**). Mean ± SEM is plotted ($n = 4$ per treatment and 9 for untreated samples). Data are representative of two (**A**, **B**, **D**) independent experiments. Data were analysed using Tukey-corrected two-way ANOVA with significant values comparing against untreated controls plotted (**A–D**). (**C**) (top) $^*P = 0.0128$, $^{**}P = 0.0084$, $^{***}P = 0.0006$; (bottom) $^*P = 0.0136$, $^{****}P < 0.0001$, (**D**) $^{****}P < 0.0001$.

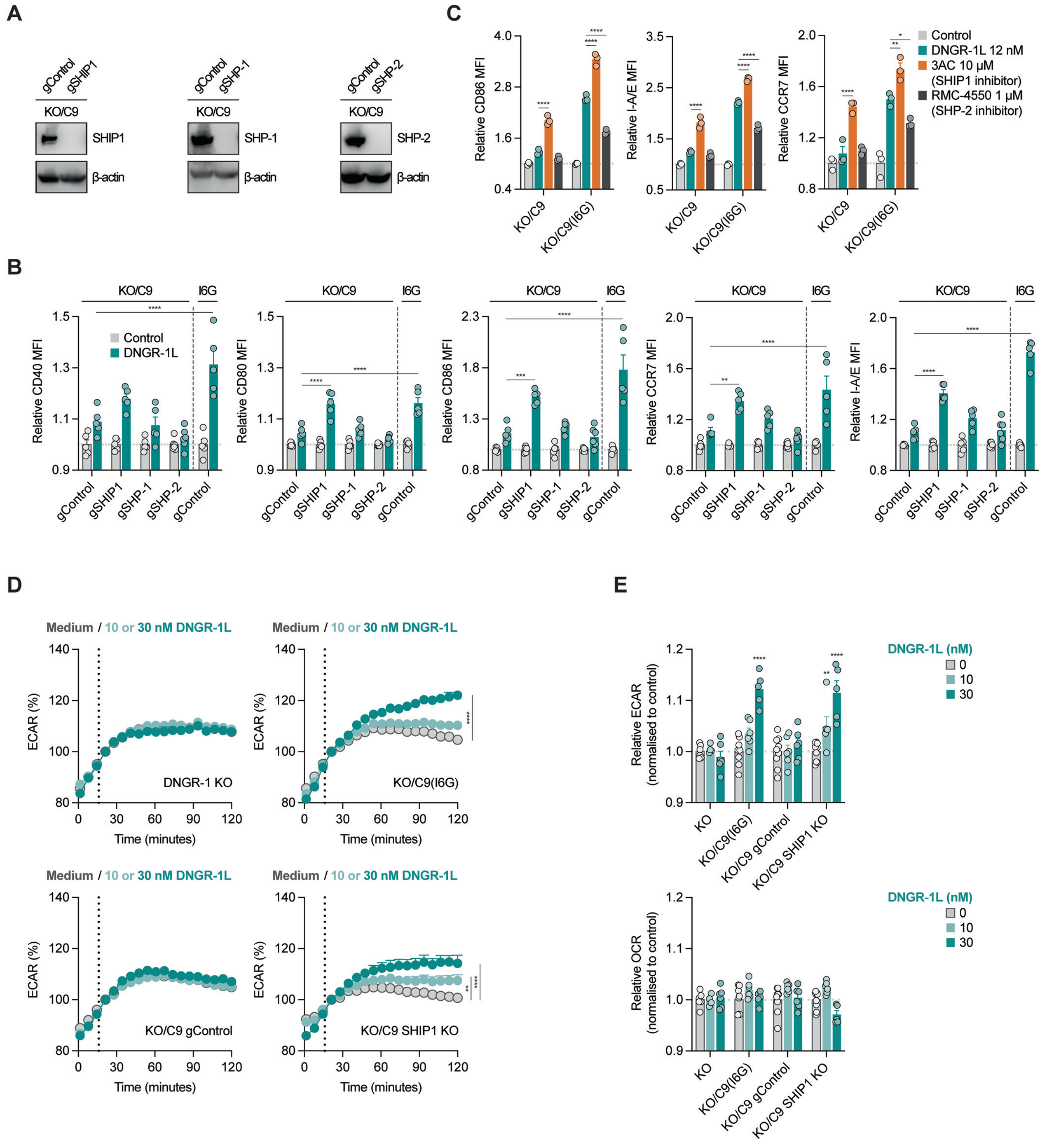
A
gControl
gSHIP1
KO/C9
SHIP1
β-actin
gSHP-1
SHP-1
gSHP-2
SHP-2
B
KO/C9
I6G
Relative CD40 MFI
Relative CD80 MFI
Relative CD86 MFI
Relative CCR7 MFI
Relative I-A/E MFI
Control
DNGR-1L
C
Control
DNGR-1L 12 nM
3AC 10 μM (SHIP1 inhibitor)
RMC-4550 1 μM (SHP-2 inhibitor)
KO/C9(I6G)
D
Medium / 10 or 30 nM DNGR-1L
ECAR (%)
Time (minutes)
DNGR-1 KO
KO/C9(I6G)
KO/C9 gControl
KO/C9 SHIP1 KO
E
Relative ECAR (normalised to control)
Relative OCR (normalised to control)
DNGR-1L (nM)
KO
KO/C9 gControl
KO/C9 SHIP1 KO

Figure EV4. SHIP1 inhibition rescues DNGR-1 mediated cDC1 activation. (related to Fig. 4).

(**A**) Western blot analysis of C9 KO MuTuDCs reconstituted with C9 made deficient for target proteins using CRISPR/Cas9. Two guide (g) RNAs that target SHIP1 (gSHIP1), SHP-1 (gSHP-1), SHP-2 (gSHP-2) or scrambled sequences (gControl) were used to complex with recombinant Cas9 protein for nucleofection. (**B**) Flow cytometric analysis of surface marker MFIs detected from KO/C9, KO/C9(I6G) or KO/C9 SHIP1, SHP-1, or SHP-2 deficient (KO) MuTuDCs cultured overnight ± 12.5 nM DNGR-1L. Mean ± SEM relative to untreated (Control) samples from biological replicates pooled from two independent experiments is plotted. (**C**) Flow cytometric analysis of surface protein MFIs from C9 KO MuTuDCs reconstituted with C9 or (I6G) MuTuDCs stimulated ± 12.5 nM DNGR-1L alone or in the presence of SHIP1 inhibitor 3AC or SHP-2 inhibitor RMC-4550 overnight. Mean ± SEM relative to untreated (Control) samples from biological triplicates is plotted. (**D**, **E**) Extracellular acidification rate (ECAR, indicator of glycolysis) measured at baseline and after ± 10 or 30 nM DNGR-1L injection of C9 KO SHIP1 sufficient and deficient MuTuDCs reconstituted with indicated receptors. $N = 4$–9 per group. (D) Data normalised to baseline measurement immediately after injection with stimuli and shown as % of baseline. Mean ± SEM is plotted. (**E**) ECAR at 2 h post-treatment. Data normalised as in (**C**). Mean ± SEM is plotted. Data are representative of one (**C**) or two (**A**, **B**, **D**, **E**) independent experiments. Data were analysed using Tukey-corrected two-way ANOVA. Comparisons are indicated (**B**, **C**) or against untreated controls (**D**, **E**) with significant values plotted. (**B**) $^{**}P = 0.0011$, $^{****}P < 0.0001$, (**C**) $^{*}P = 0.0177$, $^{**}P = 0.0029$, $^{****}P < 0.0001$, (**D**) $^{**}P = 0.0071$, $^{****}P < 0.0001$, (**E**) $^{**}P = 0.0071$, $^{****}P < 0.0001$.

© The Author(s)

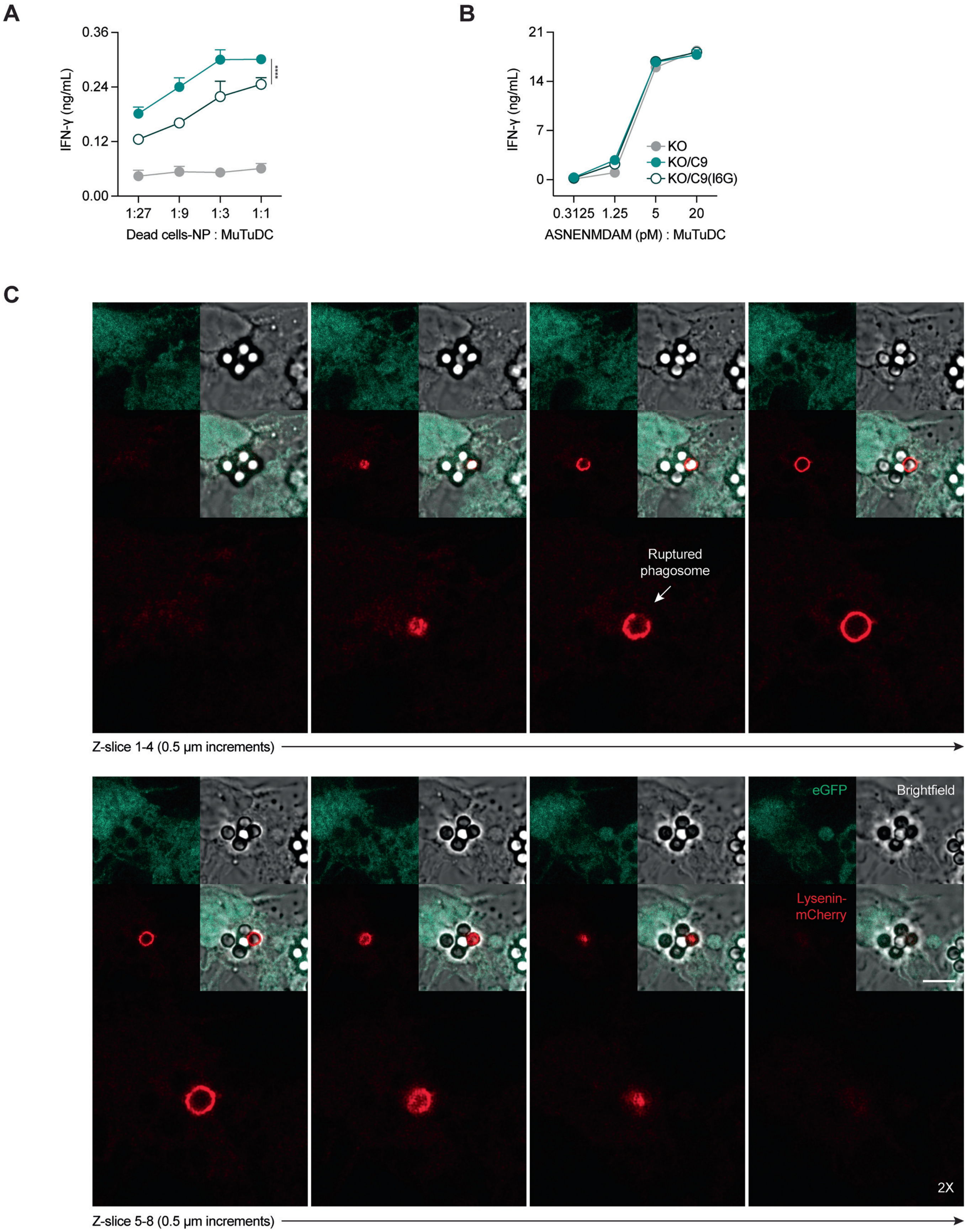
A
0.36
0.24
0.12
0.00
IFN-γ (ng/mL)
1:27
1:9
1:3
1:1
Dead cells-NP : MuTuDC
****
B
21
14
7
0
IFN-γ (ng/mL)
0.3125
1.25
5
20
ASNENMDAM (pM) : MuTuDC
KO
KO/C9
KO/C9(I6G)
C
Ruptured phagosome
Z-slice 1-4 (0.5 µm increments)
eGFP
Brightfield
Lysenin-mCherry
2X
Z-slice 5-8 (0.5 µm increments)

 © The Author(s)

Figure EV5. Cross-presentation of NP antigen and phagosomal rupture (related to Fig. 5).

(**A**, **B**) IFN-γ release from F5 T_E cells co-cultured with C9 KO MuTuDCs reconstituted or not with the indicated receptors and incubated with (**A**) NP-expressing dead cells, or (**B**) ASNENMDAM peptide. Mean ± SD from biological triplicates is plotted. (**C**) Confocal microscopic analysis of lysenin-mCherry fusion protein-expressing KO/C9 MuTuDCs co-cultured with α-DNGR-1 IgG-coupled beads. 0.5 μm increments of consecutive Z-slices of the same image in Fig. 5H. Arrow indicates ruptured area of lysenin-mCherry^{+} phagosome indicated by loss of mCherry signal. Scale bar = 2 μm. Data are representative of two independent experiments (**A**–**C**). Data were analysed using Tukey-corrected two-way ANOVA. Comparisons are indicated (**A**, **B**) with significant values plotted. (**A**) ****P < 0.0001.

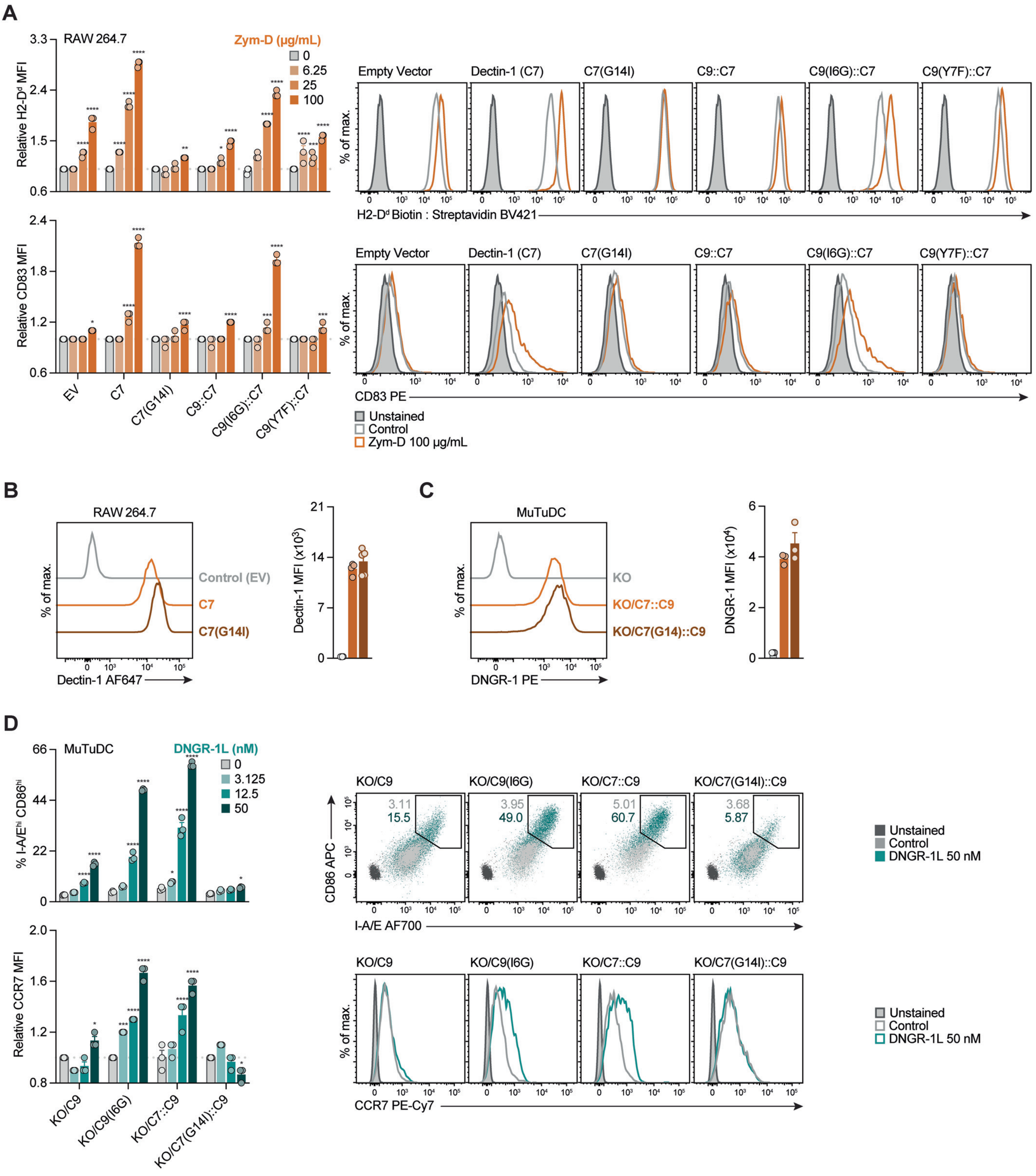
A
RAW 264.7
Zym-D (µg/mL)
0
6.25
25
100
Relative H2-Dᵈ MFI
Relative CD83 MFI
EV
C7
C7(G14I)
C9::C7
C9(I6G)::C7
C9(Y7F)::C7
Empty Vector
Dectin-1 (C7)
% of max.
H2-Dᵈ Biotin : Streptavidin BV421
CD83 PE
Unstained
Control
Zym-D 100 µg/mL
B
RAW 264.7
Control (EV)
C7
C7(G14I)
Dectin-1 AF647
Dectin-1 MFI (x10³)
C
MuTuDC
KO
KO/C7::C9
KO/C7(G14)::C9
DNGR-1 PE
DNGR-1 MFI (x10⁴)
D
MuTuDC
DNGR-1L (nM)
0
3.125
12.5
50
% I-A/Eʰⁱ CD86ʰⁱ
Relative CCR7 MFI
KO/C9
KO/C9(I6G)
KO/C7::C9
KO/C7(G14I)::C9
CD86 APC
I-A/E AF700
CCR7 PE-Cy7
DNGR-1L 50 nM

 © The Author(s)

Figure EV6. Cell lines to analyse Dectin-1 function (related to Fig. 6).

(**A**) Flow cytometric analysis of the indicated surface marker expression by RAW 264.7 cells ectopically expressing indicated receptors or transduced with EV and stimulated overnight ± Zym-D. Mean ± SEM from biological triplicates (left) and representative histograms (right) are plotted. Note that these data are the same data as in Fig. 6A, with bar graphs plotted here as MFI relative to untreated controls to better emphasise the response to Zym-D. Dotted line represents 1. (**B**) Flow cytometric analysis of surface Dectin-1 (C7) expression in RAW 264.7 cells ectopically expressing the indicated receptors or transduced with EV. Cell lines were established after sorting for equal expression of Dectin-1. (**C**) Flow cytometric analysis of surface DNGR-1 (C9) expression in C9 KO MuTuDCs reconstituted or not with the indicated chimeric receptors. Cell lines were established after sorting for equal expression of DNGR-1. (**B**, **C**) Representative histograms (left) and mean MFI ± SEM (right) are plotted from biological (**B**) quintuplets or (**C**) triplicates. (**D**) Flow cytometric analysis of surface marker expression by C9 KO MuTuDCs reconstituted or not with indicated receptors stimulated overnight ± DNGR-1L. Mean ± SEM from biological triplicates (left) and representative histograms (right) are plotted. CCR7 is shown relative to untreated controls and the dotted line represents 1. Note that these are data from the same experiments as in Fig. 6B, plotted differently. Data are representative of two (**B**, **C**), or three (**A**, **D**) independent experiments. Data were analysed using Tukey-corrected two-way ANOVA with significant values comparing against untreated samples plotted (**A**–**D**). (**A**) H2-D^d *$P = 0.0405$, **$P = 0.0011$, ***$P = 0.0007$, ****$P < 0.0001$; CD83 *$P = 0.0374$, ***$P = 0.0001$, ****$P < 0.0001$, (**D**) I-A/E^{hi} $CD86^{hi}$ *$P = 0.0352$ (KO/C7::C9), $P = 0.0468$ (KO/C7(G14I)::C9), ****$P < 0.0001$; CCR7 *$P = 0.0240$, ***$P = 0.0004$, ****$P < 0.0001$.
